# Orientational enhancement of surface phonon polarity in superconducting KTaO₃ interfaces

**DOI:** 10.1038/s41467-026-76343-4

**Published:** 2026-08-14

**Authors:** Tongying Liu, Jiashi Li, Shiyu Zhang, Tao Zhou, Fanjin Lu, Xiaoyang Chen, Jia Liu, Long Cheng, Xinyi Liu, Wei Li, Yanwu Xie, Xiaofang Zhai, Rui Peng, Wei-Tao Liu

**Affiliations:** 1 Physics Department, State Key Laboratory of Surface Physics, Key Laboratory of Micro and Nano Photonic Structures [Ministry of Education (MOE)], Fudan University, Shanghai, 200433 China; 2 School of Physical Science and Technology, Shanghai Tech University, Pudong, Shanghai, 201210 China; 3 School of Physics, and State Key Laboratory for Extreme Photonics and Instrumentation, Zhejiang University, Hangzhou, 310027 China

**Keywords:** Surfaces, interfaces and thin films, Nonlinear optics, Superconducting properties and materials

## Abstract

The critical role of symmetry-breaking interfaces in mediating superconductivity has garnered increasing interest in recent years. KTaO_3_ (KTO)-based heterointerfaces present a striking example, where the superconducting transition temperature (*T*_*c*_) exhibits an intriguing dependence on KTO crystallographic orientation. Here we identify a highly anisotropic surface phonon resonance near 107 meV at KTO and KTO-based heterointerfaces using surface-specific, symmetry-sensitive nonlinear optical spectroscopy. Polarity of this phonon mode scales with crystallographic orientation as (111) > (110) ≫ (001), reaching a 35-fold enhancement over non-superconducting interfaces and tracking the superconducting *T*_*c*_. In addition, a softened local mode near 100 meV is observed and attributed to interfacial oxygen vacancies. Our results help build a framework linking the symmetry-breaking lattice phonons, defects, and anisotropic superconductivity for KTO and related heterointerfaces.

## Abstract (web summary)

Nonlinear optical spectroscopy reveals a highly anisotropic polar surface phonon at KTaO3-based heterointerfaces, strongest for the superconducting (111) orientation. The phonon response tracks superconducting Tc, linking interfacial symmetry breaking, defects and anisotropic superconductivity.

## Introduction

The discovery of emergent phenomena at correlated oxides interfaces has attracted growing interest, for bringing new opportunities to the next generation device applications^1–4^. At these interfaces, the quantum confinement of correlated electrons and broken symmetry with reduced dimensionality unlocks exotic phenomena, such as superconducting two-dimensional electron gas (2DEG) systems^5,6^, electrically tunable Rashba spin-orbit coupling (SOC)^7–12^, emergent interfacial ferromagnetism^13–15^ and anomalous Hall effects^16–18^, and so on. Central to these phenomena is the intricate interplay between charge, spin, orbital, and lattice degrees of freedom. In particular, longitudinal optical (LO) phonons have recently been identified as key mediators of emergent phenomena through strong electron-phonon coupling (EPC)^19–22^. The inversion symmetry breaking at polar interfaces provides additional tunability by generating giant static interfacial fields (∼10^9 ^V/m)^23–26^ which can amplify Rashba-type SOC through relativistic effects. Such a static field may influence the interfacial EPC landscape by modifying the equilibrium polar structure^25,27^, the dielectric response and carrier screening^28,29^, and the confinement and spatial distribution of interfacial electronic states^30,31^ thereby changing their overlap with polar-phonon fields^32^.

KTaO_3_ (KTO), a prototypical quantum paraelectric, has served as an excellent platform for studying low-dimensional electron systems due to its large dielectric constant and high carrier mobility^33–36^. Compared to its lighter counterpart, SrTiO_3_ (STO), KTO hosts Ta-5*d* orbitals near the Fermi level, which can synergize with interfacial polarity to generate exceptionally strong surface fields and enhanced SOC [ ~ 400 meV at G-point, 20× larger than STO]^10,37^. While the STO-based heterostructures, such as LaAlO_3_ (LAO)/STO, exhibit superconductivity only below ~200 mK^6^, the LAO/KTO system showcases an intriguing orientation-dependent superconductivity, with the transition temperature (*T*_*c*_) being ~2 K for the KTO-(111) substrate, 0.9 K for (110), and absent for (001) (down to 25 mK)^31,38,39^. This positions KTO-based oxide heterostructures as a unique platform to elucidate the roles played by crystalline symmetry, phonon dynamics, and interfacial polarity in the Cooper pairing mechanism.

Currently, our understanding of such effects is still emerging, for it requires integrating information from both electronic and lattice degrees of freedom, with high surface/interface specificity. The second-order nonlinear optical technique such as sum-frequency spectroscopy (SFS), can help bridge this gap^40–42^. Compared to angle-resolved photoemission spectroscopy (ARPES)^32^ and scanning tunneling microscopy (STM)^43^, SFS is capable of accessing the buried interfaces between different oxide subphases, whether conducting or insulating. It also detects vibrational fingerprints of interfacial lattices and defects with sub-meV resolution^40^. Compared to conventional Raman scattering for bulk studies, SFS has the same inherent sensitivity to electron-phonon couplings^44,45^, and further inherent specificity to symmetry-breaking surfaces and interfaces^40^. Combined, SFS has been successfully established for studying a variety of quantum material surfaces and interfaces^46–49^.

In this work, we employ SFS to study the surface phonons of KTO and KTO-based heterostructures. Two major resonant modes were identified in situ: a prominent one at ~107 meV derived from the bulk LO_4_ mode, and a defect-related mode at ~100 meV. Using susceptibility tensor analysis, we quantify the surface phonon polarity across different KTO crystallographic orientations, revealing an orientation-dependent static interfacial polar field that can enhance Rashba-type SOC and modulate the interfacial EPC landscape via associated polar reconstruction. Intriguingly, the surface phonon polarity is dramatically enhanced with the establishment of 2DEG at amorphous-LAO (*a*-LAO)/KTO interfaces, following the trend (111) > (110) ≫ (001), which was in direct correlation with the superconducting transition temperature *T*_*c*_. Besides, low-temperature ultraviolet (UV) irradiation studies indicated an elevated oxygen vacancy (V_O_) concentration on (111) surface, which might further contribute to the emergent conductivity. Our findings can contribute to the establishment of a general framework for relating interfacial symmetry, phonon-mediated electron-electron correlation, and defect engineering in oxide quantum materials.

## Results

### SF spectra from KTO substrates

**Fig. 1:**
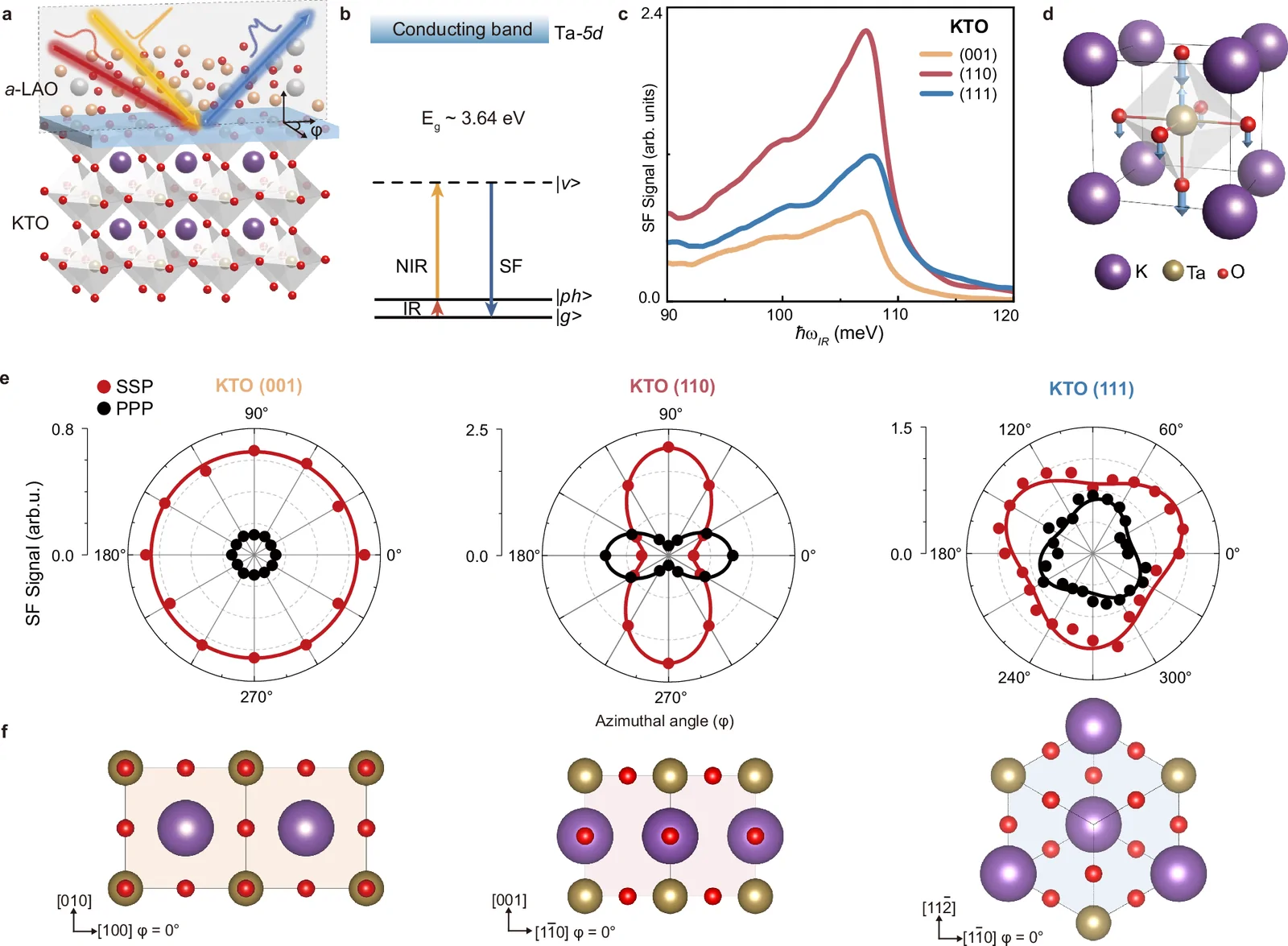
Sum-frequency surface phonon spectroscopy of KTO. **a** Schematics of the SFG experimental geometry, with IR and NIR beams overlapping and generating the SF beam from the symmetry-broken interface. **b** Transition diagram of the SFG process in KTO. The IR photon excites a phonon coherence from the ground state, upconverted by the NIR photon at 1.5 eV to a virtual state ( | *v* > ) and emits an SF photon. The Ta-5*d* states constitute the bottom of the conduction band with the gap 3.64 eV. **c** SF spectra with respect to the IR photon energy from KTO substrates with (001), (110), and (111) orientations. **d** The normal mode coordinates of the LO_4_ phonon in a TaO_6_ octahedron of bulk KTO. Blue arrows represent displacements of ions, with arrow length indicating the displacement amplitudes (not to scale). **e** Azimuthal anisotropy patterns of SF signals averaged within 107 ± 1 meV from samples in **c**. SSP: S-polarized SF, S-NIR, P-IR; PPP: P-polarized SF, P-visible, P-infrared. The solid curves are fits using equations in the Supplementary Information. **f** Atomic configurations of the three surfaces viewed from the top.

The principle of SFS is provided in detail elsewhere^40^. The SFS experiments were done with a femtosecond broadband laser system^48^ (see details in Methods and Supplementary Information). The narrowband near-infrared (NIR) and broadband infrared (IR) pulses overlapped both spatially and temporally at the sample surface (Fig. 1a), and the sample could be rotated azimuthally (*φ*) around the surface normal direction to acquire anisotropy patterns. As illustrated in Fig. 1b, the quantum process of SFS for phonon spectroscopy involves the adsorption of an infrared (IR) photon from the ground state ( | *g*˃) that excites the phonon coherence ( | *ph*˃), and the subsequent up-conversion by another photon [at NIR ~ 1.5 eV in our case] to a virtual state ( | *v*˃), then the emission of the SF photon. The Ta-5*d* states, constituting the bottom of the conduction band, serve as the closest electronic levels to this virtual state. Due to the mutually exclusive Raman and IR selection rules in centrosymmetric systems, the sum frequency generation (SFG) is only active at the symmetry-breaking surfaces and interfaces under the electric dipole approximation^40^. Figure 1c shows representative SSP (S-polarized SF beam, S-pol. NIR, and P-pol. IR) SFG spectra from (001), (110), and (111)-oriented KTO bare substrates, acquired at optimal geometries with the maximum signal intensity. Azimuthally averaged data and corresponding spectra taken with another polarization combination (PPP) are provided in Supplementary Fig. 1. In all cases, a prominent resonance mode near 107 meV was observed.

To assign this mode, we note that the mode is adjacent to the bulk LO_4_ mode at ~104 meV^50,51^, which is the highest longitudinal optical phonon at Γ-point. This triply degenerate phonon mode transforms according to the *T*_*1u*_ (*Г*_*15*_) irreducible representation of the O_*h*_ group. It involves antiphase displacements of tantalum atoms and oxygen octahedra and has optical transition dipole moments along three Ta-O bonds, as illustrated in Fig. 1d. Similar to the case of STO^48^, the projected modes of LO_4_ to surfaces become SFG-active due to the broken inversion symmetry. Notably, the surface phonon intensity is highly orientation-dependent. Another notable spectral feature was a broad shoulder-like band around ~100 meV, which is associated with local structural defects^48^ and would be discussed in detail later.

The spectral intensity at varying azimuthal angle (*φ*) are depicted in Fig. 1e. Here, *φ* = 0° for the (001), (110) and (111) surfaces are defined when the beam incident plane was aligned with the [100], [1$\bar{1}$0], and [1$\bar{1}$0] directions, respectively (see coordinate systems in Fig. 1f). Details on the extraction of the anisotropic patterns are provided in Supplementary Information. As expected, the patterns agreed with the structural symmetry of each surface, that is *C*_*4v*_ for (001), *C*_*2v*_ for (110) and *C*_*3v*_ for (111) surfaces. Atomic configurations of each surface are presented in Fig. 1f (top view).

### Signal enhancement at heterointerfaces

**Fig. 2:**
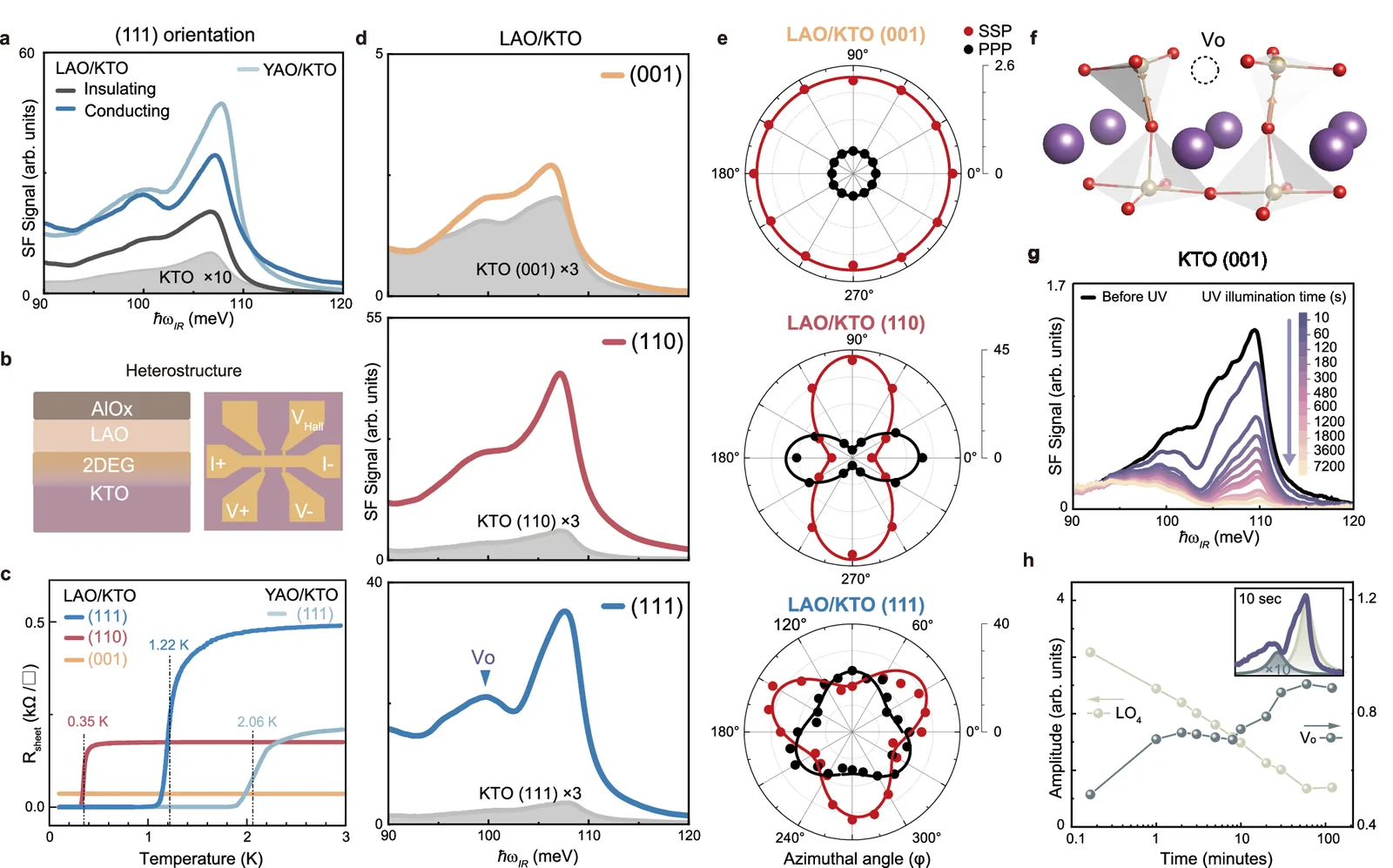
SF surface phonon spectra from KTO-based heterointerfaces. **a** SF spectra from KTO (111) orientation: *a*-LAO/KTO insulating (black) & conducting (blue), *a*-YAO/KTO (light blue), bare KTO (gray filled, amplified by 10 times). **b** Sketches of the AlO_x_/LAO/KTO heterostructure and the Hall-bar device. **c** Temperature-dependent sheet resistance of LAO/KTO samples with (001), (110) and (111) orientations and YAO/KTO(111) adapted from refs. ^12,37^. **d** SF spectra (colored) for samples in (**c**) and corresponding KTO substrates (gray filled, amplified by 3 times). The vertical axes for all three panels are to scale and numerically comparable. **e** Azimuthal anisotropy patterns of SF signals. **f** Illustration of the local vibration mode around V_O_, with the displacements of Ta and O ions marked by orange arrows. The neighboring TaO_6_ octahedra are distorted due to the change in bonding strength. **g** Spectral dependence on accumulated UV illumination time at 60 K [from KTO (001) substrate]. **h** Amplitude evolution of LO_4_ and Vo modes over accumulated UV illumination time. Insert: fitting of the spectrum at 10 second.

The deposition of *a*-LAO layer led to drastic changes in the SF phonon spectra, as shown in Fig. 2. Since the optical dispersion of LAO within this range is small^52^, the spectral modulation caused by the nm-scale LAO capping layers is negligible (details in Supplementary Note 4, Supplementary Fig. 12). First, we monitored the case with different LAO thicknesses. With a 1.2 nm-LAO layer that is below the 2DEG formation threshold, the spectrum (black curve in Fig. 2a) showed the same 107 meV resonance as bare KTO (111) substrates (gray filled curve, amplified by 10 times), but the intensity was enhanced by nearly 20 times. Next, we increased the *a*-LAO thickness to 2 nm that triggered the 2DEG formation, and the phonon intensity was further enhanced (blue curve) to about 33 times of the bare substrate. This behavior starkly contrasts with the LAO/STO interface, where the 2DEG formation was found to suppress the surface phonon intensity^48^, highlighting distinct interfacial physics between STO and KTO heterostructures. To further confirm that the enhanced mode originates from KTO, we prepared *a*-YAlO_3_/KTO(111) sample (*T*_*c*_ = 2 K), and found the same strong enhancement of the 107 meV mode compared to the bare KTO substrate (light blue curve, Fig. 2a). The fitting parameters for the spectra shown in Fig. 2a are provided in Supplementary Table 8. More spectra are shown in Supplementary Fig. 2.

This enhancement in surface phonon intensity exhibited significant crystallographic anisotropy. Figure 2b presents the structure of AlO_x_ protected *a*-LAO grown on KTO (001), (110), and (111) substrates, with corresponding transport curves in Fig. 2c. The (111) and (110) samples showed superconducting transitions at *T*_*c*_ = 1.22 and 0.35 K, respectively. For the (001) sample, no transition was observed down to our base temperature limit of 0.1 K (in ref. ^36^, a *T*_*c*_ of ~ 50 mK was found). The surface phonon intensities from all heterostructures were enhanced from the corresponding bare substrate, but dramatically different in magnitudes: (001) (Fig. 2d, top panel, vertical axis adjusted to magnify the spectrum) exhibited a 4-fold increase relative to the bare substrate (gray filled), while (110) and (111) surfaces showed over 33- and 35-fold enhancement, respectively (middle/bottom panels). For conducting (111) interfaces, 1- and 2-nm LAO capping layers yielded about the same enhancement. All spectra were acquired at azimuthal angles with the maximum intensities, with the trends of enhancement persisted in azimuthally averaged data (Supplementary Fig. 3). Meanwhile, the linear dependence on both IR and NIR powers confirmed that the SF signal originated from second-order nonlinear responses, with negligible effects from laser-induced heating or sample degradation (Supplementary Fig. 11). The reproducibility of SF spectra over the data acquisition period was also routinely checked to rule out long-term sample degradation.

Measurements from additional batches of samples confirmed the above result, that the LAO/KTO interfaces yielded enhanced SF phonon signals from corresponding bare KTO surfaces, with the magnitude of enhancement being much greater for (110) and (111) surfaces than for the (001) surface. Meanwhile, the azimuthal orientational dependences (Fig. 2e) remained consistent with the crystalline symmetry, confirming the preservation of local crystallographic symmetry. Overall, this dramatic enhancement of interfacial phonon intensities upon heterostructure formation suggests a close interplay between lattice and electronic degrees of freedom that will be discussed in the following section.

### Oxygen vacancies

Besides the major response derived from the LO_4_ mode, many spectra exhibited a low-frequency “shoulder” around ~ 100 meV. In our previous study on STO^48^, we showed that oxygen vacancies led to distinctive SF response at frequencies lower than the stoichiometric mode. Similar to STO, the KTO surfaces are also prone to form V_O_s, and the adjacent Ta-O bonds could also exhibit softened stretching vibrations (Fig. 2f). To verify the assignment, we irradiated the KTO (001) surface (to avoid the influence of anisotropy) with a 365 nm (3.40 eV) UV light in a vacuum of 10^−7 ^Torr at 60 K, causing migration and accumulation of V_O_s at the surface due to lower formation energy^53,54^. After each irradiation period, five SF spectra were acquired sequentially over a 5-minute window with UV blocked. The stability of consecutive SF spectra ensured that the photothermal effect by UV was negligible. We then averaged all spectra within each data acquisition window.

As shown in Fig. 2g, the spectral profile underwent marked changes upon UV irradiation, and split into two distinctive bands with the spectral weight at the low-frequency side increased steadily. By fitting spectra of different UV dosage, we extracted the evolution of the 107 and 100 meV mode amplitudes with respect to the UV irradiation time, as plotted in Fig. 2h and tabulated in Supplementary Table 1. It is clearly seen that in contrary to the 107 meV, the relative strength of the 100 meV kept increasing with high UV dosage, agreeing with its assignment to local Ta-O vibrations adjacent to V_O_s. After the UV exposure, the dark spectrum remained stable at 100 K over 12 h, and only gradually returned to the original state at elevated temperature or oxygen exposure (Supplementary Fig. 4), consistent with the behavior expected for interfacial V_O_s. V_O_s could elevate the carrier density of 2DEG via doping and introduce disorders as scattering centers, while since it is a relatively minor effect in the current system, we would focus our discussion on the 107 meV mode originating from stoichiometric KTO in the following sections.

### Origin of the SF response

To help understand results from KTO and *a*-LAO/KTO interfaces, we compare to our previous findings on crystalline (*c*-)LAO/STO heterostructures^48^. As we mentioned earlier, the STO and KTO heterostructures exhibited distinct interfacial physics. On pristine STO (001) surface, the 103 meV surface phonon (derived from the bulk LO_4_ mode) intensity dropped sharply upon the deposition of 1 or 2 unit-cell (uc) of *c*-LAO due to the restoration of inversion symmetry, showing that the STO response was highly localized to the topmost surface layer. The signal increased at 3 uc of LAO due to the ferroelectric distortion, then dropped again at 4 uc following the formation of 2DEG.

For KTO, the phonon intensities from pristine surfaces were similar to those of pristine STO. However, upon the formation of 2DEG with LAO or YAO, the KTO phonon intensity did not drop as for LAO/STO, but drastically increased by 4 ~ 35 times from bare substrates. Compared to the STO surface phonon and typical molecular monolayers, the nonlinear optical susceptibility of KTO phonon is one to two orders of magnitude stronger^40^. To understand that, we note that according to ARPES measurements, 2DEGs at KTO interfaces have a quasi-3D nature and distributed in a more extended region ( ~ 5.5 to 6.8 nm) compared to that at STO interfaces^32^. The longer penetration depth of the accompanied surface field could lead to stronger nonlinear optical responses known as the electric-field-induced (EFI) SFG from the bulk subphase^40^. Therefore, the robustness of the phonon frequency and linewidth from case to case indicates that the underlying lattice structures remained stable, without major structural variations and/or chemical degradation. Meanwhile, the rehybridization of electronic states due to the broken symmetry, and symmetry-regulated EPC and/or SOC^8–10^, could modulate the transition matrix elements of the anti-Stokes Raman process in SFG (Fig. 1b)^48^. Besides, the carrier accumulation caused by the surface polar field could also affect the strength of the nonlinear susceptibility tensor elements. In the absence of the microscopic theory on nonlinear optical susceptibility involving the above effects, we are not able to resolve individual contributions at this stage. Still, all these effects could be considered collectively as an effective polarity-enhancement factor that characterizes the degree of symmetry breaking across different interfaces for comparison.

### Susceptibility tensor analysis

Here, we systematically analyze the nonlinear susceptibility tensor elements governing the SF response, which would enable a quantitative comparison of polarities of the three distinct crystallographic interfaces. The workflow for extracting the nonlinear susceptibility tensor elements is explicitly illustrated in Supplementary Fig. 10, with detailed derivations provided in Supplementary Note 2. Since the surface phonon resonances remained at a similar frequency in all cases, the corresponding lattice constants were nearly preserved. We therefore assume the effective ${{\chi }^{\leftrightarrow}}^{\left(2\right)}$ tensors were all derived from the KTO bulk third-order nonlinear optical susceptibility ${{\chi }^{\leftrightarrow}}^{\left(3\right)}$ like that for EFI-SFG, with an effective symmetry-breaking polar field. Since KTO belongs to the O_*h*_ point group, ${{\chi }^{\leftrightarrow}}^{\left(3\right)}$ has 21 non-zero tensor elements ${{\chi }}_{{\alpha }{\beta }{\mu }{\nu }}^{\left(3\right)}\left({\alpha }{\beta }{\mu }{\nu }{{{\rm{\in }}}}a=\left[100\right],b=\left[010\right],c=\left[001\right]\right)$ of which only four are independent, denoted as ${\chi }_{1}$, ${\chi }_{2}$, ${\chi }_{3}$, ${\chi }_{4}$.

1 $${aaaa}\left(={{{{\rm{\chi }}}}}_{{aaaa}}^{\left(3\right)}\right)={bbbb}={cccc}={\chi }_{1}$$

2 $${aabb}={aacc}={bbaa}={bbcc}={ccaa}={ccbb}={\chi }_{2}$$

3 $${abab}={acac}={baba}={bcbc}={caca}={cbcb}={\chi }_{3}$$

4 $${abba}={acca}={baab}={bccb}={caac}={cbbc}={\chi }_{4}$$

It is reasonable to assume that the intrinsic susceptibility ${{\chi }^{\leftrightarrow}}^{\left(3\right)}$ would retain the same form for all three KTO surfaces. By transforming from the crystal to the lab coordinate system, we can deduce the effective susceptibility tensor elements of each surface that contribute to the SSP and PPP spectra. Following the Thomas-Fermi screening model, we modeled the symmetry-broken region as under an effective polar field described by ${{{\bf{E}}}}={E}_{Z}{e}^{-z/\xi }{{{\bf{z}}}}$ (${{{\bf{z}}}}$ being the surface normal and *ξ* the thickness that field extends). Since the beam coherence length in our measurements was ~30 nm^48^, it fully covered the 2DEG thickness of a few to ten nanometers estimated by ARPES studies^32^. Therefore, the effective second-order nonlinear optical susceptibility ${{\chi }^{\leftrightarrow}}^{\left(2\right)}$ measured in SFG takes the form ${{\chi }}_{B,i}^{\left(2\right)}={d}_{i}{E}_{z}\xi$. The ${{\chi }^{\leftrightarrow}}^{\left(2\right)}$ components can then be independently derived through fitting of SSP and PPP spectra and patterns, using the Fresnel-related coefficients provided in Supplementary Table 6 (see detailed derivation in Supplementary Note 2). The results are tabulated in Supplementary Tables 2–4. Assuming ${{\chi }^{\leftrightarrow}}^{\left(3\right)}$ being invariant, the variation in ${{\chi }^{\leftrightarrow}}^{\left(2\right)}$ between different KTO surfaces and interfaces thus corresponds to the variation in surface polarities.

### Orientational polarity enhancement

**Fig. 3:**
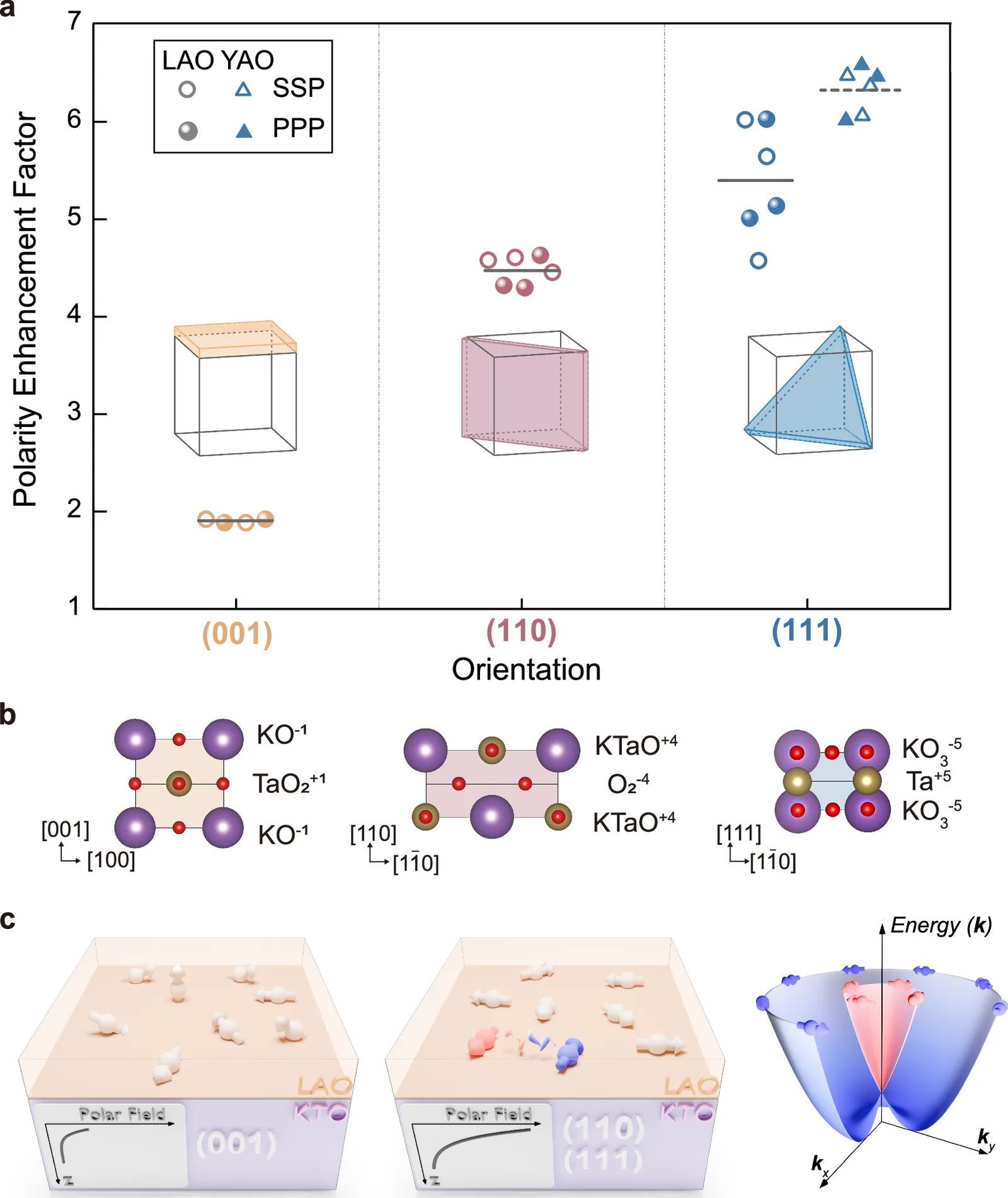
Orientational dependent phonon polarity enhancement factor. **a** The polarity enhancement factors obtained independently from SSP (open symbols) and PPP (filled symbols) spectra and anisotropy patterns for LAO/KTO (circles) and YAO/KTO (triangles) heterointerfaces. Data points from various measurement sets are horizontally offset for clarity, with black bars indicating the average values. **b** Atomic configurations of the three surfaces viewed from the side, with layer charges denoted. **c** Illustration of the cases with weaker (left panel) and stronger (middle panel) polar fields and Rashba effects.

We define the polarity enhancement factor by the ${{\chi }^{\leftrightarrow}}^{\left(2\right)}$ ratio between that of hetero-interfaces and corresponding KTO bare surfaces, as tabulated in Supplementary Table 5 and plotted in Fig. 3a. The open and solid symbols present values independently derived from SSP and PPP measurements, showing a consistent trend of (111) > (110) ≫ (001). For all three orientations, the growth of LAO layers enhances the polar fields. While the (001) interfaces exhibited a moderate enhancement of ~1.90 ± 0.02, the (110) and (111) interfaces showed dramatic enhancements of 4.48 ± 0.13, 5.40 ± 0.54 (for LAO) and 6.35 ± 0.63 (for YAO), respectively, in clear correlation with *T*_*c*_ of each interface. It is noted that this correlation does not ensure causality, which awaits further analysis.

## Discussion: polarity enhancement and superconductivity anisotropy

To understand this orientation-dependent polarity enhancement, we again compare the KTO-based interfaces to those based on STO. While all atomic layers at STO (001) surface are electrically neutral, those of KTO are charged for all three crystalline orientations. As depicted in Fig. 3b, the KTO (001) surface is stacked from alternating (TaO_2_)^+1^ and (KO)^−1^ layers, the (110) surface from (KTaO)^+4^ and O_2_^−4^, and (111) surface from Ta^+5^ and (KO_3_)^−5^ layers, respectively. According to first-principles calculations^55^, higher densities of charges at the (110) and (111) terminations could result in higher polarizability and increased surface charge accumulation upon truncation. For a quick check, we estimated the dipole moments per unit cell of KTO surfaces using nominal charges. This resulted in 1.1, 4.3, and 4.2 *e*·Å for (001), (110), and (111) surfaces, respectively, which roughly followed the trend of polarity enhancement. To be more quantitative, we considered the dynamical Born effective charges linked to the polarization changes induced by atomic movements^56,57^ (details provided in Supplementary Note 3). For the surface phonon mode derived from LO_4_, the corresponding dipole moments for (001), (110), and (111) surfaces were estimated to be 3.6, 10.5, and 10.1 *e*·Å, respectively, as summarized in Supplementary Table 7. Again, it consistently yielded the dipole moments of (111) and (110) surfaces to be 3 ~ 4 times greater than that of the (001), following the same trend of the polarity enhancement detected experimentally.

The strong polarity enhancement at the *a*-LAO-covered KTO (111) and (110) interfaces, specifically for the LO_4_-derived surface phonon mode, indicates a substantially reconstructed interfacial polar environment that may influence the electronic properties via various mechanisms^25,29,31,32^. For the (001) interfaces, the *T*_*c*_ ~ 0.05 K could only be achieved upon a threshold gate voltage^36^, also showcasing the importance of polarity. The LO_4_ mode involving Ta-O anti-phase vibrations (Fig. 1d) can mediate the long-range electron-electron attraction (proportional to the LO/TO splitting) via the generalized Fröhlich mechanism^21,22^. Theoretical studies also demonstrated that surface charge density is maximized on (111) and (110) interfaces and predicted the existence of strong polar fields due to higher Born effective charges and stronger polarities^55,58^. This mechanism complements the deformation-potential interaction arising from mixed ionic-covalent Ta-O bond vibrations, providing both long- and short-range attractive channels for electrons. Consistently, ARPES studies report the weakest EPC signatures on (001), where our SFG measurements also show the lowest surface polarity, suggesting a possible correlation with its low *T*_*c*_.

In addition, the strong polar fields could enhance the Rashba-type SOC^7^ at the interfaces (Fig. 3c). This may contribute to the formation of anisotropic spin textures and orbital hybridization^59,60^, particularly among the three *t*_*2g*_ orbitals, which could promote inter-orbital hopping and further stabilize electron pairing. Specifically, the tetragonal symmetry on (001) lowers the *d*_*xy*_ orbital below *d*_*xz/yz*_ and suppresses inter-orbital scattering channels needed for pairing^35^. Overall, we obtained an empirical correspondence between *T*_*c*_ and enhanced polarity for different KTO samples, which can reflect the combined effects of SOC and EPC, and underscore the potential role of orientation-dependent symmetry breaking, both in-plane and out-of-plane, in devising oxide heterostructures.

Our SFG phonon spectroscopy offers fresh insight into the KTO and *a*-LAO/KTO interfaces. The surface phonon mode near ~107 meV, derived from the LO_4_ mode of the KTO, showed relatively small differences on KTO substrates of different orientations; while with the deposition of *a*-LAO/KTO, the phonon intensities grew sharply, specific to the (110) and (111) interfaces, being enhanced by over 30-fold. Through susceptibility tensor analysis, we found the enhancement of phonon polarity follows the order of (111) > (110) » (001). Meanwhile, through low-temperature UV experiments, we detected a higher concentration of V_O_s on the (111) surface, which could contribute to its elevated carrier density and transition temperature. Such contrasts show an orientation dependence synchronizing the superconducting anisotropy, suggesting that interfacial polarity and defects are closely related to the electronic environment of KTO-based heterointerfaces. Future ultrafast time-resolved studies may provide more insight into the dynamic interaction between electronic and lattice degrees of freedom.

## Methods

### Sample preparation and characterization

We prepared two batches of *a*-LAO/KTO samples: LAO/KTO (111) samples with varying thicknesses, and AlO_x_/LAO/KTO samples with (111), (110), and (001) orientations. In the first batch, the KTO substrates were annealed at 650°C for 2 h in air, then immersed in deionized water at 60°C for 30 min to ensure a highly uniform step-terrace structure and surface termination. Subsequently, LAO thin films were deposited by pulsed laser deposition (PLD) at an oxygen pressure of 5×10^−7 ^mbar, with the growth rate initially calibrated by X-ray reflectivity. Then, the thicknesses of the LAO films were precisely controlled by adjusting the number of laser pulses. The 1.2-nm LAO/KTO sample was insulating with a resistance exceeding 1 GΩ, and the 2 nm-LAO/KTO sample was conducting. In the second batch, all samples featured the 1.5 nm-AlO_x_/1 nm-LAO/KTO hetero-structure taken from ref. ^32^. Prior to film growth, the bare KTO (001), (110), and (111) substrates were treated at 650–680°C in a mixed atmosphere of 1×10^−5^ mbar O_2_ and 1×10^−7^ mbar H_2_O vapor. The *a*-YAlO_3_ /KTaO_3_(111) sample was shown in ref. ^12^ with *T*_*c*_ = 2 K. For this sample, the KTO substrate was initially annealed at 600 °C for 30 minutes in an ultrahigh-vacuum environment to obtain a smooth, uniformly terminated surface prior to deposition. The rms roughness of the KTO surfaces was typically around 1 ~ 2 Å as probed by AFM^12,32,38,61^.

### Laser experimental details

The experiment employed a Ti:sapphire femtosecond laser system (Spectra Physics), which consists of four main components, as shown in Supplementary Fig. 5. First, the oscillator (MaiTai SP) and regenerative amplifier (Spitfire Pro) generated an 800 nm 35 fs laser beam with a bandwidth of 60 nm. Then, the beam was split into two parts. One was reflected by a Bragg filter (N013-14-A2, OptiGrate), producing near-infrared (805 nm) light with a bandwidth of 0.5 nm. The remaining light was directed through an optical parametric amplifier (OPA) and difference frequency generation system (TOPAS-C/DFG) to generate infrared light with a tunable central frequency ranging from 800 to 3800 cm⁻¹ and a bandwidth of 300 ~ 500 cm⁻¹. Finally, the near-infrared light passed through a half-wave plate, polarizer, and lens before hit the sample surface at a 45° incident angle, with a spot diameter of approximately 500 μm at the sample position. The infrared light was focused by an off-axis parabolic mirror and incident on the sample surface at 57°, with a spot diameter of 410 μm at the sample position. The generated sum-frequency light was collected by a spectrometer (Acton SP300i, Princeton Instruments) and CCD (PyLoN 1340×100, Princeton Instruments) after passing through a filter. The raw SFG spectra were normalized against those from a gold thin film taken under identical conditions, as the optical dispersion of gold is negligible within the experimental IR frequency range. We note that the interfacial sensitivity mainly refers to changes in the SFG intensity, which scales with ${\left|{{\chi }}^{\left(2\right)}\right|}^{2}$ and probes the interfacial polar response; by contrast, the nearly unchanged phonon frequencies and linewidths serve as a control for lattice stability. The low-temperature environment was provided by a self-built helium-free cryogenic system used in our previous study^48^, reaching a minimum temperature of 30 K for samples. Since spectral profiles of the KTO (001), (110), and (111) surface phonons showed only minor temperature dependence (Supplementary Fig. 6), spectra discussed in the text were all taken at room temperature unless otherwise specified.

## Supplementary information

Supplementary Information

Transparent Peer Review file

## Data Availability

The source data generated in this study have been deposited in the Figshare database at https://doi.org/10.6084/m9.figshare.30343798.

## Code availability

No custom code was used in this study.
