# Supplementary material for: Orientational enhancement of surface phonon polarity in superconducting KTaO₃ interfaces
Source: Nat Commun. 2026 Aug 14;17:9764. doi: 10.1038/s41467-026-76343-4 (PMC13572391; doi:10.1038/s41467-026-76343-4)
Supplement: Supplementary file 1 [file 41467_2026_76343_MOESM1_ESM.pdf]

Supplementary Information for

Orientational Enhancement of Surface Phonon Polarity in Superconducting $KTaO_3$ Interfaces

Tongying Liu[1], Jiashi Li[1], Shiyu Zhang[1], Tao Zhou[1], Fanjin Lu[1], Xiaoyang Chen[1], Jia Liu[2], Long Cheng[2], Xinyi Liu[1], Wei Li[1], Yanwu Xie[3], Xiaofang Zhai[2], Rui Peng[1], and Wei-Tao Liu[1]*

* Corresponding author email: wtliu@fudan.edu.cn

This file includes:

Supplementary Notes 1–4

Supplementary Figures 1–12

Supplementary Tables 1–8

Supplementary References

Supplementary Information Text

Supplementary Note 1. SFG basics

For the system we studied, the total nonlinear susceptibility comprises the surface and the bulk contributions, yielding the SF intensity as [1]

$$I(\omega_3) = \frac{{\omega_3}^2 \left|\chi_{eff}^{(2)}\right|^2 I(\omega_1) I(\omega_2)}{8\varepsilon_0 c^3 \cos^2 \beta_3}$$

with $I(\omega_i)$ and β_i denoting the beam intensity at ω_i and the angle of beam with respect to the sample surface normal, respectively. Experimentally, the raw SFG intensity is normalized against a bare gold reference standard. Because gold exhibits no significant optical dispersion or prominent resonance peaks within our probed mid-infrared range, it serves as an ideal standard to characterize the broadband IR profile. This normalization procedure accurately compensates for variations in the incident laser power and optical alignment efficiency. By dividing the raw sample signal by the gold reference signal acquired under identical conditions, the resulting normalized intensity is proportional to the squared modulus of the effective second-order nonlinear susceptibility, $\left|\chi_{eff}^{(2)}\right|^2$. Changes in SFG intensity therefore reflect changes in the effective interfacial nonlinear response, whereas the phonon frequencies and linewidths provide independent measures of the stability of the underlying lattice structures.

Generally, we can fit spectra using complex Lorentzian functions to extract the susceptibility tensor elements:

$$I \propto \left|\overleftrightarrow{\chi}_{NR} + \overleftrightarrow{\chi}_{R}\right|^2 = \left|\overleftrightarrow{\chi}_{NR} + \sum_q \frac{\overleftrightarrow{A}_q}{\omega_{IR} - \omega_q + i\Gamma_q}\right|^2 \qquad \text{(S1)}$$

where $\overleftrightarrow{\chi}_{NR}$ is the non-resonant background, and $\overleftrightarrow{\chi}_{R}$ is the resonant contribution, with $\overleftrightarrow{A}_q$,

ω_q, and Γ_q being the amplitude, frequency, and damping coefficient of the qth resonance mode, respectively. The resonant part, i.e., effective nonlinear susceptibility is $\chi^{(2)}_{eff} = \chi^{(2)}_{eff,S} + \chi^{(2)}_{eff,B}$ with $\chi^{(2)}_{eff,S}$ and $\chi^{(2)}_{eff,B}$ referring to the surface and bulk contributions to the effective nonlinear susceptibility, respectively. For the KTO-based heterojunction interface, assuming the built-in polar field exhibits an exponentially decaying profile $\mathbf{E} = E_Z e^{-z/\xi}\mathbf{z}$ (ξ is the thickness that field extends), the effective nonlinear susceptibilities are [1]:

$$\chi^{(2)}_{eff,S} = \left[\overleftrightarrow{L}_3 \cdot \vec{e}_3\right] \cdot \overleftrightarrow{\chi}^{(2)}_{\mathrm{S}} : \left[\overleftrightarrow{L}_1 \cdot \vec{e}_1\right]\left[\overleftrightarrow{L}_2 \cdot \vec{e}_2\right]$$

$$\chi^{(2)}_{eff,B} = \left[\overleftrightarrow{L}_3 \cdot \vec{e}_3\right] \cdot \left[\overleftrightarrow{\chi}^{(3)}_{\mathrm{B}} \frac{(E_Z \xi\ )}{1 - i\Delta k \xi}\right] : \left[\overleftrightarrow{L}_1 \cdot \vec{e}_1\right]\left[\overleftrightarrow{L}_2 \cdot \vec{e}_2\right] \tag{S2}$$

where $\overleftrightarrow{\chi}^{(2)}_S$ and $\overleftrightarrow{\chi}^{(3)}_B$ are the second and third order nonlinear susceptibility tensors of the surface and bulk, $\vec{e}_i$ and $\overleftrightarrow{L}_i$ are the unit polarization vector and corresponding transmission Fresnel coefficient (see in Supplementary Table 6 for our systems), respectively. The wavevector mismatch Δk is the reciprocal of the coherence length l_c, which for our setup is 30 nm [2]. The ARPES measurements reveal that the conductive layer thickness is 5.5 nm [3], resulting in $E_Z\xi/(1 - i\Delta k\xi) \approx E_Z\xi$.

Supplementary Note 2. Susceptibility tensor of KTO heterostructures

Supplementary Figure 10 describes a detailed flowchart of the theoretical treatment of experimental data and fitting procedure. Experimentally (purple boxes), we performed SFG anisotropy measurements to obtain the azimuthal angle (φ) dependent SFG spectra, then fit all spectra to derive both non-resonant and resonant parts of $\chi^{(2)}$. The fitted results are tabulated in Supplementary Tables 1, 2 and 8. The uncertainties represent standard errors calculated directly from the variance-covariance matrix upon convergence of the Levenberg-Marquardt fitting algorithm. The non-resonant background was found to be generally much weaker than the resonant signal, with $\chi_{NR}^{(2)}/\chi_{R}^{(2)}$ ~ 0.2-0.3 at the LO_4-derived phonon peak.

For each polarization combination, multiple tensor elements may contribute. To extract individual tensor elements, on the theoretical side (blue boxes), we first identified the independent tensor elements of each crystal plane via projecting from the bulk symmetry group. Then, the φ-dependence of each polarization combination, contributed from various $\overleftrightarrow{\chi}^{(2)}$ tensor elements, can be derived via a coordinate transformation from the crystal frame to lab frame. Combined, we can solve for individual susceptibility tensor elements, and by comparing them in different cases, we eventually get the enhancement factor. Below we provide details of the tensor elements analysis.

Symmetry analysis

For the $Pm\overline{3}m$ space group (O_h point group) that KTO belongs to, the third-order susceptibility has 21 non-zero tensor elements $\chi^{(3)}_{\alpha\beta\mu\nu}$ in crystal coordinate ($\alpha\beta\mu\nu \in \{a = [100], b = [010], c = [001]\}$), of which only four are independent, denoted as χ_1, χ_2, χ_3,

χ_4.

$$aaaa\left(=\chi^{(3)}_{aaaa}\right) = bbbb = cccc = \chi_1,$$

$$aabb = aacc = bbaa = bbcc = ccaa = ccbb = \chi_2,$$

$$abab = acac = baba = bcbc = caca = cbcb = \chi_3,$$

$$abba = acca = baab = bccb = caac = cbbc = \chi_4.$$

We can use 3×3×3×3 matrix ($\chi^{(3)}_{\alpha\beta\mu\nu}$ in $\alpha \times \beta \times \mu \times \nu$ position) to represent the susceptibility for clarity,

$$\overleftrightarrow{\chi}^{(3)}_{\alpha\beta\mu\nu} = \begin{pmatrix} \begin{pmatrix} \chi_1 & 0 & 0 \\ 0 & \chi_2 & 0 \\ 0 & 0 & \chi_2 \end{pmatrix} & \begin{pmatrix} 0 & \chi_3 & 0 \\ \chi_4 & 0 & 0 \\ 0 & 0 & 0 \end{pmatrix} & \begin{pmatrix} 0 & 0 & \chi_3 \\ 0 & 0 & 0 \\ \chi_4 & 0 & 0 \end{pmatrix} \\ \begin{pmatrix} 0 & \chi_4 & 0 \\ \chi_3 & 0 & 0 \\ 0 & 0 & 0 \end{pmatrix} & \begin{pmatrix} \chi_2 & 0 & 0 \\ 0 & \chi_1 & 0 \\ 0 & 0 & \chi_2 \end{pmatrix} & \begin{pmatrix} 0 & 0 & 0 \\ 0 & 0 & \chi_3 \\ 0 & \chi_4 & 0 \end{pmatrix} \\ \begin{pmatrix} 0 & 0 & \chi_4 \\ 0 & 0 & 0 \\ \chi_3 & 0 & 0 \end{pmatrix} & \begin{pmatrix} 0 & 0 & 0 \\ 0 & 0 & \chi_4 \\ 0 & \chi_3 & 0 \end{pmatrix} & \begin{pmatrix} \chi_2 & 0 & 0 \\ 0 & \chi_2 & 0 \\ 0 & 0 & \chi_1 \end{pmatrix} \end{pmatrix}.$$

The transformation from the crystal coordinates (α, β, μ, $\nu \in \{a, b, c\}$) to the lab coordinates ($i, j, k, l \in \{x, y, z\}$) follows:

$$\chi^{(3)}_{ijkl} = T_{i\alpha}T_{j\beta}T_{k\mu}T_{l\nu}\chi^{(3)}_{\alpha\beta\mu\nu}$$

where $T^{\top}T = I$, $T^{-1} = T$, $(T^{-1})_{\beta j} = T_{j\beta}$.

The subindices of $\chi^{(3)}_{ijkl}$ from left to right represent the direction of SFG, NIR, IR and the field term, respectively. Given the close proximity of the SFG and NIR wavelengths, we further have $\chi_3 \approx \chi_4$. The coordinates used for the three crystal facets are shown in Supplementary Figure 7, with z along the surface normal and zx being the beam incident plane.

(001)

For (001) surface, the crystal coordinate is defined with x'-axis along the a-axis and z' along the c-axis in sample coordinates. The transformation matrix from the crystal to the lab is

$$T_{(001)} = \begin{pmatrix} \cos\varphi & -\sin\varphi & 0 \\ \sin\varphi & \cos\varphi & 0 \\ 0 & 0 & 1 \end{pmatrix} \begin{pmatrix} 1 & 0 & 0 \\ 0 & 1 & 0 \\ 0 & 0 & 1 \end{pmatrix}.$$

Since the field is along z direction, only $l = z$ terms are permitted:

$$\chi^{(3)}_{ijkz} = \begin{pmatrix} \begin{pmatrix} 0 \\ 0 \\ \chi_2 \end{pmatrix} & \begin{pmatrix} 0 \\ 0 \\ 0 \end{pmatrix} & \begin{pmatrix} \chi_3 \\ 0 \\ 0 \end{pmatrix} \\ \begin{pmatrix} 0 \\ 0 \\ 0 \end{pmatrix} & \begin{pmatrix} 0 \\ 0 \\ \chi_2 \end{pmatrix} & \begin{pmatrix} 0 \\ \chi_3 \\ 0 \end{pmatrix} \\ \begin{pmatrix} \chi_3 \\ 0 \\ 0 \end{pmatrix} & \begin{pmatrix} 0 \\ \chi_3 \\ 0 \end{pmatrix} & \begin{pmatrix} 0 \\ 0 \\ \chi_1 \end{pmatrix} \end{pmatrix}.$$

Substitute it into Eq. (2) and for the SSP and PPP polarization combinations used in this study, the effective susceptibility can be expressed as

$$\begin{aligned} \chi^{(2)}_{eff}(\mathrm{SSP}) &= \mathcal{L}_{yyz}\chi_2 \\ \chi^{(2)}_{eff}(\mathrm{PPP}) &= \mathcal{L}_{zzz}\chi_1 - \mathcal{L}_{xxz}\chi_2 + (\mathcal{L}_{zxx} - \mathcal{L}_{xzx})\chi_3 \end{aligned} \qquad \text{(S3)}$$

where $\mathcal{L}_{ijk}$ are the dimensionless coefficients including Fresnel coefficients after projection to the polarization vector, for example, $\mathcal{L}_{yyz}$ represents $\sin\beta_2 L_{yy}(\omega_1)\, L_{yy}(\omega_2) L_{zz}(\omega_3)$. All coefficients can be obtained from Kramers-Kronig relations [4,5] and listed in Supplementary Table 6. Besides, the term $\mathcal{L}_{zxx} - \mathcal{L}_{xzx} = 0.002$ is three orders of magnitude smaller than the other terms and can therefore be neglected.

Evidently, Eq. (S3) implies that for the (001) plane, the SF intensity is azimuthally invariant, i.e., isotropic, as indeed depicted in Fig. 1e and 2e. By fitting the patterns (see

Supplementary Table 2), we can deduce the susceptibility components as follows:

$$\chi_1 = (C_{iso}^{PPP} + \mathcal{L}_{xxz} C_{iso}^{SSP} / \mathcal{L}_{yyz}) \mathcal{L}_{zzz},$$

$$\chi_2 = C_{iso}^{SSP} / \mathcal{L}_{yyz}.$$

where the superscript denotes the polarization and the subscript represents the isotropic term coefficient.

(110)

For (110) surface, the coordinates are defined with x'-axis along c-axis and z'-axis along the [110] direction. The transformation matrix from the crystal to the lab and non-vanishing terms are:

$$T_{(110)} = \begin{pmatrix} \cos\varphi & -\sin\varphi & 0 \\ \sin\varphi & \cos\varphi & 0 \\ 0 & 0 & 1 \end{pmatrix} \begin{pmatrix} 0 & 0 & 1 \\ 1/\sqrt{2} & -1/\sqrt{2} & 0 \\ 1/\sqrt{2} & 1/\sqrt{2} & 0 \end{pmatrix}.$$

$$\overleftrightarrow{\chi}_{ijkl}^{(3)} = \begin{pmatrix} \begin{pmatrix} 0 \\ 0 \\ \chi_2 - \frac{\chi'}{2}\sin^2\varphi \end{pmatrix} & \begin{pmatrix} 0 \\ 0 \\ \frac{\chi'}{4}\sin 2\varphi \end{pmatrix} & \begin{pmatrix} \chi_3 - \frac{\chi'}{2}\sin^2\varphi \\ \frac{\chi'}{4}\sin 2\varphi \\ 0 \end{pmatrix} \\ \begin{pmatrix} 0 \\ 0 \\ \frac{\chi'}{4}\sin 2\varphi \end{pmatrix} & \begin{pmatrix} 0 \\ 0 \\ \chi_2 - \frac{\chi'}{2}\cos^2\varphi \end{pmatrix} & \begin{pmatrix} \frac{\chi'}{4}\sin 2\varphi \\ \chi_3 - \frac{\chi'}{2}\cos^2\varphi \\ 0 \end{pmatrix} \\ \begin{pmatrix} \chi_3 - \frac{\chi'}{2}\sin^2\varphi \\ \frac{\chi'}{4}\sin 2\varphi \\ 0 \end{pmatrix} & \begin{pmatrix} \frac{\chi'}{4}\sin 2\varphi \\ \chi_3 - \frac{\chi'}{2}\cos^2\varphi \\ 0 \end{pmatrix} & \begin{pmatrix} 0 \\ 0 \\ \frac{\chi'}{2} + \chi_1 \end{pmatrix} \end{pmatrix}.$$

Note that we define the terms $\chi' \equiv -\chi_1 + \chi_2 + 2\chi_3$ for clarity. Substitute it into Eq. (S2) and for the SSP and PPP polarization combinations used in this study, the effective susceptibility can be expressed as

$$\chi_{eff}^{(2)}(\mathrm{SSP}) = C_{ani}^{SSP} \cos 2\varphi + C_{iso}^{SSP}$$

$$\chi_{eff}^{(2)}(\mathrm{PPP}) = C_{ani}^{PPP}\cos 2\varphi + C_{iso}^{PPP} \tag{S4}$$

where the C_{ani}^{PPP} and C_{ani}^{SSP} are the anisotropic coefficients respectively. Here are the values:

$$C_{ani}^{SSP} = \mathcal{L}_{yyz}(\chi_1 - \chi_2 - 2\chi_3)/4,$$

$$C_{iso}^{SSP} = \mathcal{L}_{yyz}(\chi_1 + 3\chi_2 - 2\chi_3)/4,$$

$$C_{ani}^{PPP} = (\mathcal{L}_{xxz} + \mathcal{L}_{xzx} - \mathcal{L}_{zxx})(\chi_1 - \chi_2 - 2\chi_3)/4,$$

$$C_{iso}^{PPP} = [(-\mathcal{L}_{xxz} - \mathcal{L}_{xzx} + \mathcal{L}_{zxx} + 2\mathcal{L}_{zzz})\chi_1$$

$$+(-3\mathcal{L}_{xxz} + \mathcal{L}_{xzx} - \mathcal{L}_{zxx} + 2\mathcal{L}_{zzz})\chi_2$$

$$+2(\mathcal{L}_{xxz} - \mathcal{L}_{xzx} + \mathcal{L}_{zxx} + 2\mathcal{L}_{zzz})\chi_3]/4.$$

Obviously, the $\cos 2\varphi$ terms suggest the anisotropic pattern in the spectra, consistent with C_{2v} symmetry of the (110) plane.

(111)

For (111) surface, the coordinates are defined with x'-axis along the [1-10] direction z'-axis along the [111] direction. The transformation matrix from the crystal to the lab and non-vanishing terms are:

$$T_{(111)} = \begin{pmatrix} \cos\varphi & -\sin\varphi & 0 \\ \sin\varphi & \cos\varphi & 0 \\ 0 & 0 & 1 \end{pmatrix}\begin{pmatrix} 1/\sqrt{2} & -1/\sqrt{2} & 0 \\ 1/\sqrt{6} & 1/\sqrt{6} & -2/\sqrt{6} \\ 1/\sqrt{3} & 1/\sqrt{3} & 1/\sqrt{3} \end{pmatrix}.$$

$$\overleftrightarrow{\chi}^{(3)}_{ijkl} = \begin{pmatrix} \begin{pmatrix} \frac{\sqrt{2}}{6}\chi' \sin 3\varphi \\ \frac{-\sqrt{2}}{6}\chi' \cos\varphi \\ \chi_2 - \chi'/3 \end{pmatrix} & \begin{pmatrix} \frac{-\sqrt{2}}{6}\chi' \cos 3\varphi \\ \frac{-\sqrt{2}}{6}\chi' \sin 3\varphi \\ 0 \end{pmatrix} & \begin{pmatrix} \chi_3 - \chi'/3 \\ 0 \\ 0 \end{pmatrix} \\ \begin{pmatrix} \frac{-\sqrt{2}}{6}\chi' \cos 3\varphi \\ \frac{-\sqrt{2}}{6}\chi' \sin 3\varphi \\ 0 \end{pmatrix} & \begin{pmatrix} \frac{-\sqrt{2}}{6}\chi' \sin 3\varphi \\ \frac{\sqrt{2}}{6}\chi' \cos 3\varphi \\ \chi_2 - \chi'/3 \end{pmatrix} & \begin{pmatrix} 0 \\ \chi_3 - \chi'/3 \\ 0 \end{pmatrix} \\ \begin{pmatrix} \chi_3 - \chi'/3 \\ 0 \\ 0 \end{pmatrix} & \begin{pmatrix} 0 \\ \chi_3 - \chi'/3 \\ 0 \end{pmatrix} & \begin{pmatrix} 0 \\ 0 \\ \chi_1 - \chi'/3 \end{pmatrix} \end{pmatrix}.$$

Substitute it into Eq. (S2) and the effective susceptibility can be expressed as

$$\chi^{(2)}_{eff}(\mathrm{SSP}) = C^{SSP}_{ani}\sin 3\varphi + C^{SSP}_{iso}$$

$$\chi^{(2)}_{eff}(\mathrm{PPP}) = C^{PPP}_{ani}\sin 3\varphi + C^{PPP}_{iso} \qquad \text{(S5)}$$

with the coefficients

$$C^{SSP}_{ani} = \sqrt{2}\mathcal{L}_{yyx}(\chi_1 - \chi_2 - 2\chi_3)/6,$$

$$C^{SSP}_{iso} = \mathcal{L}_{yyz}(\chi_1 + 2\chi_2 - 2\chi_3)/3,$$

$$C^{PPP}_{ani} = \sqrt{2}\mathcal{L}_{xxx}(\chi_1 - \chi_2 - 2\chi_3)/6,$$

$$C^{PPP}_{iso} = [(-\mathcal{L}_{xxz} - \mathcal{L}_{xzx} + \mathcal{L}_{zxx} + \mathcal{L}_{zzz})\chi_1$$

$$+(-2\mathcal{L}_{xxz} + \mathcal{L}_{xzx} - \mathcal{L}_{zxx} + 2\mathcal{L}_{zzz})\chi_2$$

$$+2(\mathcal{L}_{xxz} - \mathcal{L}_{xzx} + \mathcal{L}_{zxx} + 4\mathcal{L}_{zzz})\chi_3]/3.$$

Now we have obtained expressions for the effective susceptibility for all three orientations under SSP and PPP polarizations. With the coefficients fitted from experimental patterns in Supplementary Table 2, we can solve the system of equations (S3)-(S5) for the value of $\chi^{(2)}_{B,1}$, $\chi^{(2)}_{B,2}$ and $\chi^{(2)}_{B,3}$, as tabled in Supplementary Table 3. The same analysis can be applied to the KTO substrate, which is primarily a surface signal, to obtain

the value of $\chi_{S,i}^{(2)}$, as listed in Supplementary Table 4. We note that the equations for C_{ani}^{SSP} and C_{ani}^{PPP} are equivalent. Taking either value yields comparable field enhancement factor, which validates the reliability of our findings. Since the lattice itself is not expected to change drastically with varying orientations, the discrepancies between LAO/KTO and substrates are mainly caused by the change in polar fields after the growth of the LAO. The polarity enhancement factors are tabulated in Supplementary Table 5 and plotted in Fig. 3a of the main text. Although the reverse derivation process is highly complex, the results exhibit consistency across different crystal planes and align with the superconducting trend: (111) > (110) >> (001). While the experiments were conducted at room temperature, the interfacial properties probed by sum-frequency generation are intrinsic. The extracted polarity-enhancement factors exhibit the same orientation dependence as T_c. This empirical correspondence suggests that orientation-dependent interfacial symmetry breaking may influence the electronic environment through the combined effects of SOC and EPC.

Supplementary Note 3. Dynamical polarization

The lab coordinate is chosen with the Ta atoms as the center. Assuming the charge q_i is located at position $\vec{r_i}$, the geometric center position is then determined to be $R = \sum q_i\vec{r}_i \,/\, \sum q_i$. After calculating the geometric centers of the positive R_+ and negative R_- charges, the dipole moment can be obtained as $\vec{p} = (q_+ + q_-)(R_+ - R_-)/2$. We calculated both the nominal and the dynamical Born effective charges (BECs). In the nominal case, the K, Ta, and O ions have charges of +1, +5, and -2, respectively. It is an ill-defined quantity because artificially dividing the ground-state electronic density to different atoms is ambiguous [6]. The dynamical Born effective charge describes the effective charge of an ion in a crystal lattice when it is displaced from its equilibrium position. It is related to the change of polarization created by an atomic displacement. Typical values of born effective charges for K, Ta and O ions are 1.14, 8.86, and -6.64 e, respectively [7,8].

The surfaces are depicted in Supplementary Figure 9. The Ta ion is at the origin with a charge of q_{Ta}. Considering the (001) configuration, the coordinates of the four K ions are (±a/2, ±a/2, -a/2) with the charge q_{K} in one primitive cell, where $a = 3.988$ Å is the lattice constant [9]. There are five oxygen ions with a negative charge of q_{O} located at (±a/2, 0, 0), (0, ±a/2, 0) and (0, 0, -a/2), respectively. The positive and negative centers are

$$R_+ = (\sum q_{\mathrm{K}}\vec{r}_{\mathrm{K}} + q_{\mathrm{Ta}}\vec{r}_{\mathrm{Ta}})/(\sum q_{\mathrm{K}} + q_{\mathrm{Ta}})$$

$$= (0, 0, -2aq_{\mathrm{K}}/(\sum q_{\mathrm{K}} + q_{\mathrm{Ta}})),$$

$$R_- = \sum q_{\mathrm{O}}\vec{r}_{\mathrm{O}} \,/\, \sum q_{\mathrm{O}} \; = (0, 0, -aq_{\mathrm{O}}/2\sum q_{\mathrm{O}}).$$

For the nominal case, we have $q_{\mathrm{Ta}} = 5$ e $q_{\mathrm{K}} = 0.125$ e and $q_{\mathrm{O}} = 1$ e. Note that in one primitive cell, the K ions on the corners count as one-eighth and the O ions on the faces

count as one-half. Then, the dipole yields $p_{z,nom}$ = 1.142 e·Å. For the BECs case, we have q_{Ta} = 8.86 e, q_K = 0.1425 e and q_O = 3.32 e. The dipole is given by $p_{z,BECs}$ = 3.622 e·Å.

With respect to (110) surface, the positive and negative centers are

$$R_+ = (0, 0, -\sqrt{2}aq_K / \sum q_K + q_{Ta}),$$

$$R_- = (0, 0, -aq_O / \sum q_O).$$

Similarly, we have $p_{z,nom}$ = 4.263 e·Å and $p_{z,BECs}$ = 10.512 e·Å.

Regarding the (111) surface, the positive and negative centers are

$$R_+ = (0, 0, -\sqrt{3}aq_K / \sum q_K + q_{Ta}),$$

$$R_- = (0, 0, -\sqrt{3}aq_O / 2\sum q_O).$$

Likewise, we have $p_{z,nom}$ = 4.226 e·Å and $p_{z,BECs}$ = 10.149 e·Å.

The values of dipole moment in z direction p_z are tabulated in Supplementary Table 7. In the nominal case, the dipole moments of (110) and (111) surfaces are 3.7 times larger than the (001) surface. Under BECs conditions, the dipole moments ofthe (110) and (111) surfaces are 2.9 and 2.8 times larger than the (001) surface, respectively. In both cases, the polarization of the (110) and (111) surfaces is stronger than that of the (001) surface.

Supplementary Note 4. Optical Effects from the LAO Capping Layer

To confirm that the substantial enhancement in the measured SFG intensity of the LAO/KTO heterostructures cannot be explained by linear optical effects from the LAO overlayer, we evaluated both IR absorption and local Fresnel-field modulation introduced by the capping layer, we systematically evaluated both the IR absorption and the local Fresnel field modulation introduced by the capping layer. According to previous IR studies[10], LAO does not have resonance features between 90 and 120 meV (726 ~ 968 cm^{-1}). Consequently, anomalous dispersion or drastic absorption features are not expected in this energy regime.

To quantitatively assess the impact of the LAO film on the local electric field driving the SFG process at the KTO interface, we modeled the heterostructure using the transfer-matrix method, which accounts for both multi-beam interference and absorption within the thin film. We calculated the local IR Fresnel factors by comparing a two-layer reference system (Air/KTO), where the local IR optical field (E_{Bare}) depends on the refractive indices of air (n_1) and KTO ($\tilde{n}_3$), against a three-layer microcavity system (Air/LAO/KTO), where the local interfacial IR field E_{Film} is additionally modulated by the complex refractive index ($\tilde{n}_2$)and physical thickness (d) of the LAO film[11].

In the three-layer model, the local interfacial IR field depends on the transmission coefficients at both the air/LAO and LAO/KTO boundaries, coupled with a complex phase-thickness term $\beta = \frac{2\pi}{\lambda} d\sqrt{\tilde{n}_2^2 - n_1^2 \sin^2 \theta_{inc}}$. This term captures the phase accumulation and amplitude decay (absorption) of the IR beam as it undergoes multiple reflections within the LAO layer. Using established frequency-dependent optical constants for LAO[12]and KTO[13], we calculated the intensity ratio of the local driving fields, defined as

$|E_{\mathrm{Film}}|^2/|E_{\mathrm{Bare}}|^2$. For a 2 nm-thick LAO film, the phase thickness β is exceptionally small in the mid-IR regime. Even when incorporating the dispersive extinction coefficient of LAO, the maximum intrinsic absorption within the 2 nm layer is calculated to be only ~0.35%. Consequently, the calculated $|E_{\mathrm{Film}}|^2/|E_{\mathrm{Bare}}|^2$ ratio remains exceptionally close to unity (deviating by less than 1%) across the entire 90–120 meV frequency range. This demonstrates that the 2 nm LAO layer does not act as a significant optical cavity or an absorbing barrier. Hence optically, the influence of the LAO-capping layer is negligible to our measurements.

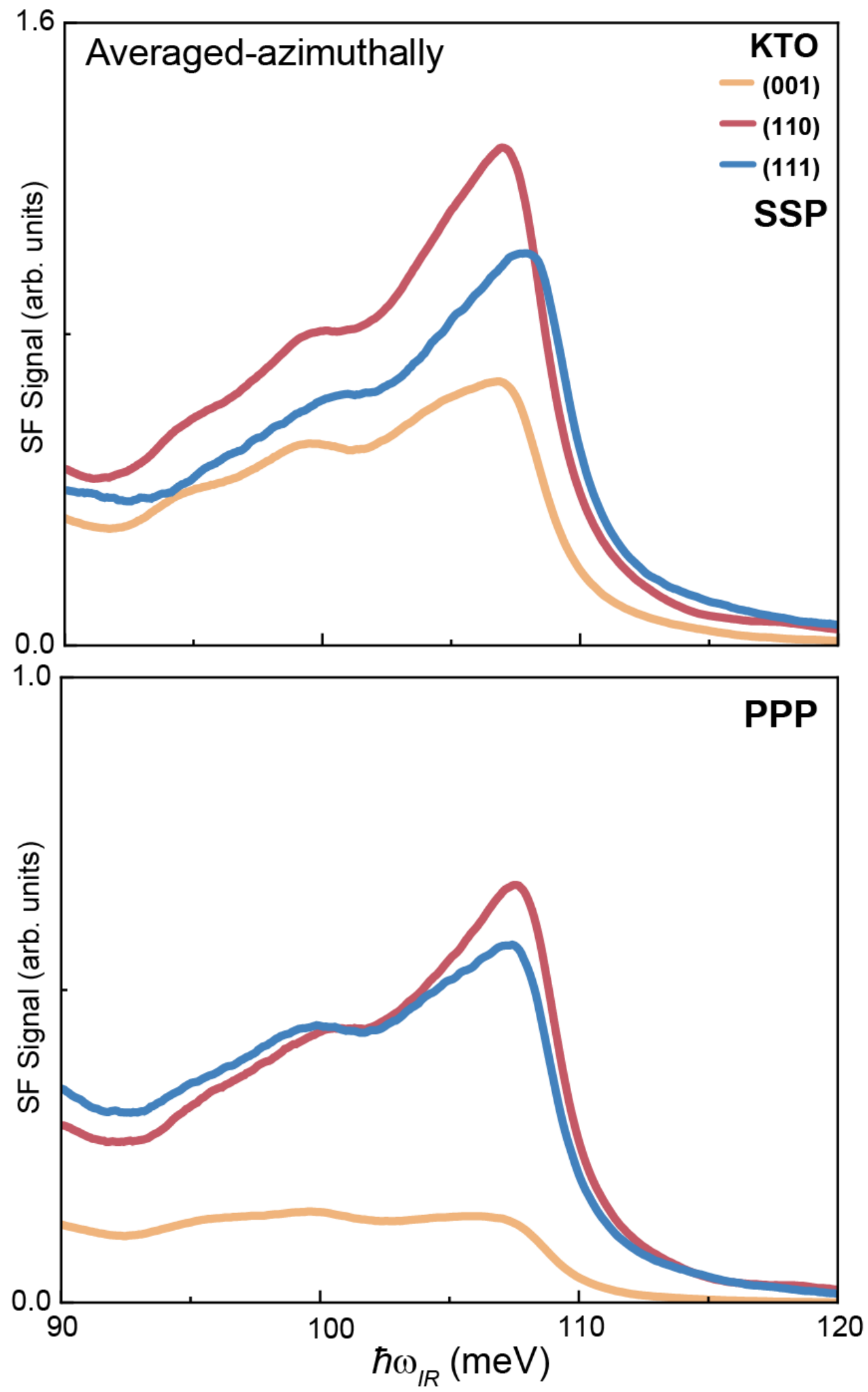

Supplementary Figure 1 The averaged-azimuthally spectra of KTO substrate with (001), (110) and (111) orientation under SSP and PPP combination. All normalized to the signals from a non-resonant gold thin film.

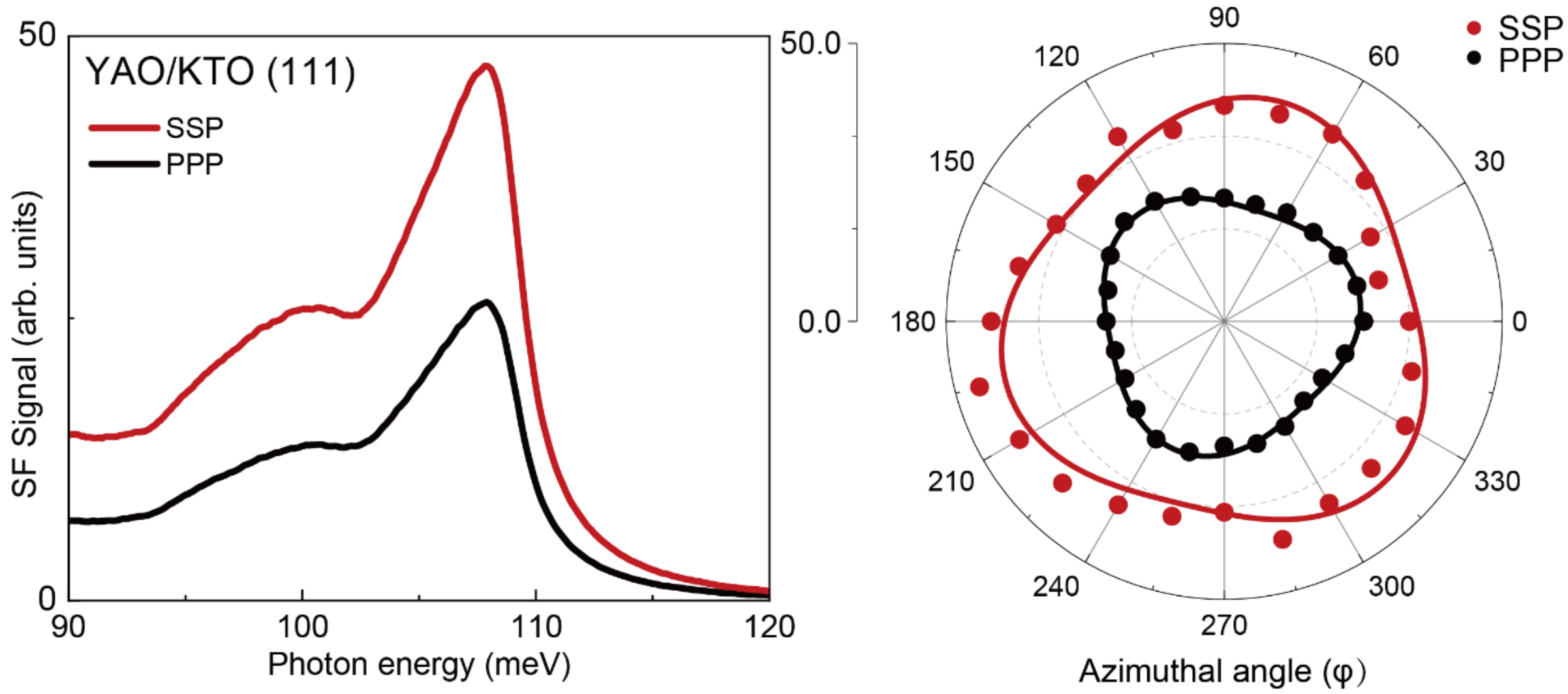

Supplementary Figure 2 The SF spectra of YAO/KTO (111) and the azimuthal patterns (see fitting parameters in Supplementary Table 2)

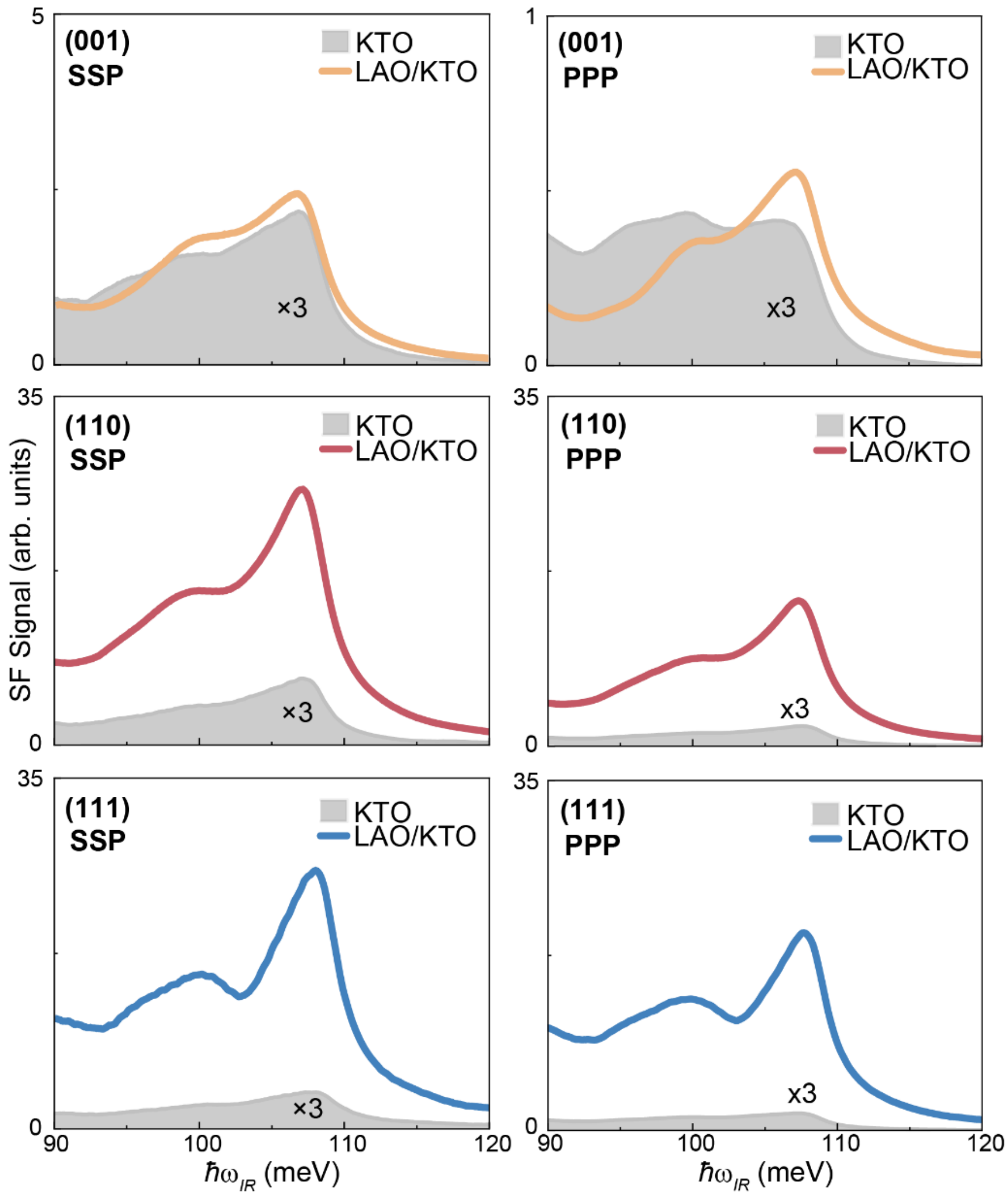

Supplementary Figure 3 The azimuthally-averaged SSP and PPP spectra from LAO/KTO with (001), (110) and (111) orientations (colored) and corresponding substrate (gray filled, amplified by 3 times).

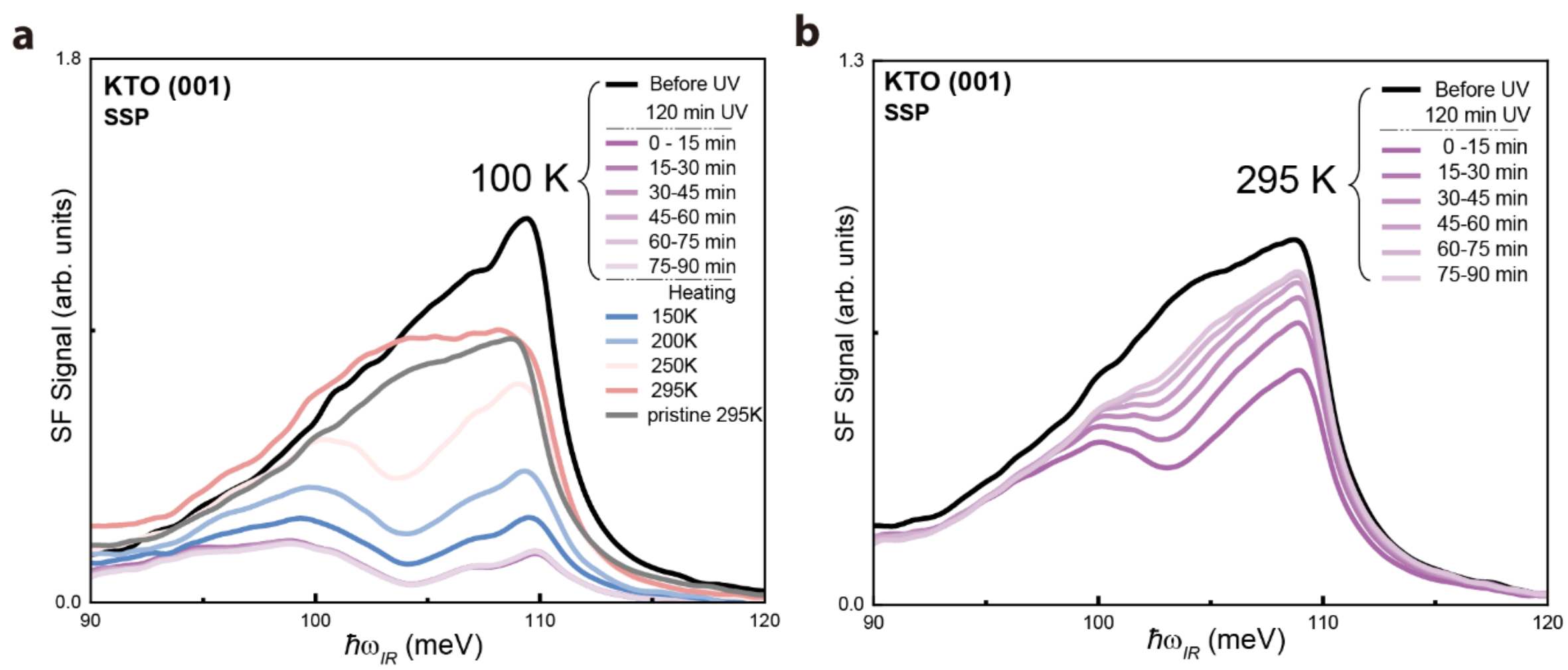

Supplementary Figure 4 a Spectra of KTO (001) upon UV irradiation at 100 K, and after UV irradiation at elevated temperature. **b** Spectra of KTO (001) upon UV irradiation before (black) and after 120 minutes of UV irradiation (purple) at 295 K.

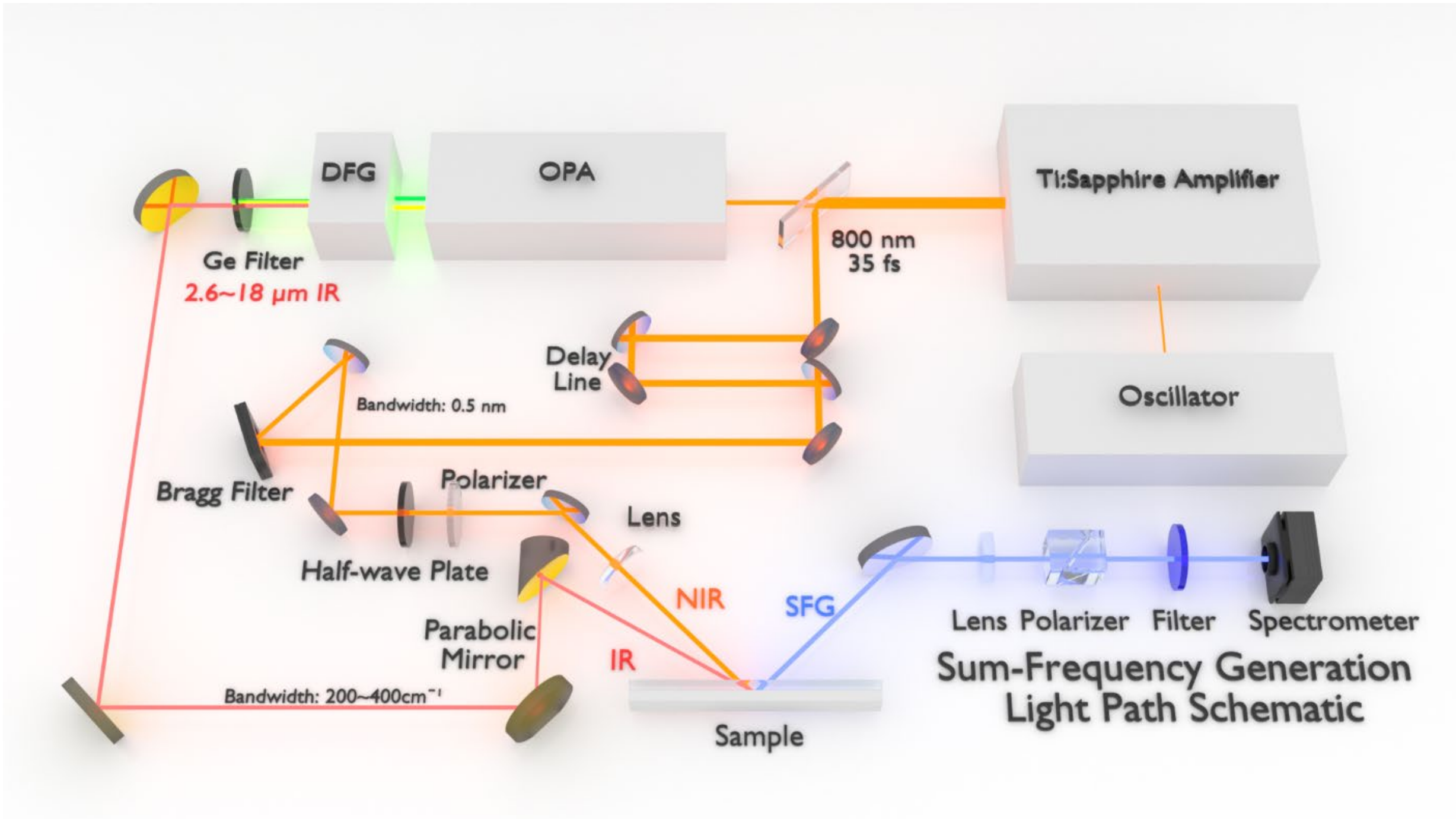

Supplementary Figure 5 The schematic of femtosecond laser system. OPA: optical parametric amplifier. DFG: Difference Frequency Generation.

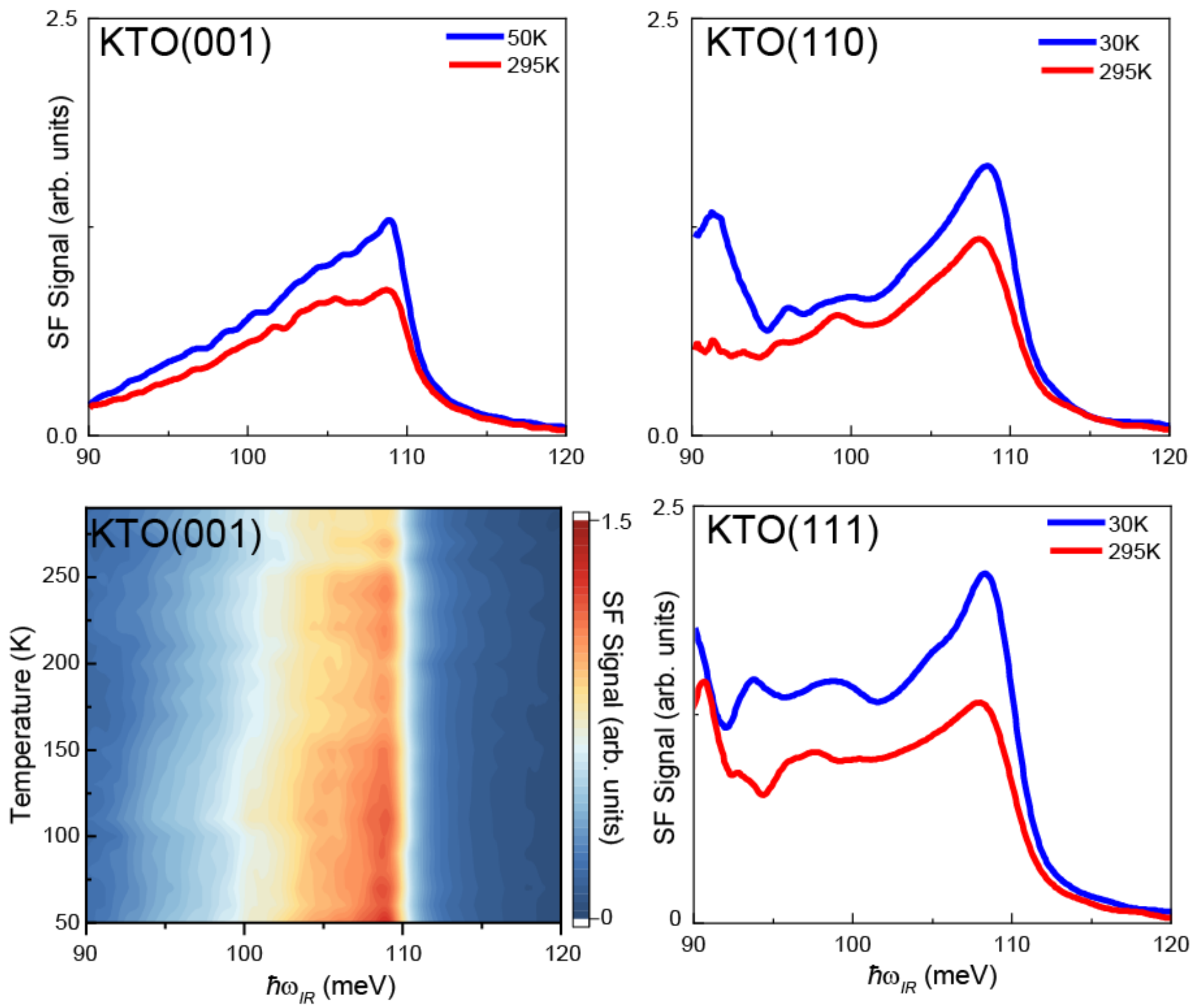

Supplementary Figure 6 The temperature dependence of KTO (001), (110) and (111) spectra.

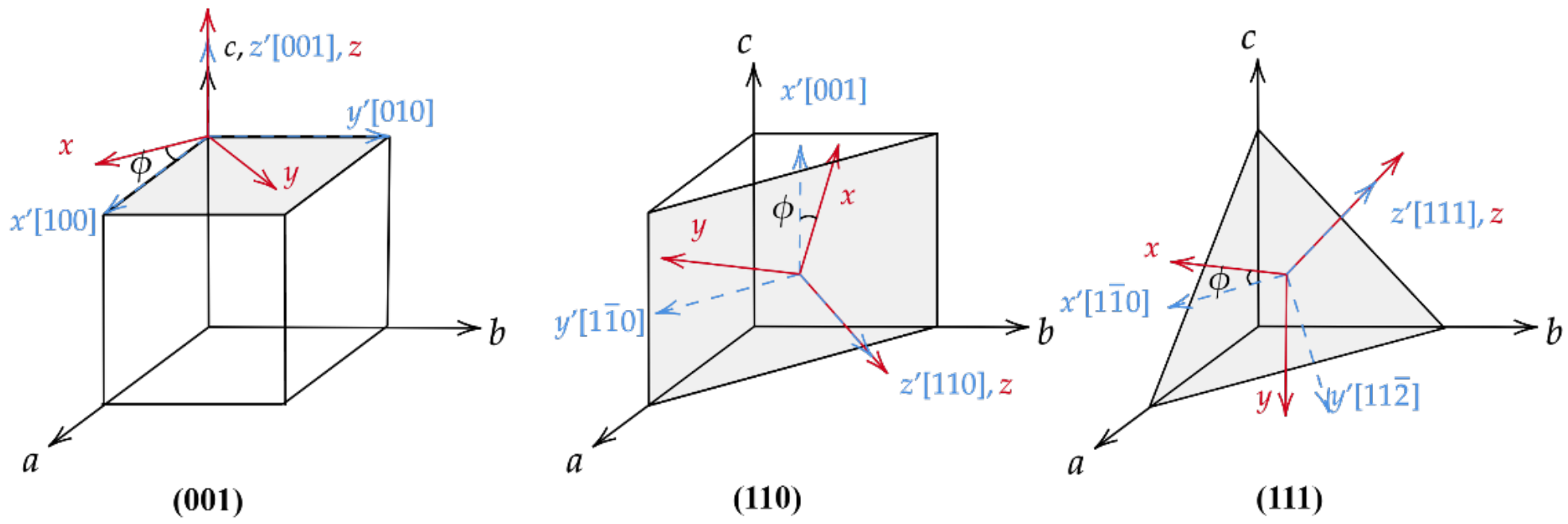

Supplementary Figure 7 The sketch of the crystal {*a*, *b*, *c*} (black) and lab {*x*, *y*, *z*} (red) coordinates. The blue one {*x'*, *y'*, *z'*} denotes initial $\varphi = 0°$ state.

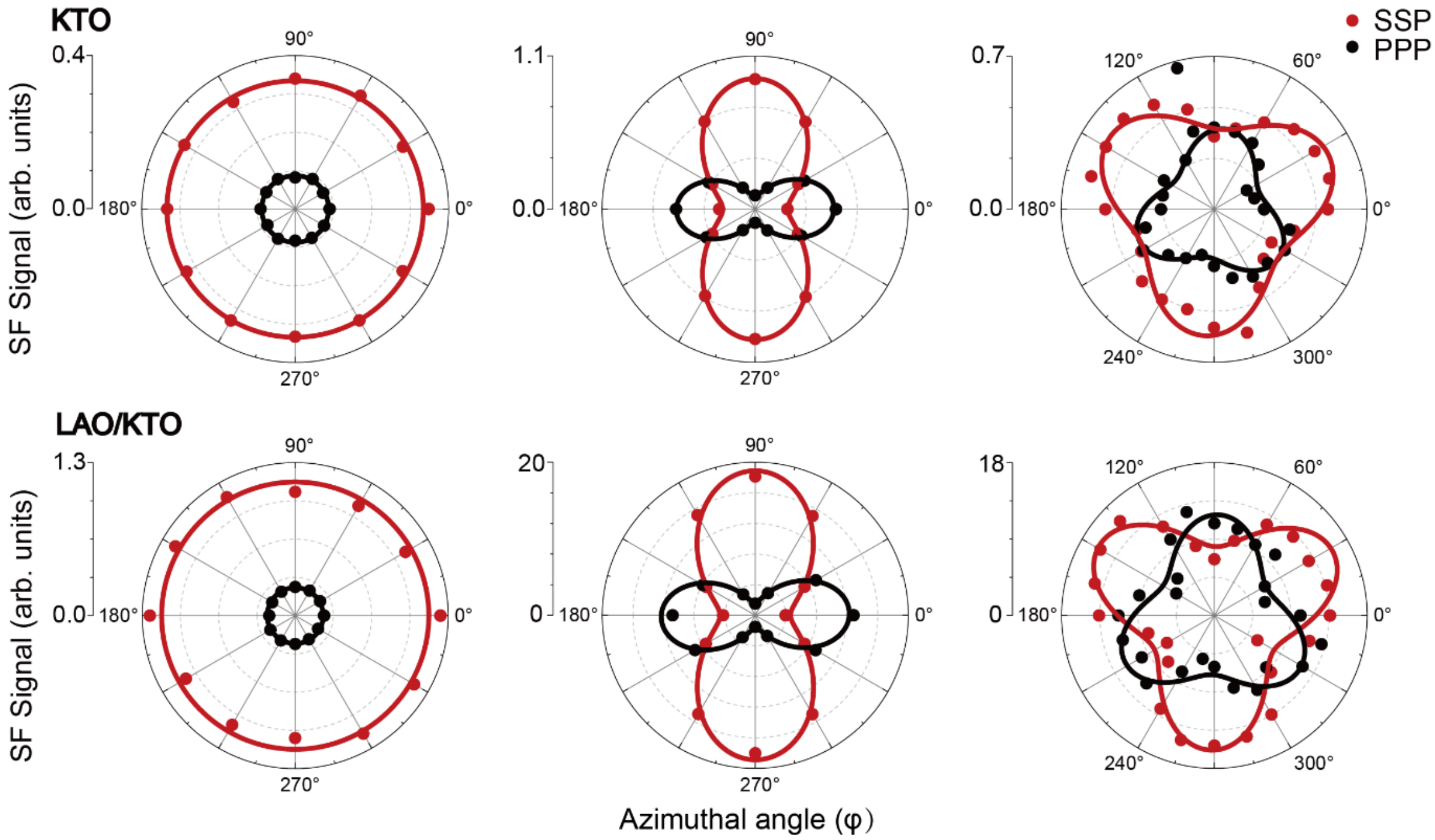

Supplementary Figure 8 Anisotropy patterns of bare KTO substrates and LAO/KTO samples with signals averaged within range 90~120 meV, showing the same trends of those averaged between 106~108 meV as in Figs. 1 and 2 of the main text. The fitting parameters were listed in Supplementary Table 2.

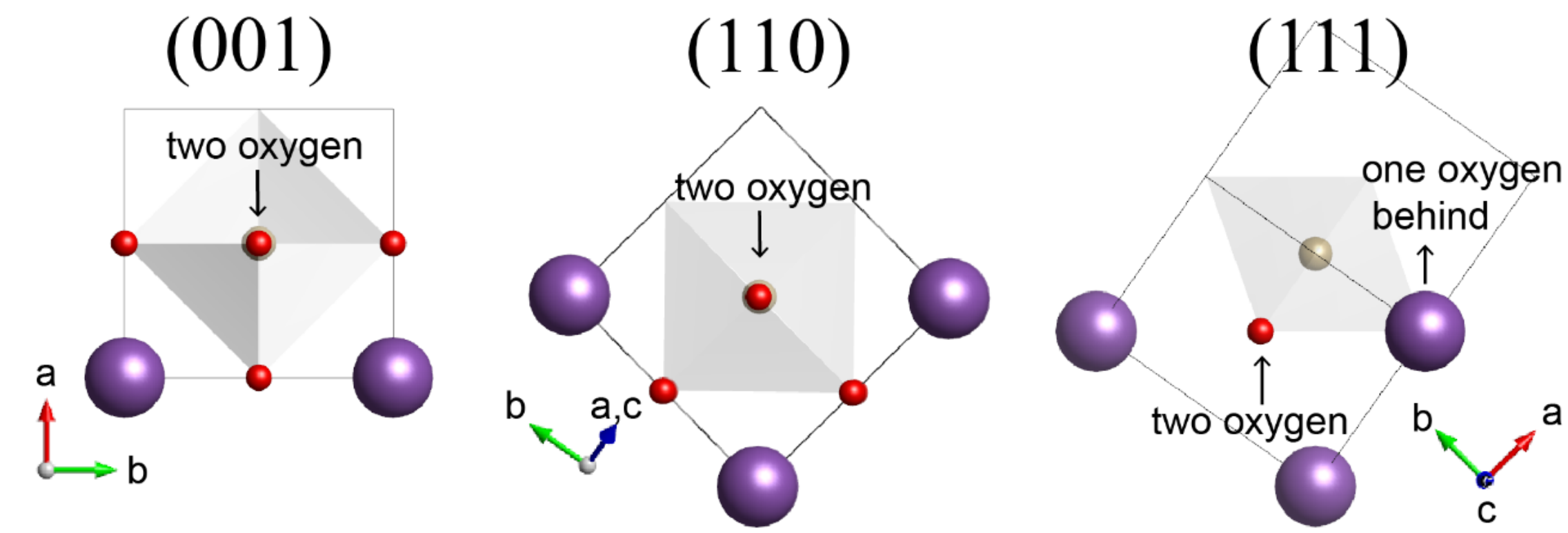

Supplementary Figure 9 The sketch of semi-infinite model for three surfaces to estimate dipole moments.

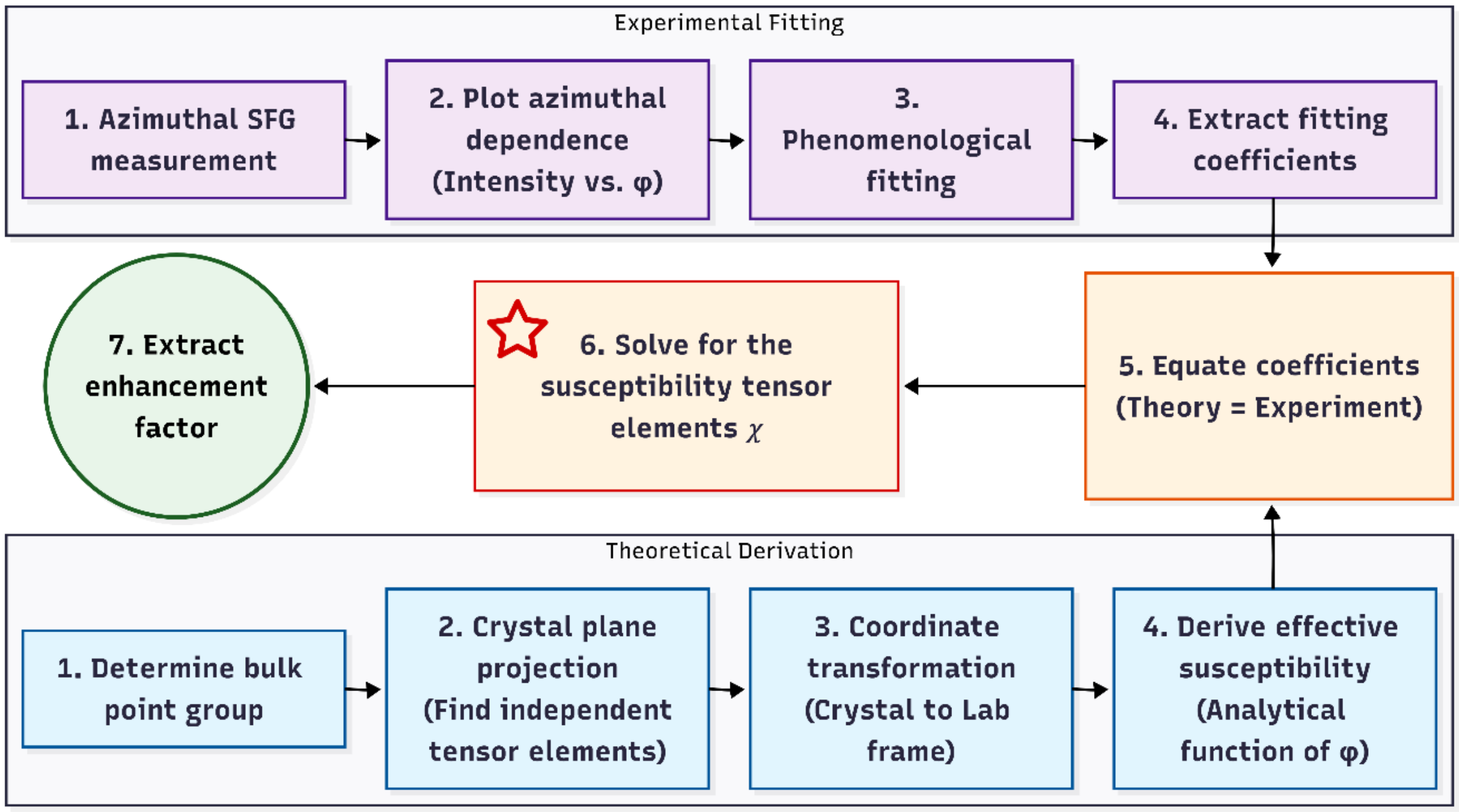

Supplementary Figure 10 Derivation Workflow of Nonlinear Susceptibility.

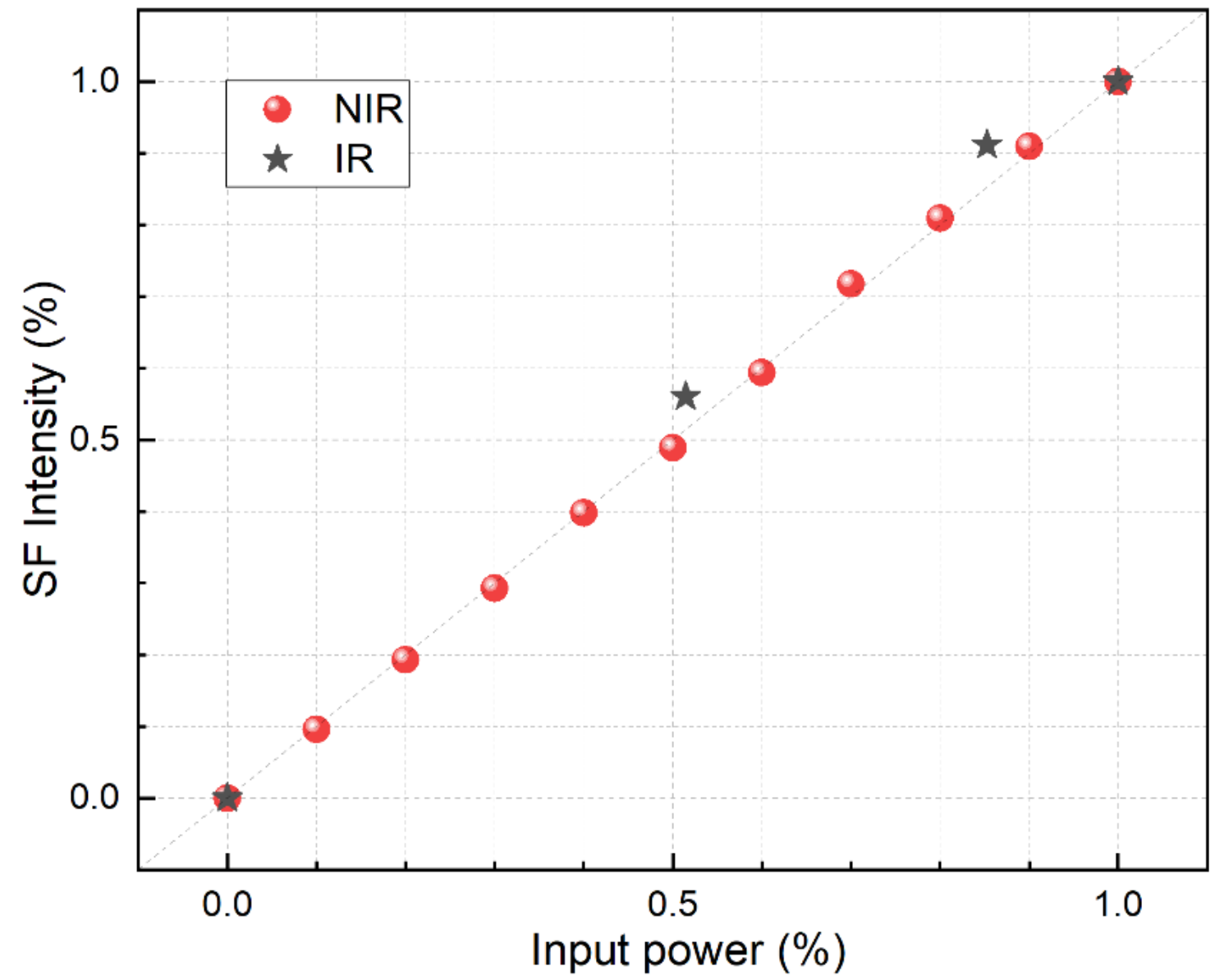

Supplementary Figure 11 Laser power dependence of the SFG signal. Both IR and NIR powers are normalized to corresponding maximum values.

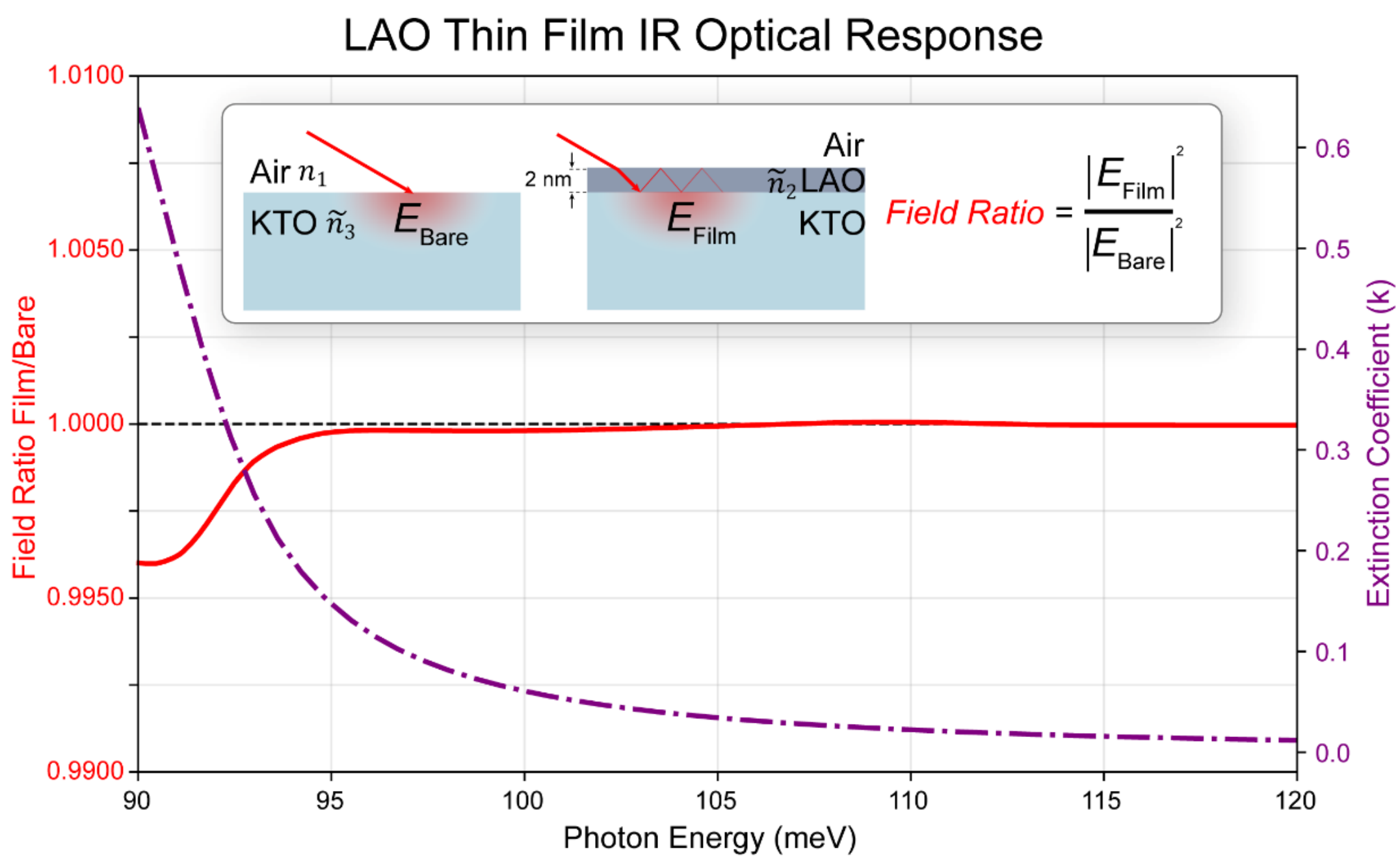

Supplementary Figure 12 IR optical response of LAO film.

Time (s)	ω_1 (meV)	A_1 (arb.units)	A_2 (arb.units)
10	109.42 ± 0.01	3.08 ± 0.02	0.51 ± 0.02
60	109.82 ± 0.02	2.44 ± 0.01	0.71 ± 0.02
120	110.01 ± 0.04	2.20 ± 0.01	0.73 ± 0.02
180	110.18 ± 0.03	2.01 ± 0.01	0.73 ± 0.02
300	110.42 ± 0.04	1.80 ± 0.02	0.72 ± 0.02
480	110.72 ± 0.06	1.59 ± 0.02	0.71 ± 0.02
600	110.82 ± 0.06	1.49 ± 0.02	0.75 ± 0.02
1200	111.47 ± 0.10	1.13 ± 0.02	0.79 ± 0.02
1800	111.59 ± 0.11	1.01 ± 0.03	0.87 ± 0.02
3600	111.87 ± 0.14	0.68 ± 0.04	0.90 ± 0.02
7200	111.78 ± 0.14	0.70 ± 0.04	0.89 ± 0.02

Supplementary Table 1 Fitting parameters of KTO (001) after UV illumination at 60 K. The global fitting was employed to ensure the reliability with $\mathrm{Re}(\chi_{NR}) = -0.23\pm0.01$, $\mathrm{Im}(\chi_{NR}) = -0.26\pm0.01$, $\Gamma_1 = 2.43\pm0.01$ meV, $\omega_2 = 101.76\pm0.02$ meV, $\Gamma_2 = 2.83\pm0.05$ meV.

Samples		Range	SSP			PPP		
			$C_{\text{ani}}^{\text{SSP}}$	$C_{\text{iso}}^{\text{SSP}}$	φ_0 (°)	$C_{\text{ani}}^{\text{PPP}}$	$C_{\text{iso}}^{\text{PPP}}$	φ_0 (°)
KTO	(001)	I	—	0.808 ± 0.005	—	—	0.362 ± 0.002	—
		II	—	0.578 ± 0.002	—	—	0.295 ± 0.001	—
	(110)	I	0.383 ± 0.005	1.076 ± 0.004	1.044 ± 0.761	0.347 ± 0.008	0.766 ± 0.006	-0.684 ± 1.1.268
		II	-0.243 ± 0.003	0.726 ± 0.002	-0.232 ± 0.719	0.225 ± 0.005	0.528 ± 0.004	-1.717 ± 1.211
	(111)	I	0.076 ± 0.012	0.986 ± 0.009	7.984 ± 8.847	-0.064 ± 0.015	0.743 ± 0.011	30.877 ± 13.435
		II	0.078 ± 0.010	0.684 ± 0.007	14.208 ± 6.793	-0.070 ± 0.010	0.540 ± 0.007	24.665 ± 7.689
LAO/KTO	(001)	I	—	1.523 ± 0.005	—	—	0.733 ± 0.002	—
		II	—	1.065 ± 0.009	—	—	0.487 ± 0.003	—
	(110)	I	-1.704 ± 0.064	4.803 ± 0.046	0.153 ± 2.084	1.702 ± 0.109	3.56 ± 0.084	-3.022 ± 3.307
		II	-1.099 ± 0.038	3.245 ± 0.028	-0.193 ± 1.956	1.128 ± 0.056	2.377 ± 0.043	-2.651 ± 2.561
	(111)	I	0.743 ± 0.072	4.875 ± 0.050	-16.443 ± 5.339	-0.396 ± 0.073	4.338 ± 0.051	4.311 ± 10.137
		II	0.566 ± 0.064	3.414 ± 0.045	3.952 ± 6.131	-0.401 ± 0.073	3.043 ± 0.050	6.736 ± 9.900
YAO/KTO	(111)	I	-0.290 ± 0.066	6.100 ± 0.047	40.774 ± 13.06	0.209 ± 0.022	4.755 ± 0.0159	53.046 ± 6.181

Supplementary Table 2 Fitting parameters of the SSP and PPP patterns. The fitting formulas used for the (001), (110), and (111) crystal surfaces are as follows: $y=|\ C_{\text{iso}}\ |^2$, $y=|\ C_{\text{ani}}\cos(2x+\varphi_0)+\ C_{\text{iso}}\ |^2$ and $y=|\ C_{\text{ani}}\sin(3x+\varphi_0)+\ C_{\text{iso}}\ |^2$. Range I: 106 ~ 108 meV. Range II: 90 ~ 120 meV.

Samples		Range	SSP			PPP		
			$\chi_{B,1}^{(2)}$	$\chi_{B,2}^{(2)}$	$\chi_{B,3}^{(2)}$	$\chi_{B,1}^{(2)}$	$\chi_{B,2}^{(2)}$	$\chi_{B,3}^{(2)}$
LAO/KTO	(001)	I	11.28	9.25	—	11.28	9.25	—
		II	7.78	6.47	—	7.78	6.47	—
	(110)	I	24.86	37.10	9.72	56.59	21.20	1.73
		II	17.21	24.80	6.38	37.91	14.42	1.16
	(111)	I	51.79	25.83	7.30	39.65	31.93	7.33
		II	36.75	17.86	5.12	26.34	23.09	5.15
YAO/KTO	(111)	I	49.67	38.51	7.80	55.04	35.81	7.79

Supplementary Table 3 The susceptibility of LAO/KTO and YAO/KTO obtained through SSP and PPP patterns. Range I: 106 ~ 108 meV. Range II: 90 ~ 120 meV. The uncertainties of the extracted parameters are omitted here.

Samples		Range	SSP			PPP		
			$\chi_{S,1}^{(2)}$	$\chi_{S,2}^{(2)}$	$\chi_{S,3}^{(2)}$	$\chi_{S,1}^{(2)}$	$\chi_{S,2}^{(2)}$	$\chi_{S,3}^{(2)}$
KTO	(001)	I	5.87	4.91	—	5.87	4.91	—
		II	4.35	3.51	—	4.35	3.51	—
	(110)	I	5.39	8.33	2.12	12.23	4.91	0.40
		II	3.86	5.53	1.41	8.20	3.36	0.32
	(111)	I	9.17	5.60	1.20	7.65	6.36	1.20
		II	6.71	3.75	0.89	5.13	4.55	0.89

Supplementary Table 4 The susceptibility of KTO obtained through SSP and PPP patterns. Range I: 106 ~ 108 meV. Range II: 90 ~ 120 meV. The uncertainties of the extracted parameters are omitted here.

Samples		Range	SSP			PPP		
			f_1	f_2	f_3	f_1	f_2	f_3
LAO/KTO	(001)	I	1.9	1.9	—	1.9	1.9	—
		II	1.8	1.8	—	1.8	1.8	—
	(110)	I	4.6	4.5	4.6	4.6	4.3	4.5
		II	4.5	4.5	4.5	4.6	4.3	3.7
	(111)	I	5.7	4.6	6.1	5.2	5.0	6.1
		II	5.5	4.8	5.8	5.1	5.1	5.8
YAO/KTO	(111)	I	5.4	6.9	6.5	7.2	5.6	6.5

Supplementary Table 5 The polarity enhancement factor. Range I: 106 ~ 108 meV. Range II: 90 ~ 120 meV. The uncertainties of the extracted parameters are omitted here.

Photon energey	$\mathcal{L}_{yyx}$	$\mathcal{L}_{yyz}$	$\mathcal{L}_{xxz}$	$\mathcal{L}_{zxx}$	$\mathcal{L}_{xzx}$	$\mathcal{L}_{xxx}$	$\mathcal{L}_{zzz}$
107 meV	0.2779	0.1645	0.2153	0.1806	0.1784	0.2420	0.1185
100 meV	0.3139	0.0414	0.0542	0.2040	0.2015	0.2733	0.0298

Supplementary Table 6 Dimensionless coefficients including Fresnel coefficients after projection to the polarization vector.

	(001)	(110)	(111)
Nominal (e·Å)	1.142	4.263	4.226
BECs (e·Å)	3.622	10.512	10.149

Supplementary Table 7 Dipole moments along the surface normal for different surfaces.

Parameter	YAO/KTO(111)	LAO/KTO(111)-Conductive	LAO/KTO(111)-Insulative	KTO(111)×10
ω_1 (meV)	108.294 ± 0.034	107.389 ± 0.082	107.546 ± 0.044	107.359 ± 0.024
Γ_1 (meV)	2.480 ± 0.033	3.203 ± 0.080	2.874 ± 0.041	2.171 ± 0.027
A_1 (meV)	19.329 ± 0.313	24.312 ± 1.116	14.369 ± 0.273	12.064 ± 0.200
φ_1 (arb.units)	0.681 ± 0.008	0.652 ± 0.007	0.627 ± 0.004	1.234 ± 0.062
ω_2 (meV)	99.607 ± 0.231	102.134 ± 0.216	100.952 ± 0.219	101.011 ± 0.208
Γ_2 (meV)	5.214 ± 0.318	3.613 ± 0.236	3.483 ± 0.261	9.617 ± 0.370
A_2 (meV)	9.259 ± 1.112	8.595 ± 1.053	2.808 ± 0.372	34.179 ± 1.553
φ_2 (arb.units)	0.427 ± 0.014	0.701 ± 0.021	0.484 ± 0.018	0.679 ± 0.051
χ_{NR} (arb.units)	2.279 ± 0.067	2.698 ± 0.042	1.775 ± 0.027	0.345 ± 0.140

Supplementary Table 8 Fitting parameters of Figure 2a in main text. The experimental spectra were fitted using the coherent superposition of two Lorentzian oscillators and a constant background, expressed as:

$$I(\omega) = \left| \frac{A_1 e^{i2\pi\varphi_1}}{\omega - \omega_1 + i\Gamma_1} + \frac{A_2 e^{i2\pi\varphi_2}}{\omega - \omega_2 + i\Gamma_2} + \chi_{NR} \right|^2$$

Supplementary References

1. Shen, Y. R. *Fundamentals of sum-frequency spectroscopy*. (Cambridge University Press, 2016).
2. Liu, X. et al. Nonlinear optical phonon spectroscopy revealing polaronic signatures of the $LaAlO_3/SrTiO_3$ interface. *Sci. Adv.* **9**, eadg7037 (2023).
3. Chen, X. et al. Orientation-dependent electronic structure in interfacial superconductors $LaAlO_3/KTaO_3$. *Nat. Commun.* **15**, (2024).
4. Shi, H. et al. Surface and bulk contributions to the second-harmonic generation in Bi_2Se_3. *Phys. Rev. B*. **94**, (2016).
5. Miller, R. C. & Spitzer, W. G. Far Infrared Dielectric Dispersion in $KTaO_3$. *Physical Review*. **129**, 94-98 (1963).
6. Ghosez, P., Michenaud, J. P. & Gonze, X. Dynamical atomic charges: The case of ABO_3 compounds. *Phys. Rev. B*. **58**, 6224-6240 (1998).
7. Geilhufe, R. M., Juričić, V., Bonetti, S., Zhu, J. & Balatsky, A. V. Dynamically induced magnetism in $KTaO_3$. *Phys. Rev. Res.* **3**, L022011 (2021).
8. Cabuk, S. Ab initio volume-dependent elastic and lattice dynamics properties of $KTaO_3$. *Physica Status Solidi (B)*. **247**, 93-97 (2010).
9. Gupta, A. et al. $KTaO_3$ —The New Kid on the Spintronics Block. *Adv. Mater.* **34**, (2022).
10. Garcia, A.B.S. et al. Effects of the Pechini's modified synthetic route on structural and photophysical properties of Eu^{3+} or Tb^{3+}-doped $LaAlO_3$. *Mater. Res. Bull.* **143**, 111462 (2021).
11. Shi, H. et al. Matrix formalism for radiating polarization sheets in multilayer structures of arbitrary composition. *Chin. Opt. Lett.* **15**, 081901 (2017).
12. Willett-Gies, T. et al. Vibrational properties of bulk $LaAlO_3$ from Fourier-transform infrared ellipsometry. *Thin Solid Films* **571**, 620–624 (2014).
13. Miller, R. C. et al. Far infrared dielectric dispersion in $KTaO_3$. *Phys. Rev*. **129**, 94–98 (1963).
