# Supplementary material for: Orientational enhancement of surface phonon polarity in superconducting KTaO₃ interfaces
Source: Nat Commun. 2026 Aug 14;17:9764. doi: 10.1038/s41467-026-76343-4 (PMC13572391; doi:10.1038/s41467-026-76343-4)
Supplement: Supplementary file 2 [file 41467_2026_76343_MOESM2_ESM.pdf]

nature portfolio

Peer Review File

Orientational Enhancement of Surface Phonon Polarity in Superconducting $KTaO_3$ Interfaces

Corresponding Author: Professor Wei-Tao Liu

This file contains all reviewer reports in order by version, followed by all author rebuttals in order by version.

Version 0:

Reviewer comments:

Reviewer #1

(Remarks to the Author)
In this manuscript, the authors investigate orientation-dependent surface phonon polarity in superconducting KTaO_3-based interfacial systems using surface-sensitive sum-frequency generation spectroscopy. By systematically comparing different crystallographic orientations [(111), (110), and (001)] of KTaO_3 surfaces and their corresponding amorphous LAO and YAO heterointerfaces, the study reveals a pronounced enhancement of a surface phonon mode whose effective polarity strongly depends on crystallographic orientation and closely follows the reported superconducting transition temperature T_c(~2 K for (111), ~0.9 K for (110), and absent for (001)). The work is timely and addresses an important open question in oxide-interface superconductivity, the microscopic origin of the striking orientation dependence observed in KTaO_3 based 2DEG.

While previous ARPES and transport studies (Nat Commun 15, 7704 (2024)) have suggested a connection between crystallographic orientation, electron–phonon coupling, and superconductivity, the present work is novel in that it provides direct, surface-specific spectroscopic evidence linking symmetry-breaking lattice dynamics to superconducting interfaces. Overall, we find the manuscript to be of high quality, with a well-reasoned analysis and discussion supported by an impressive body of experimental data. We would appreciate further clarification from the authors regarding the broader significance and general interest of these results. Addressing the following questions would strengthen the case for publication.

Q1: The manuscript highlights that the phonon polarity follows the trend (111) > (110) >> (001) and appears to track the superconducting transition temperature T_c. However, while the (001) interface shows an approximately fourfold enhancement in phonon polarity, it remains non-superconducting. It would be helpful for the authors to clarify why a finite interfacial polarity alone is insufficient to induce superconductivity in this case. This observation raises the question of whether a minimum (threshold) phonon polarity is required for pairing, or whether additional factors at the (001) interface act to suppress superconductivity despite the enhanced polarity.

Q2: The authors demonstrate that the LO4 phonon SFG signal correlates with the superconducting transition temperature T_c; however, a causal relationship is not fully established. It would be important to clarify that SFG measurements reveal correlations rather than direct evidence of causality. For example, insulating LAO/KTO(111) with a thin LAO layer (1.2 nm) exhibits a weak LO4 signal, whereas conducting LAO/KTO(111) with a slightly thicker LAO layer (2 nm) shows a much stronger LO4 response. This comparison suggests that the presence of the 2DEG itself may enhance the LO4 polar response, rather than the phonon independently driving superconductivity. As a result, it remains unclear whether the strong LO4 signal primarily originates from crystallographic orientation and symmetry, from interfacial charge density/carrier accumulation, or from a combination of both effects.

Q3:The authors introduce a “polarity enhancement factor” defined as the ratio of χ^((2))values between different interfaces. Since χ^((2))is extracted from fits to the SFG spectra, it would be helpful to clarify in the main text how these fits were performed, including how the non-resonant background was treated, and to report the associated uncertainties. In addition, it would be useful to comment on whether laser-induced heating or possible sample degradation could influence the SFG signal (χ^((2))values). Confirmation that the laser pulses do not significantly modify the sample (for example, through repeatability checks or by ruling out photocarrier effects) would strengthen the reliability of the extracted polarity enhancement factors.

Q4: The report states that "through susceptibility tensor analysis, we quantify the surface phonon polarity at various KTO crystallographic orientations," but it is not clearly explained how the raw SFG intensity is translated into a quantitative measure of phonon polarity or polar field. Clarifying the steps that connect the measured SFG response to the extracted polarity, such as the underlying assumptions, tensor components involved, and normalization procedures, would greatly improve the transparency and interpretability of this analysis.

Q5: The surface or interfacial layer always breaks the symmetry. Did the authors verify that the LAO (AlO_x) capping layer itself does not introduce any resonant features in the 90–120 meV energy range? In addition, could IR absorption or reflectivity of the LAO layer modify the local electric field at the KTO surface and thereby influence the measured SFG response? It would be helpful to comment on possible interference or field-modification effects arising from the thin LAO (AlO_x) cap layer.

Q6: Are the crystallographic planes (111), (110), and (001) expected to have uniform surface terminations in the as-prepared KTO substrates? For example, in Fig. 3(b), was the KTO(001) surface prepared to be purely $(KO)^{-1}/TaO_2^{+1}$ terminated? If mixed terminations or surface reconstructions are present, it would be helpful for the authors to comment on their possible existence and discuss how such effects might influence the observed phonon SFG signal.

Q7: For the UV excitation experiments, could photothermal heating have contributed to the observed response? Were any control measurements performed (for example, temporarily blocking the UV illumination on part of the sample or varying the UV power) to rule out heating-related effects?

Reviewer #2

(Remarks to the Author)
This work presents sum frequency (SF) generation studies on (001), (110), (111)-oriented $LaAlO_3/KTaO_3$ and a-$YAlO_3/KTaO_3$ heterostructures and reports more than 30 times enhancement (compared to the bare substrate) of the SF signal at around 107 meV. The authors performed susceptibility tensor analysis to explain the azimuthal anisotropy pattern and to extract polarity enhancement factor. The analysis seems to be of reasonable quality, and the assignment of vacancy-related mode is convincing. The 35-fold enhancement of a signal is indeed remarkable, and the Tc also happens to be higher in the orientation where the large enhancement is observed. However, this work still does not provide any reason to believe that the 107 meV phonon plays any role in mediating superconductivity. The mode could still have been just a spectator. Thus, I could recommend publication of this manuscript only when the issues below are sufficiently addressed.

1. As the authors already mentioned, the effective second-order nonlinear susceptibility is explicitly proportional to the effective symmetry-breaking polar field. Indeed, the authors also showed that the SF signal enhancement from different orientation is explained well with the dynamical polarization pattern and the dipole moments. Then, doesn't it mean that the "enhancement" of the SF signal simply tells us that the "symmetry-breaking polar field" is enhanced without necessity of other changes in e.g. electron-phonon coupling strength or transition dipole moment? Following that, can't the presence of symmetry-breaking polar field itself somehow contribute to higher Tc without any role of the phonon?

2. The frequency and spectral width of the surface phonon mode (107 meV) seem to be robust regardless of the electronic environment and orientations. If there is any such strong coupling, wouldn't the phonon lineshape have been highly sensitive to local field or formation of 2D EG?

3. Also, the anisotropic patterns are well explained by known crystal symmetry and rotational transformation given the experimental configuration. So, the role of interfacial symmetry in producing such SF signal is well explained, but its role in the Cooper pairing mechanism is not clear.

4. The SF spectra were obtained above the superconducting temperature with the single thickness. Can they compare the temperature dependence, or thickness dependence where they can correlate the electronic behavior and phonon-originated SF behavior?

5. The authors mentioned that their findings can "facilitate the establishment of a unified framework" for phonon-mediated pairing from the "fragmented understandings". However, this work still seems to be not well connected with the electronic degrees of freedom. It would be great if they can strengthen the discussion of "the roles played by crystalline symmetry, phonon dynamics, and interfacial polarity in the Cooper pairing mechanism".

Reviewer #3

(Remarks to the Author)
I co-reviewed this manuscript with one of the reviewers who provided the listed reports. This is part of the Nature Communications initiative to facilitate training in peer review and to provide appropriate recognition for Early Career Researchers who co-review manuscripts.

Version 1:

Reviewer comments:

Reviewer #1

(Remarks to the Author)
I would like to thank the authors for their careful and thorough response to my previous comments, as well as for the substantial revisions made to both the main text and the Supplementary Information. The authors have addressed the key concerns from the initial review in a thoughtful and constructive way, and the work now presents a significantly stronger case for the importance of orientation-dependent surface phonon polarity in superconducting KTaOs-based interfaces. The revised manuscript is now much clearer, more rigorous, and overall far more compelling in communicating its findings.

Overall, I have no further questions or comments and support the publication of the manuscript in its revised form.

Reviewer #2

(Remarks to the Author)
The authors have significantly improved the manuscript by addressing my previous concerns regarding the physical origins of the observed SFG intensity enhancements. However, while the manuscript now provides a compelling empirical correlation between interfacial symmetry breaking and the superconducting transition temperature (Tc), several physical and logical interpretations in the manuscript remain speculative or conflate distinct physical phenomena. I advise that the authors address the following conceptual points to clarify the manuscript's claims before publication:

1. The authors must decouple the static interfacial field (that drives the SFG signal) from the dynamic polar field (associated with Fröhlich-type EPC). The claim that a static surface field directly enhances the bulk Fröhlich matrix element is physically unsupported and should be removed or clarified.

2. The interpretation of the Nb-doping data for FeSe/STO should be softened to acknowledge the presence of multiple, simultaneous perturbations (carrier density, screening, strain) that may act as common causes for both the observed SFG enhancement and the higher Tc. The manuscript should frame this as a multi-factorial correlation rather than definitive proof of a causal polar-field mechanism.

3. The authors should explicitly clarify that the technique's sensitivity to the interfacial environment is derived from the macroscopic \chi(2) intensity, whereas the robustness of the phonon lineshape acts as a control for the stability of the lattice. This distinction resolves the tension between 'sensitivity' and 'insensitivity' currently present in the text.

4. Statements attributing the pairing mechanism to specific surface phonons (e.g., Pg. 3, Ln. 38) should be rephrased to emphasize these as potential modulators of EPC rather than established contributors, aligning the text with the authors' own admission that the exact mechanism remains unclear.

Reviewer #3

(Remarks to the Author)
I co-reviewed this manuscript with one of the reviewers who provided the listed reports. This is part of the Nature Communications initiative to facilitate training in peer review and to provide appropriate recognition for Early Career Researchers who co-review manuscripts.

Version 2:

Reviewer comments:

Reviewer #2

(Remarks to the Author)
I thank the authors for overall great improvement and sufficiently addressing the concerns.

Open Access This Peer Review File is licensed under a Creative Commons Attribution 4.0 International License, which permits use, sharing, adaptation, distribution and reproduction in any medium or format, as long as you give appropriate credit to the original author(s) and the source, provide a link to the Creative Commons license, and indicate if changes were made.
In cases where reviewers are anonymous, credit should be given to 'Anonymous Referee' and the source.
The images or other third party material in this Peer Review File are included in the article's Creative Commons license, unless indicated otherwise in a credit line to the material. If material is not included in the article's Creative Commons license and your intended use is not permitted by statutory regulation or exceeds the permitted use, you will need to obtain permission directly from the copyright holder.
To view a copy of this license, visit https://creativecommons.org/licenses/by/4.0/

We greatly thank all Reviewers for their efforts in reviewing our manuscript and providing constructive feedback that helped improve its quality. In this revision, we have additional measurements and calculations following the Reviewers' comments and revised our manuscript accordingly. Our point-to-point responses all reviewers' comments are provided below.

Reply to Reviewer's comments:

Reviewer #1's comments:

In this manuscript, the authors investigate orientation-dependent surface phonon polarity in superconducting $KTaO_3$-based interfacial systems using surface-sensitive sum-frequency generation spectroscopy. By systematically comparing different crystallographic orientations [(111), (110), and (001)] of $KTaO_3$ surfaces and their corresponding amorphous LAO and YAO heterointerfaces, the study reveals a pronounced enhancement of a surface phonon mode whose effective polarity strongly depends on crystallographic orientation and closely follows the reported superconducting transition temperature T_c(~2 K for (111), ~0.9 K for (110), and absent for (001)). The work is timely and addresses an important open question in oxide-interface superconductivity, the microscopic origin of the striking orientation dependence observed in $KTaO_3$ based 2DEG.

While previous ARPES and transport studies (Nat Commun 15, 7704 (2024)) have suggested a connection between crystallographic orientation, electron – phonon coupling, and superconductivity, the present work is novel in that it provides direct, surface-specific spectroscopic evidence linking symmetry-breaking lattice dynamics to superconducting interfaces. Overall, we find the manuscript to be of high quality, with a well-reasoned analysis and discussion supported by an impressive body of experimental data. We would appreciate further clarification from the authors regarding the broader significance and general interest of these results. Addressing the following questions would strengthen the case for publication.

Q1: The manuscript highlights that the phonon polarity follows the trend (111) > (110) >> (001) and appears to track the superconducting transition temperature T_c. However, while the (001) interface shows an approximately fourfold enhancement in phonon polarity, it remains non-superconducting. It would be helpful for the authors to clarify why a finite interfacial polarity alone is insufficient to induce superconductivity

in this case. This observation raises the question of whether a minimum (threshold) phonon polarity is required for pairing, or whether additional factors at the (001) interface act to suppress superconductivity despite the enhanced polarity.

(Our response:) We deeply appreciate the Reviewer for the positive remarks and very insightful comments. Regarding the question on threshold polarity, though we did not observe superconductivity on KTO(001) interfaces down to our base temperature (~0.1 K), Ueno *et al.* did achieve a T_c ~ 0.05 K via ionic liquid gating on the (001) surface [Ref. 31, *Nature Nanotech. 6, 408 (2011)*], which emerged above a threshold gate voltage at ~4.5 V. More recently, Chen *et al.* have reported a T_c up to ~0.25 K at the crystalline $CaZrO_3/KTaO_3(001)$ interface that was also tuned by gating [*arXiv:2507.01392*]. Although they did not observe any threshold behavior between –150 and +150 V [corresponding to the sheet carrier density (n_s) between 7.1 and $8.1\times10^{13}/cm^2$], a minimum n_s at $\sim0.78\times10^{13}/cm^2$ for superconductivity was proposed. Therefore, there probably exists a threshold interfacial field (related to phonon polarity) for pairing, and we have added the above notes to the manuscript:

> *"For the (001) sample, no transition was observed down to our base temperature limit of 0.1 K (in Ref.*[31]*, a T_c of ~ 50 mK was found)."* (Pg. 7, Ln. 139)
>
> *"For the (001) interfaces, the T_c ~ 0.05 K could only be achieved upon a threshold gate voltage* [31]*, also showcasing the importance of polarity."* (Pg. 13, Ln. 279)

On competing factors that suppress superconductivity on KTO(001)-based interfaces, several possible scenarios have been reported. Liu *et al*. proposed that the spin-orbit-coupling (SOC) under strong tetragonal symmetry in the (001) case pulled down the d_{xy} state well below the $d_{xz/yz}$ orbitals, suppressing the inter-orbital scattering channels required for pairing [Ref. 30, *Nat. Commun. 14, 951 (2023)*]. Besides, ARPES studies by Chen *et al.* found evidence of orientational e-ph coupling (EPC) between electrons and surface phonons, and showed that the coupling was the weakest on (001) [Ref. 39, *Nat. Commun. 15, 7704 (2024)*]. Here, the weak Fröhlich-type EPC itself could result from the reduced polarity of (001) surfaces. We thank the Reviewer for the comment that helps us to improve our discussion, and have added related notes as follows:

> *"This type of EPC is the weakest on (001) due to the lowest polarity, corresponding to the lowest T_c achievable."* (Pg. 14, Ln. 288)
>
> *"This may contribute to the formation of anisotropic spin textures and orbital hybridization* [56,57]*, particularly among the three t_{2g} orbitals, which could promote*

inter-orbital hopping and further stabilize electron pairing. Specifically, the tetragonal symmetry on (001) lowers the d_{xy} orbital below $d_{xz/yz}$ and suppresses inter-orbital scattering channels needed for pairing [30]." (Pg. 14, Ln. 291)

Q2: The authors demonstrate that the LO_4 phonon SFG signal correlates with the superconducting transition temperature T_c; however, a causal relationship is not fully established. It would be important to clarify that SFG measurements reveal correlations rather than direct evidence of causality. For example, insulating LAO/KTO(111) with a thin LAO layer (1.2 nm) exhibits a weak LO_4 signal, whereas conducting LAO/KTO(111) with a slightly thicker LAO layer (2 nm) shows a much stronger LO_4 response. This comparison suggests that the presence of the 2DEG itself may enhance the LO_4 polar response, rather than the phonon independently driving superconductivity. As a result, it remains unclear whether the strong LO_4 signal primarily originates from crystallographic orientation and symmetry, from interfacial charge density/carrier accumulation, or from a combination of both effects.

(Our response:) We greatly thank the Reviewer for this point and we fully agree on that. We now explicitly note in the text that the present study demonstrates a correlation rather than direct causality. And as we discussed in our response to Q1, parallel factors such as SOC and orientational EPC may also participate. To reveal the exact mechanism behind such correlation, a microscopic theory is desired that can quantitatively relate nonlinear optical susceptibilities to SOC, EPC, and other factors. Since such a theory is unavailable at this stage, we considered all these effects together as an *effective* polarity enhancement factor, which characterizes the degree of symmetry-breaking at different interfaces. We have strengthened this point in the revised manuscript following the Reviewer's suggestion as follows:

> "*Besides, the carrier accumulation caused by the surface polar field could also affect the strength of nonlinear susceptibility tensor elements. In the absence of the microscopic theory on nonlinear optical susceptibility…*" (Pg. 11, Ln. 211)
>
> "*…in clear correlation with T_c of each interface. It is noted that this correlation does not ensure causality, which awaits further analysis.*" (Pg. 12, Ln. 256)

Q3: The authors introduce a "polarity enhancement factor" defined as the ratio of $\chi^{(2)}$ values between different interfaces. Since $\chi^{(2)}$ is extracted from fits to the SFG spectra, it would be helpful to clarify in the main text how these fits were performed, including

how the non-resonant background was treated, and to report the associated uncertainties.

(Our response:) We thank the Reviewer for pointing this out. Our workflow for extracting the nonlinear susceptibility and the polarity enhancement factor is summarized below in Figure R1 (also added to SI as Fig. S10):

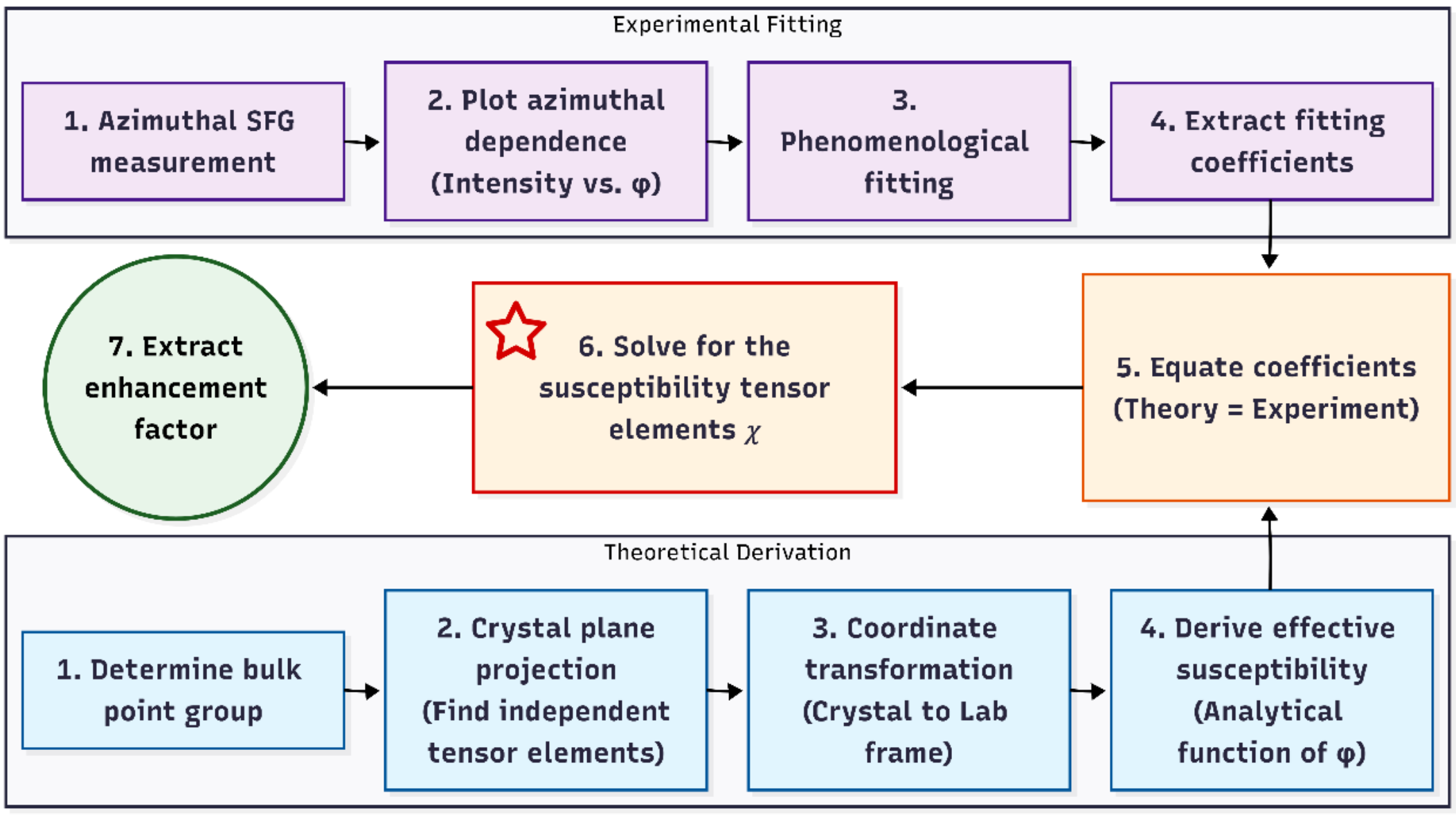

Figure R1. Workflow of the analysis of SFG data.

Briefly, we performed SFG anisotropy measurements to obtain the azimuthal angle (φ) dependent SFG spectra, then they were fitted to extract effective susceptibility tensor elements $\chi_{eff}^{(2)}$ using Eqs. S1 & S2 (purple boxes):

$$I \propto |\overleftrightarrow{\chi}_{NR} + \overleftrightarrow{\chi}_{R}|^2 = |\overleftrightarrow{\chi}_{NR} + \sum_q \frac{\vec{A}_q}{\omega_{IR} - \omega_q + i\Gamma_q}|^2$$

$$\chi_{eff}^{(2)} = [\overleftrightarrow{L}_3 \cdot \vec{e}_3] \cdot \overleftrightarrow{\chi}^{(2)} : [\overleftrightarrow{L}_1 \cdot \vec{e}_1][\overleftrightarrow{L}_2 \cdot \vec{e}_2]$$

Here, the non-resonant backgrounds ($\chi_{NR}^{(2)}$) was treated as a constant complex number independent of frequency within our spectral window, and was directly obtained via fitting, presented with uncertainties in SI Tables S1 & S8. We found the non-resonant background is generally much weaker than the resonant signal, that $|\chi_{NR}/\chi_R|$ is ~ 0.2-0.3 at the LO_4-derived phonon peak. For each polarization combination, multiple tensor elements may contribute. To extract individual tensor elements, on the theoretical side (blue boxes), we first identified the independent tensor elements of each crystal plane by projecting from the bulk symmetry group. Then, the φ-dependence of each polarization combination, contributed by various $\overleftrightarrow{\chi}^{(2)}$ tensor elements, can be derived

via a coordinate transformation from the crystal frame to lab frame. Combined (yellow boxes), we can solve for individual susceptibility tensor elements, and by comparing them in different cases, we eventually get the enhancement factor.

We have added a more detailed description to the SI (Note 2) and a concise summary in the main text as follows:

> *"The workflow for extracting the nonlinear susceptibility tensor elements is explicitly illustrated in Figure S10, with detailed derivations provided in Supplementary Note 2."* (Pg. 11, Ln. 221)

Additionally, to improve the readability of the text, we have added several subheadings, as follows:

1. *Origin of the SF response*
2. *Susceptibility tensor analysis*
3. *Orientational polarity enhancement*
4. *Discussion: polarity enhancement and superconductivity*

We hope this would make the content easier to navigate for readers, and we thank the Reviewer again for the very helpful suggestion.

In addition, it would be useful to comment on whether laser-induced heating or possible sample degradation could influence the SFG signal ($\chi^{(2)}$ values). Confirmation that the laser pulses do not significantly modify the sample (for example, through repeatability checks or by ruling out photocarrier effects) would strengthen the reliability of the extracted polarity enhancement factors.

(Our response:) We thank the Reviewer a lot for raising this important point. In our measurements, the power of both IR and NIR incident beams were carefully maintained within the region that the SF intensity scaled linearly with the laser power that $I_{SF} \propto |\overleftrightarrow{\chi}^{(2)}_{sample}|^2 I_{NIR} I_{IR}$, as shown below in Fig. R2:

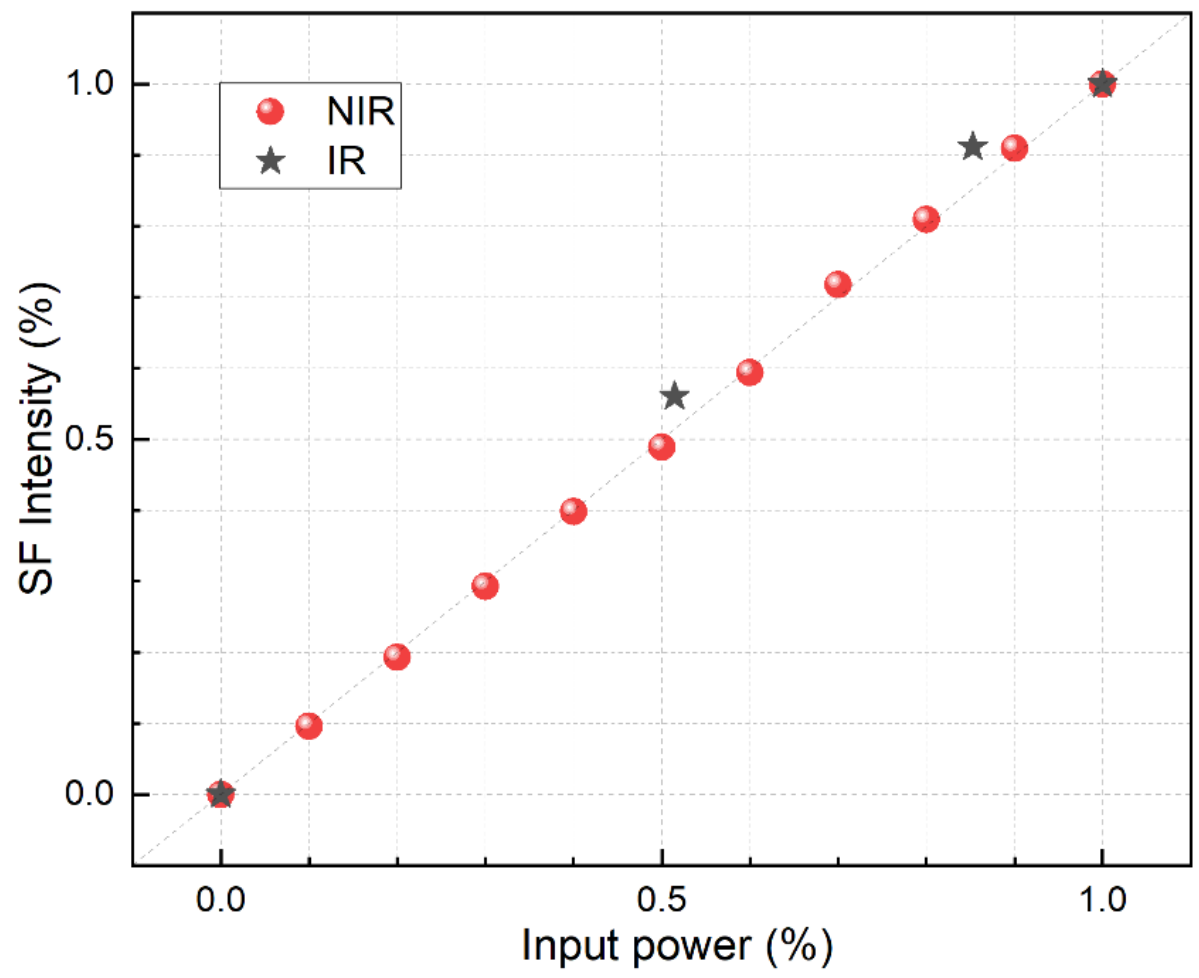

Figure R2. Laser power dependence of the SFG signal. Both IR and NIR powers are normalized to corresponding maximum values.

Within this linear regime, we assured that the sample susceptibility remained unchanged, thus any laser-induced sample modification could be neglected. If such modification happened, the emitted signal would deviate from this trend. Also, we routinely check the reproducibility of SF spectra over the data acquisition period to rule out long-term sample degradation. We thank the Reviewer again for the suggestion, and have added the power dependence data and related descriptions to the revised manuscript and SI Fig. S11:

> *"Meanwhile, the linear dependence on both IR and NIR powers confirmed that the SF signal originated from second-order nonlinear responses, with negligible effects from laser-induced heating or sample degradation (Supplementary Fig. S11). The reproducibility of SF spectra over the data acquisition period was also routinely checked to rule out long-term sample degradation."* (Pg. 8, Ln. 148)

Q4: The report states that "through susceptibility tensor analysis, we quantify the surface phonon polarity at various KTO crystallographic orientations," but it is not clearly explained how the raw SFG intensity is translated into a quantitative measure of phonon polarity or polar field. Clarifying the steps that connect the measured SFG response to the extracted polarity, such as the underlying assumptions, tensor components involved, and normalization procedures, would greatly improve the transparency and interpretability of this analysis.

(Our response:) We thank the Reviewer for noting this. As we described on the Pg. 12, Ln. 248, the polarity enhancement factor was defined by the $\overleftrightarrow{\chi}^{(2)}$ ratio between that of

heterointerfaces and corresponding KTO bare surfaces. The extraction of related $\overleftrightarrow{\chi}^{(2)}$ elements is now described with more details (both in main text and SI), as mentioned in our response to Q3. Regarding the Reviewer's specific comments:

- *Underlying assumptions:* our primary assumption is that the intrinsic third-order susceptibility of the crystal lattice $\overleftrightarrow{\chi}_B^{(3)}$ remains constant. Due to the absence of microscopic theory explicitly linking EPC and spin-orbit coupling to the optical nonlinear susceptibilities, we bundled possible causes of the observed susceptibility magnitude change into an effective polarity field, as described in Pg.11, Ln. 223 and Pg. 12, Ln. 234.
- *Tensor components:* the tensor components were provided on Pg. 11, Ln. 228., and described in SI (Note 2) with more details.
- *Normalization procedures:* the raw SFG spectra were normalized against those from a gold thin film taken under identical conditions, as the optical dispersion of gold is negligible within the experimental IR frequency range. We have added related notes to Methods session of the main text as follows and more details to SI (Note 1).

  > *"The raw SFG spectra were normalized against those from a gold thin film taken under identical conditions, as the optical dispersion of gold is negligible within the experimental IR frequency range."* (Pg. 17, Ln. 349)

We thank the Reviewer again for the comment that helps us improve the transparency and readability of our analysis.

Q5: The surface or interfacial layer always breaks the symmetry. Did the authors verify that the LAO (AlO_x) capping layer itself does not introduce any resonant features in the 90–120 meV energy range? In addition, could IR absorption or reflectivity of the LAO layer modify the local electric field at the KTO surface and thereby influence the measured SFG response? It would be helpful to comment on possible interference or field-modification effects arising from the thin LAO (AlO_x) cap layer.

(Our response:) We thank the Reviewer for noting this. According to previous IR studies [Ref. 49, *Mater. Res. Bull. 143, 111462 (2021)*], LAO does not have resonance features between 90 and 120 meV (726 ~ 968 cm^{-1}) (yellow shaded region in Figure R3).

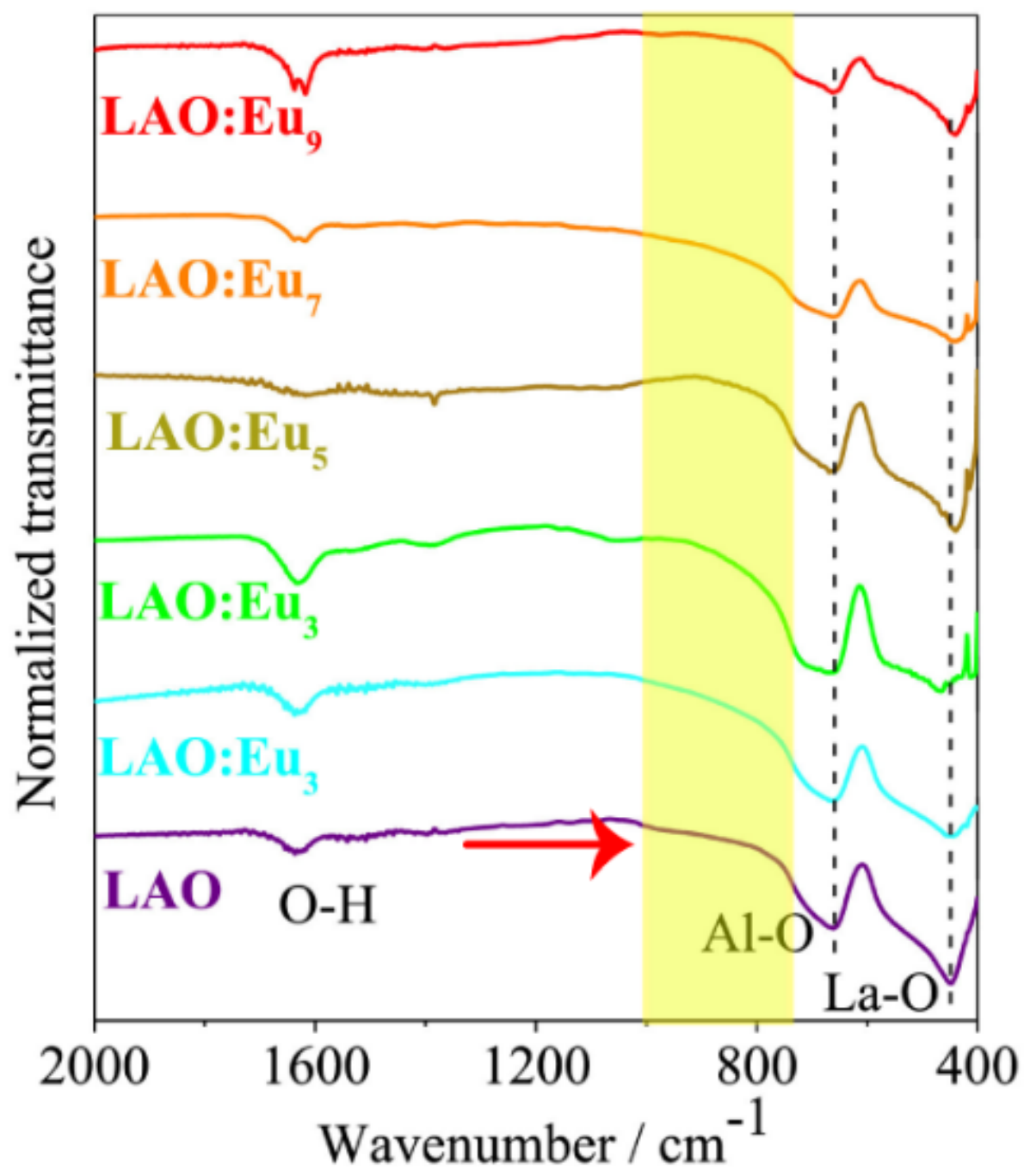

Figure R3. IR transmittance of LAO crystals.

In addition, we have evaluated the IR Fresnel coefficients of the heterostructure. Figure R4 presents the calculated field ratio between the Fresnel coefficients from the bare KTO surface (air/KTO, with the field denoted as E_{Bare}) and the LAO-capped KTO surface (air/LAO/KTO, with the field denoted as E_{Film}), with interference and absorption taken into account [Ref. 11 in SI, *Chin. Opt. Lett. 15, 081901 (2017)*]. The optical constants of LAO and KTO were taken from [Ref. 12 in SI, *Thin Solid Films 571, 620–624 (2014)*] and [Ref. 13 in SI, *Phys. Rev. 129, 1(1963)*]. Based on the extinction coefficient of LAO (left axis), the maximum optical absorption of a 2 nm-thick LAO film is ~0.35%, and the $E_{\text{Film}}/E_{\text{Bare}}$ ratio is very close to 1 (right axis) within our frequency range. Hence optically, the influence of the LAO-capping layer is negligible to our measurements.

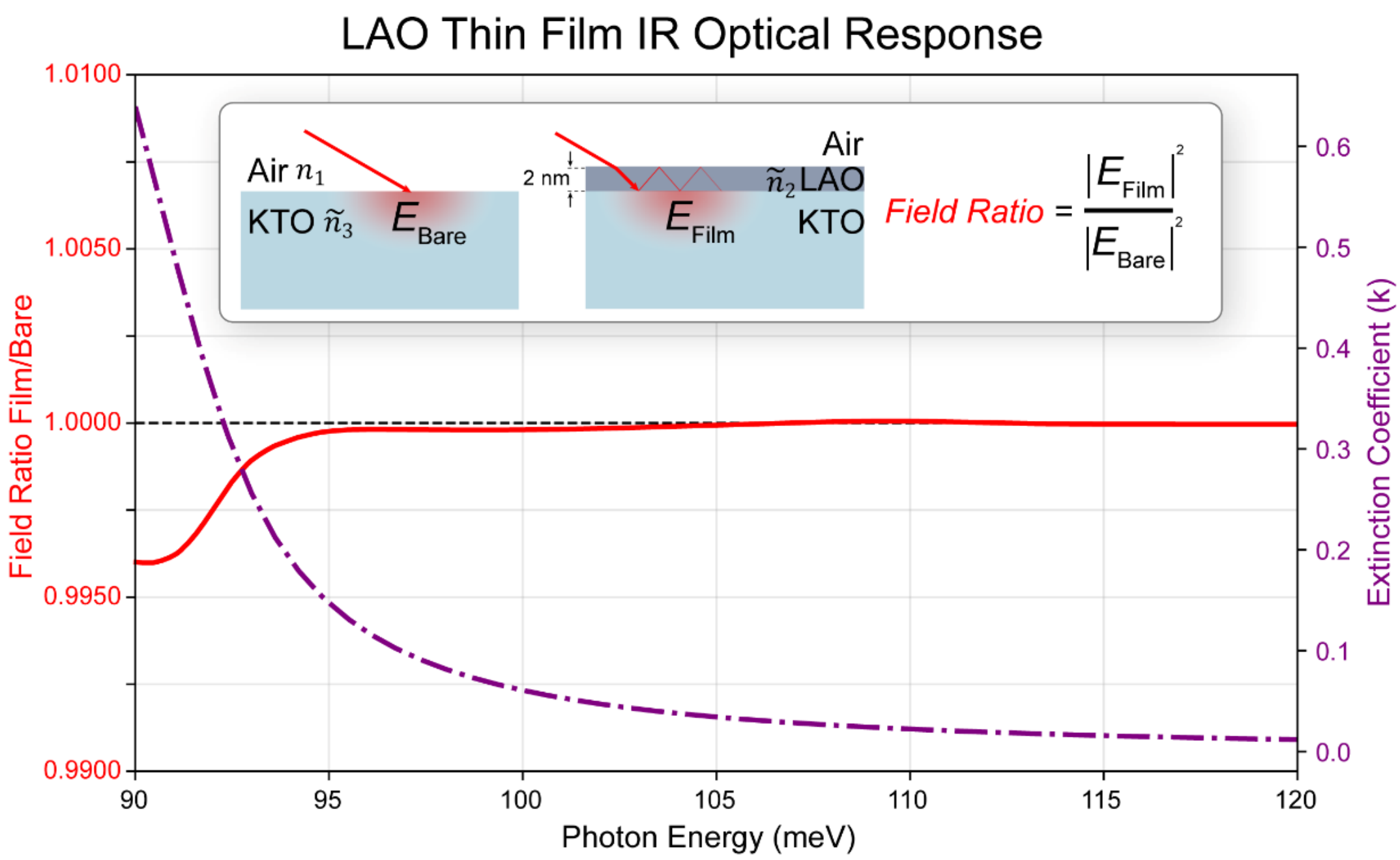

Figure R4 IR optical response of LAO film.

Also, as shown in Fig. 2a of the main text, the YAO-capped KTO sample exhibited very similar spectral features to those with LAO-capping, which further confirmed that the observed SFG response originates primarily from KTO. We thank the Reviewer again for helping us improve our paper. We have added additional notes to the SI (Note 4) and the main text as follows:

> *"Since the optical dispersion of LAO within this range is small [49], the spectral modulation caused by the nm-scale LAO capping layers is negligible (details in SI Note 4, Fig. S12)."* (Pg. 7, Ln. 120)

Q6: Are the crystallographic planes (111), (110), and (001) expected to have uniform surface terminations in the as-prepared KTO substrates? For example, in Fig. 3(b), was the KTO(001) surface prepared to be purely $(KO)^{-1}/TaO_2^{+1}$ terminated? If mixed terminations or surface reconstructions are present, it would be helpful for the authors to comment on their possible existence and discuss how such effects might influence the observed phonon SFG signal.

(Our response:) We thank the Reviewer for this insightful comment. The commercially purchased KTO substrates underwent rigorous treatment protocols by the coauthors, and the resultant high-quality surface conditions were extensively characterized in their previous publications as follows:

- The KTO (111) samples [Ref. 58, *Phys. Rev. Lett. 135, 216202(2025)*]: The substrates were annealed in a tube furnace for 2 hours at 650 °C, followed by a DI water bath at 60 °C for 30 minutes. Figure R5 presents the AFM image of the treated KTO (111) surface with a root-mean-square (RMS) roughness of ~1.4 Å. This value is consistent with the KO_3-Ta layer separation of ~1.2 Å along the <111> axis, indicating a highly uniform step-terrace structure and termination.

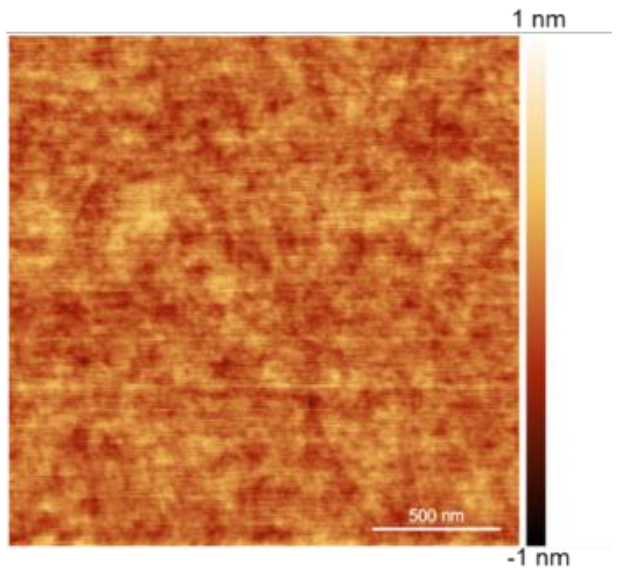

Figure R5. The AFM image of the treated KTO (111) substrate. From [Ref. 58, *Phys. Rev. Lett. 135, 216202(2025)*].

- The AlO_x/LAO/KTO samples [Ref. 33, *Phys. Rev. Lett. 126, 026802 (2021);* Ref. 39, *Nat. Commun. 15, 7704 (2024)*]: The substrates were treated at 650~680 °C in a mixed atmosphere of 1×10^{-5} mbar O_2 and 1×10^{-7} mbar H_2O vapor. The AFM images in Figure R6 (displaying the bare KTO (001), (110), and (111) substrates prior to growth) demonstrate high surface quality with RMS roughness being 2.1, 1.8, and 1.8 Å, respectively.

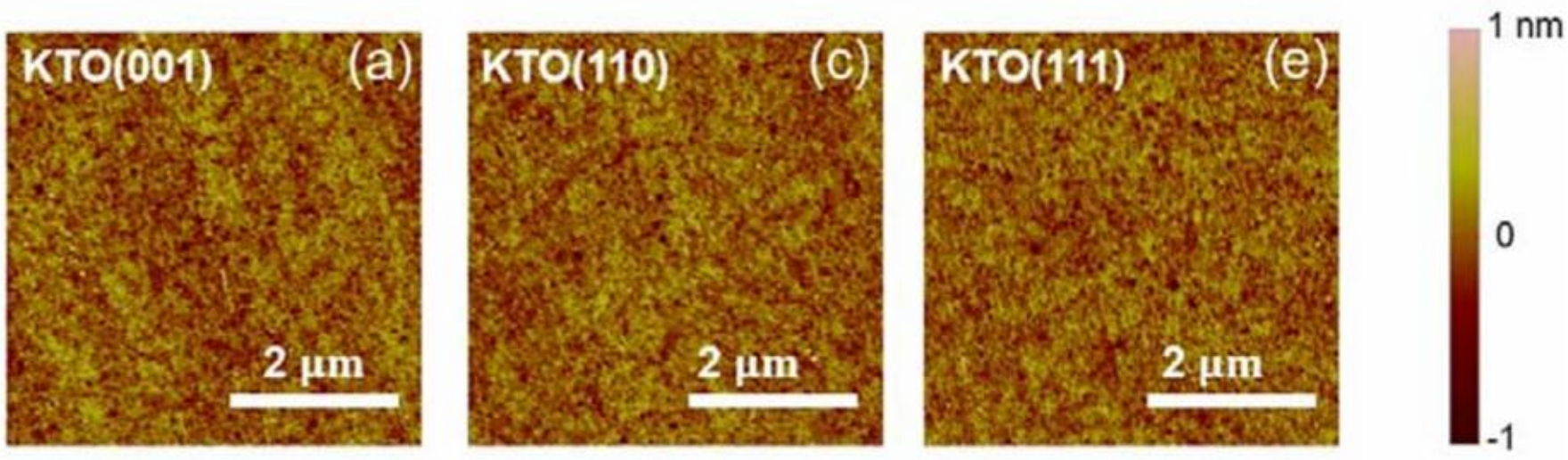

Figure R6. The AFM image of the treated KTO (001), (110) and (111) substrate. From [Ref. 33, *Phys. Rev. Lett. 126, 026802 (2021)*].

- The YAO/KTO samples [Ref. 12, *Nat. Commun. 14, 3046 (2023)*]: The substrates were annealed at 600 °C for 30 minutes in UHV to obtain a smooth surface with the RMS roughness of ~1 Å, confirmed by the AFM characterization in Figure R7.

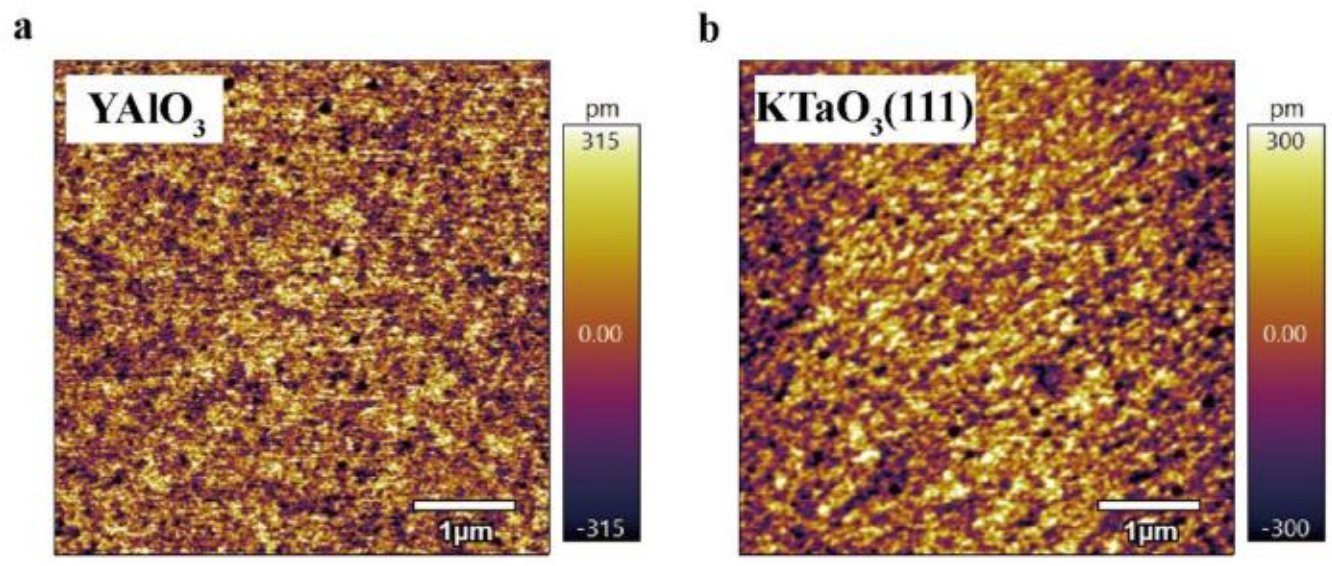

Figure R7. The AFM image of the treated YAO/KTO and (111) substrate. From [Ref. 12, *Nat. Commun. 14, 3046 (2023)*].

For the potential impact of mixed terminations, we can refer to our previous study on STO substrates [Ref. 45, *Sci. Adv. 9, eadg7037 (2023)*], in which we compared SF spectra from a purely Ti-O terminated surface and a surface with half Ti-O and half Sr-O terminations. As shown in Figure R8, the spectral line shapes remained essentially identical, and the peak intensity of the Ti-O terminated surface (red) was a bit higher, consistent with a better structure ordering. Therefore, the imperfection of sample termination will most likely reduce the absolute signal intensity, while leaving other spectral features unchanged.

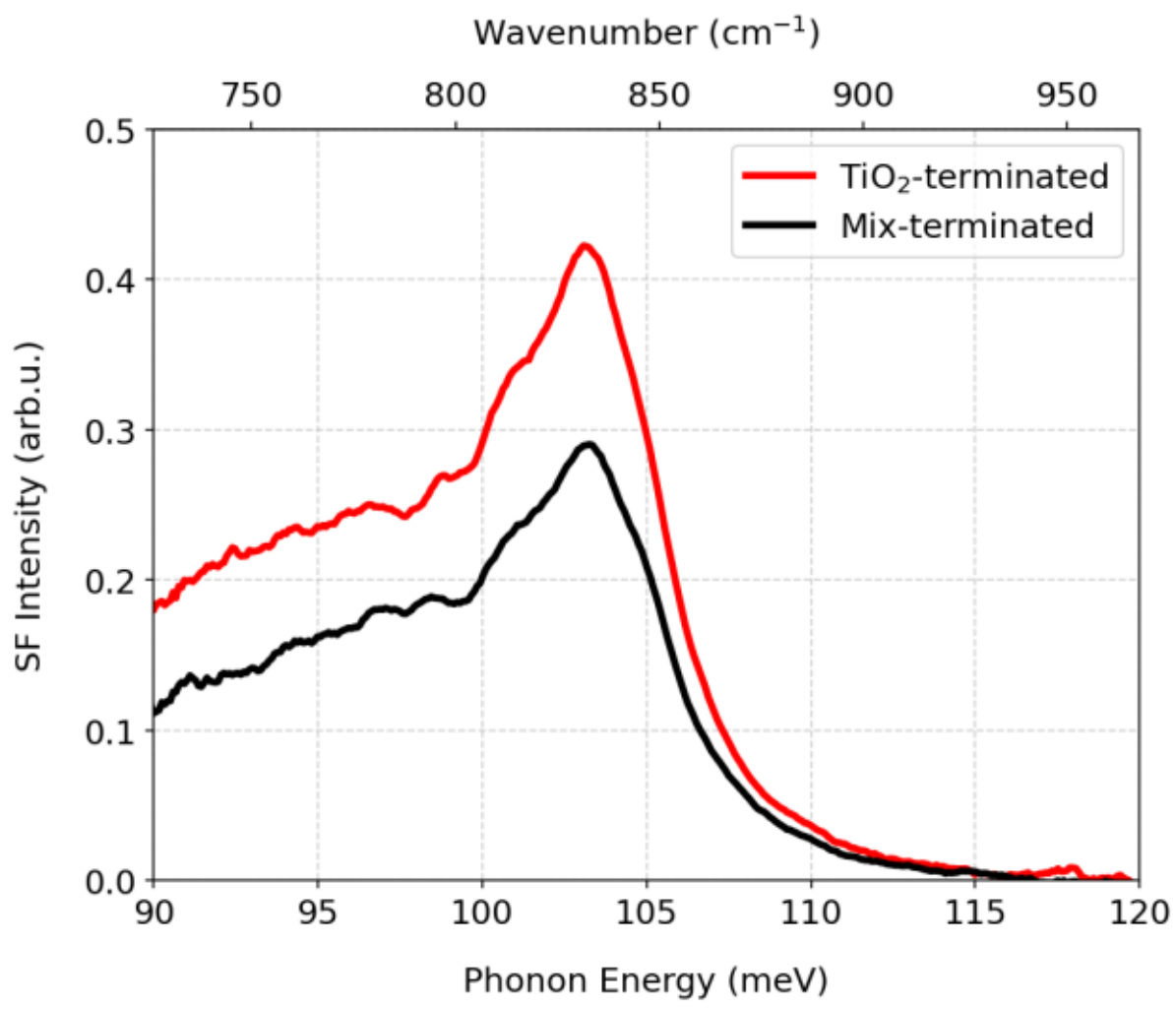

Figure R8. The SF spectra of STO with TiO_2 termination and mix termination.

Regarding possible surface reconstructions, they will lead to different bonding geometries from the bulk, including bond length, angles, and strengths, and give rise to phonon peaks distinct from bulk counterparts (e.g., as we previously observed on TiO_2 and SiO_2 surfaces [*Sci. Adv. 2, e1601162 (2016); Nat. Chem. 17, 167 (2025)*]). While in the present study, we did not observe such marked distinctions, except for the UV-

induced oxygen vacancy (V_O)-related local modes. We therefore conclude that our sample surfaces did not undergo significant reconstruction. We thank the Reviewer again for the suggestions, and have added more details and references to the manuscript as follows:

> *"In the first batch, the KTO substrates were annealed at 650 ℃ for 2 hours in air, then immersed in deionized water at 60 ℃ for 30 minutes to ensure a highly uniform step-terrace structure and surface termination. ...In the second batch, all samples featured the 1.5 nm-AlOx/1 nm-LAO/KTO hetero-structure taken from ref*[39]*. Prior to film growth, the bare KTO (001), (110), and (111) substrates were treated at 650–680 ℃ in a mixed atmosphere of 1×10^{-5} mbar O_2 and 1×10^{-7} mbar H_2O vapor. The a-YAlO3 /KTaO3(111) sample was shown from ref 12 with T_c = 2 K. For this sample, the KTO substrate was initially annealed at 600 °C for 30 minutes in an ultrahigh-vacuum environment to obtain a smooth, uniformly terminated surface prior to deposition. The rms roughness of the KTO surfaces was typically around 1~2 Å as probed by AFM* [12,33,39,58]*."* (Pg. 15, Ln. 318, in the Methods section)

Q7: For the UV excitation experiments, could photothermal heating have contributed to the observed response? Were any control measurements performed (for example, temporarily blocking the UV illumination on part of the sample or varying the UV power) to rule out heating-related effects?

(Our response:) We thank the Reviewer for this comment. We basically excluded the photothermal heating effect. For each spectrum in Fig. 2g, UV was first turned on for a designated duration and then turned off. Subsequently, we unblocked the IR and NIR beams to collect the SFG spectrum over a 5-minute period. During this 5-minute window, five consecutive SF spectra were recorded. These spectra were found to be stable, and the averaged spectrum was presented in Fig. 2g. We then proceeded to the next UV dosage. Had the photothermal effect been important, we would have observed a continuous recovery of the SF spectra during the 5-minute data acquisition time. Moreover, after the UV measurements, the sample was left in the dark for 12 hours at 60 K in UHV. We then took the SF spectrum and found it remained nearly unchanged (Fig. R9). This confirmed that the spectral changes were dominantly from changes induced by the cumulative UV exposure, rather than temporary photothermal effects.

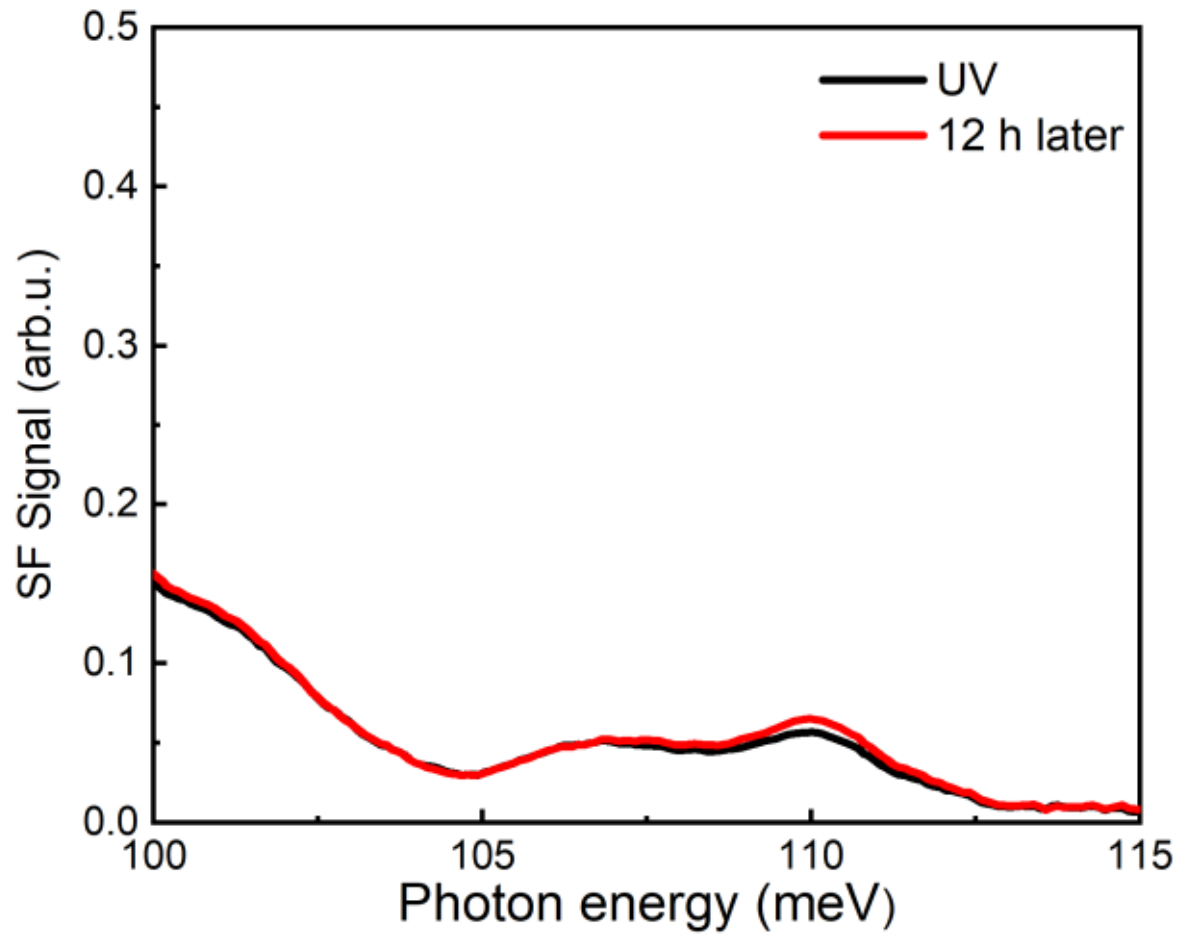

Figure R9. SF spectra of KTO measured 12 hours after UV irradiation, stored at low temperature under high vacuum.

We thank the Reviewer again for this helpful comment, and have added discussion to the revised manuscript to explicitly rule out heating-related effects as follows:

"After each irradiation period, five SF spectra were acquired sequentially over a 5-minute window with UV blocked. Stability of consecutive SF spectra ensured that the photothermal effect by UV was negligible. We then took the average of all spectra in each data acquisition window." (Pg. 9, Ln. 169)

"After the UV exposure, the dark spectrum remained stable at 100 K over 12 hours, and only gradually returned to the original state at elevated temperature or oxygen exposure (Supplementary Fig. S4), consistent with the behavior expected for interfacial V_Os." (Pg. 9, Ln. 180)

Reviewer #2 (Remarks to the Author):

This work presents sum frequency (SF) generation studies on (001), (110), (111)-oriented $LaAlO_3/KTaO_3$ and a-$YAlO_3/KTaO_3$ heterostructures and reports more than 30 times enhancement (compared to the bare substrate) of the SF signal at around 107 meV. The authors performed susceptibility tensor analysis to explain the azimuthal anisotropy pattern and to extract polarity enhancement factor. The analysis seems to be of reasonable quality, and the assignment of vacancy-related mode is convincing. The 35-fold enhancement of a signal is indeed remarkable, and the T_c also happens to be higher in the orientation where the large enhancement is observed. However, this work still does not provide any reason to believe that the 107 meV phonon plays any role in mediating superconductivity. The mode could still have been just a spectator. Thus, I could recommend publication of this manuscript only when the issues below are sufficiently addressed.

(Our response:) We sincerely thank the Reviewers for the effort in reviewing our manuscript, and we have carefully revised it based on the Reviewers' insightful comments. Before addressing the specific comments point-by-point, we wish to clarify the main perspective of our study. The emergent superconductivity at interfaces is one of the most intriguing phenomena in condensed matter physics, and the exact pairing mechanism remains a complex, open question. On KTO-based systems, recent studies have found various correlated aspects, including the surface polar field [Ref. 35, *Science 372, 721 (2021)*], orientational e-ph coupling (EPC) [Ref. 39, *Nat. Commun. 15, 7704 (2024)*], spin-orbit coupling (SOC) [Ref. 30, *Nat. Commun. 14, 951 (2023)*], among others. Our work reveal another correlated factor: the 107 meV surface phonon mode whose polarity tracks the superconducting T_c. Importantly, we did not claim that this specific phonon definitely mediates the superconductivity, and we fully agree with the Reviewers that at this stage, we cannot rule out the possibility that the mode acts as a spectator. However, because this SFG mode has an intrinsic, exceptional sensitivity to the broken symmetry, surface polarity, EPC, and SOC, it can serve as a highly effective and non-invasive probe to monitor the macroscopic physical environment that governs the emergent superconductivity, as we will elaborate below in our point-by-point replies to the comments.

Q1. As the authors already mentioned, the effective second-order nonlinear susceptibility is explicitly proportional to the effective symmetry-breaking polar field.

Indeed, the authors also showed that the SF signal enhancement from different orientation is explained well with the dynamical polarization pattern and the dipole moments. Then, doesn't it mean that the "enhancement" of the SF signal simply tells us that the "symmetry-breaking polar field" is enhanced without necessity of other changes in e.g. electron-phonon coupling strength or transition dipole moment? Following that, can't the presence of symmetry-breaking polar field itself somehow contribute to higher T_c without any role of the phonon?

(Our response:) We thank the Reviewers for this insightful comment, and we fully agree with the Reviewers that the symmetry-breaking polar field can possibly "*contribute to higher T_c without any role of the phonon*". We did not claim that this phonon definitely mediate superconductivity, and we have noted that *"this correlation does not ensure causality, which awaits further analysis"*(Pg. 12, Ln. 256) to further clarify this point. Rather, our key premise is that the impact of the polar field on the electronic properties is exquisitely reflected in the phonon properties, making SFG spectroscopy a highly sensitive probe of this environment. Overall, the polar field can modulate SF phonon signals via both direct and indirect pathways (such as EPC and SOC, among others) as follows:

- *Electrostatic polar field:* this can directly enhance the SF signal through the electric-field-induced (EFI) mechanism. We have described this on Pg. 10, Ln. 205: *"The longer penetration depth of the accompanied surface field could lead to stronger nonlinear optical responses known as the electric-field-induced (EFI) SFG from the bulk subphase* [36]*."*
- *E-ph coupling (EPC):* for the Fröhlich-type of EPC, it is inseparable from and mediated through the macroscopic polar field accompanied by the LO phonon, and the coupling matrix element scales proportionally with the polar field strength [*Many-Particle Physics, 3rd ed., Section 1.3.5*]. Therefore, an enhanced polar field usually implies an enhanced Fröhlich-type EPC, which was proposed to enhance Cooper pairing at STO-based interfaces [Ref. 20, *Chin. Phys. B 24, 117405 (2015)*]. We have described this on Pg. 3, Ln. 38: *"The inversion symmetry breaking at polar interfaces provides additional tunability by allowing giant surface fields (~10^9 V/m)* [23-26] *which could not only amplify SOC through relativistic effects, but also modulate EPC through surface phonons that contribute to the Cooper pairing* [27]*."* In the recent ARPES study by our coauthors, evidence of orientational EPC was found in LAO/KTO heterostructures [Ref. 39, *Nat. Commun. 15, 7704*

(2024)], being the strongest on (111) and weakest on (001), in line with our SFG spectroscopy results.

- *Spin-orbit coupling (SOC)*: a surface polar field can contribute to the Rashba-type of SOC, that could also modulate nonlinear optical susceptibilities via related transition matrix elements. Currently, quantitative theories are lacking, but we did observe experimental manifestations. In our recent collaborative study [*arXiv:2511.15526*, (under review)] we monitored a series of $LaAlO_3/KTaO_3$ (111) samples with T_c ranging from ~1.2 K to 2.1 K. There, we observed markedly different SFG phonon anisotropies, with samples of higher T_c exhibiting much stronger trigonal components (Fig. R10, more trigonal component corresponds to a more six-fold-like pattern). That suggests the trigonal crystal field played a role in enhancing T_c, which would further modulate SOC from the bulk phase.

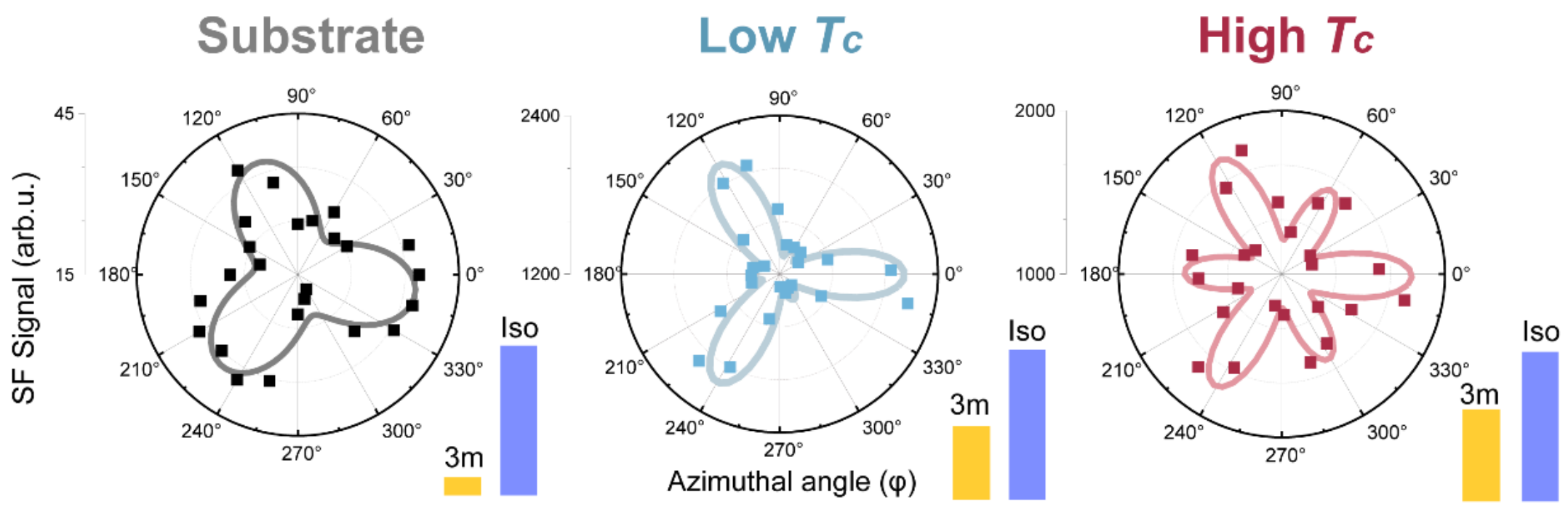

Figure R10. Anisotropy patterns of the 107 meV surface phonon mode for LAO/KTO (111) samples with varying T_c. From [*arXiv:2511.15526*].

Figure R11 below summarizes our overall scenario: the crystal symmetry and interfacial polarity directly affect the SF phonon response; meanwhile, they also determine the SOC and EPC at the interface, through which the SF phonon response can be further modulated. As all above factors may have impact on the emergent superconductivity, the SF surface phonon spectroscopy can serve as a sensitive *in situ* and non-invasive probe for monitoring related effects.

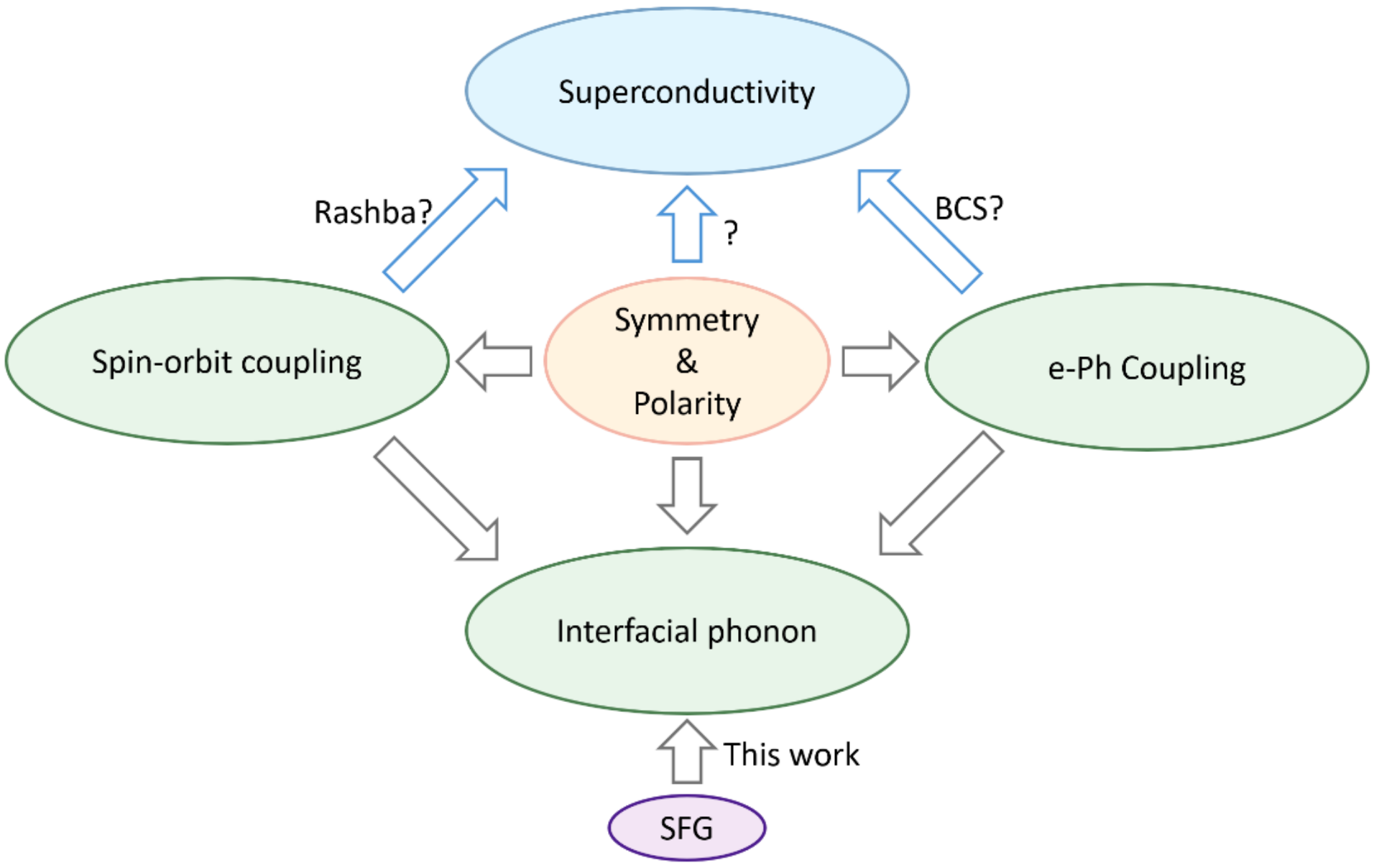

Figure R11 Microscopic physical picture of interfacial phonons and many-body coupling effects probed by SFG.

We thank the Reviewers again for this insightful comment, and have added related notes as follows:

"Meanwhile, the rehybridization of electronic states due to the broken symmetry, and symmetry-regulated EPC and/or SOC [8-10]*, could modulate the transition matrix elements of the anti-Stokes Raman process in SFG (Fig. 1b)* [45]*. Besides, the carrier accumulation caused by the surface polar field could also affect the strength of nonlinear susceptibility tensor elements. In the absence of the microscopic theory on nonlinear optical susceptibility involving above effects, we are not able to resolve individual contributions at this stage. Still, all these effects could be considered together as an effective polarity enhancement factor, which characterizes the degree of symmetry-breaking at different interfaces for comparison."* (Pg. 10, Ln. 209)

"It is noted that this correlation does not ensure causality, which awaits further analysis." (Pg. 12, Ln. 256)

In addition, prompted by the Reviewers' comment, we further investigated the relation between phonon polarity on T_c of the STO-based system. It is known that the T_c of bulk doped STO and LAO/STO interfaces typically ranges from 0.2 to 0.4 K [Ref. 6, *Science 317, 1196 (2007); Nature 456, 624 (2008); Phys. Rev. 163, 380 (1967)*], that of bulk FeSe is 8 K [*PNAS 105, 14262 (2008)*]. By growing on undoped-STO substrate, the onset FeSe-T_c on bare STO increases sharply to 40 K [*Chin. Phys. Lett. 31, 017401*

(2014)], and that on Nb-doped STO substrate could reach up to ~100 K [*Nat. Mater. 14, 285 (2014)*]. Accordingly, we found that compared to the bare STO substrate, the SF phonon intensity of Nb-doped STO substrate was enhanced by nearly 10 times (Fig. R12). This serves as another powerful example demonstrating our main perspective: the interfacial symmetry-breaking polar field that dramatically enhances superconductivity also strongly manifests in the SFG macroscopic response, highlighting the capability of our technique in probing such crucial interfacial parameters.

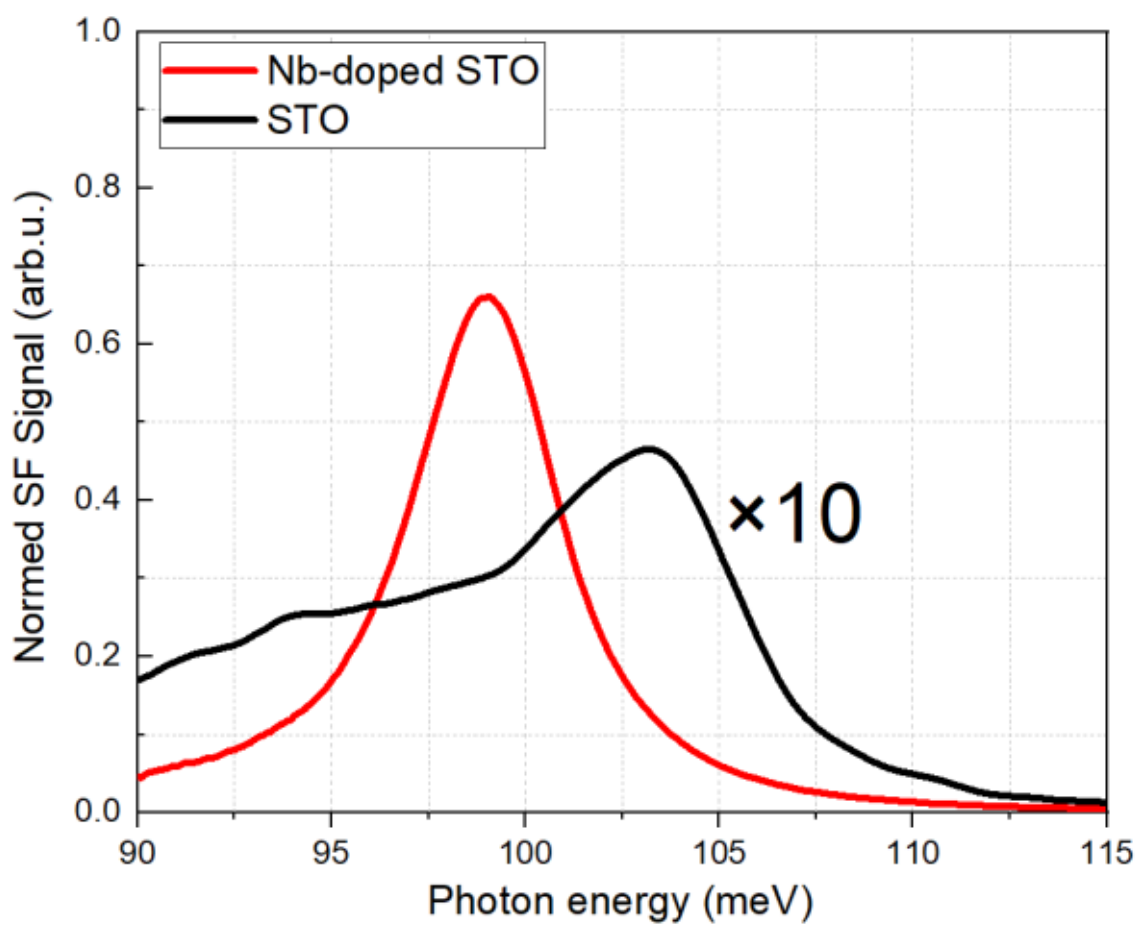

Figure R12. SF surface phonon spectra of STO (dark) and Nb-doped STO (red).

Q2. The frequency and spectral width of the surface phonon mode (107 meV) seem to be robust regardless of the electronic environment and orientations. If there is any such strong coupling, wouldn't the phonon lineshape have been highly sensitive to local field or formation of 2DEG?

(Our response:) We thank the Reviewers for this important remark. This insensitivity is mainly because the SF signal originates from both the surface and sub-surface region where the inversion symmetry was effectively broken. As shown by recent ARPES studies, the 2DEG at the KTO interfaces has a quasi-three-dimensional character, penetrating into the KTO bulk-subphase by ~5.5 to 6.8 nm [Pg. 10, Ln. 203; Ref. 39, *Nat. Commun. 15, 7704 (2024)*], which corresponds to ~15-20 unit cells. Meanwhile, the SFG coherence length was about ~ 30 nm, fully covering the 2DEG region. If the phonon mode is confined to the topmost lattice layers, the lattice constant, restoring force, and damping coefficient would be more sensitive to the local environment, as we observed previously on LAO/STO interfaces [Ref. 45, *Sci. Adv. 9, eadg7037 (2023)*]. There, the peak position and linewidth of STO surface phonons exhibited a more

noticeable change (2~3 meV). While for KTO, the mode is less localized, and we don't expect its profile to vary much from case to case (0.3 ~ 0.9 meV in all our samples).

Another point is that the observed SF spectra could be nicely fitted by complex Lorentzian functions (an example shown below in Fig. R13 with data in Table S8). This indicates that the dominant contribution to the linewidth is homogeneous broadening. If the extra damping caused by the environment is less than that due to the intrinsic lifetime, the spectral change would not be significant. On the other hand, the electronic environment may have significant impact on dynamic behaviors of these phonons, which could be captured by ultrafast pump-probe studies via photo-doping or transient structural perturbation, and is an excellent future research direction.

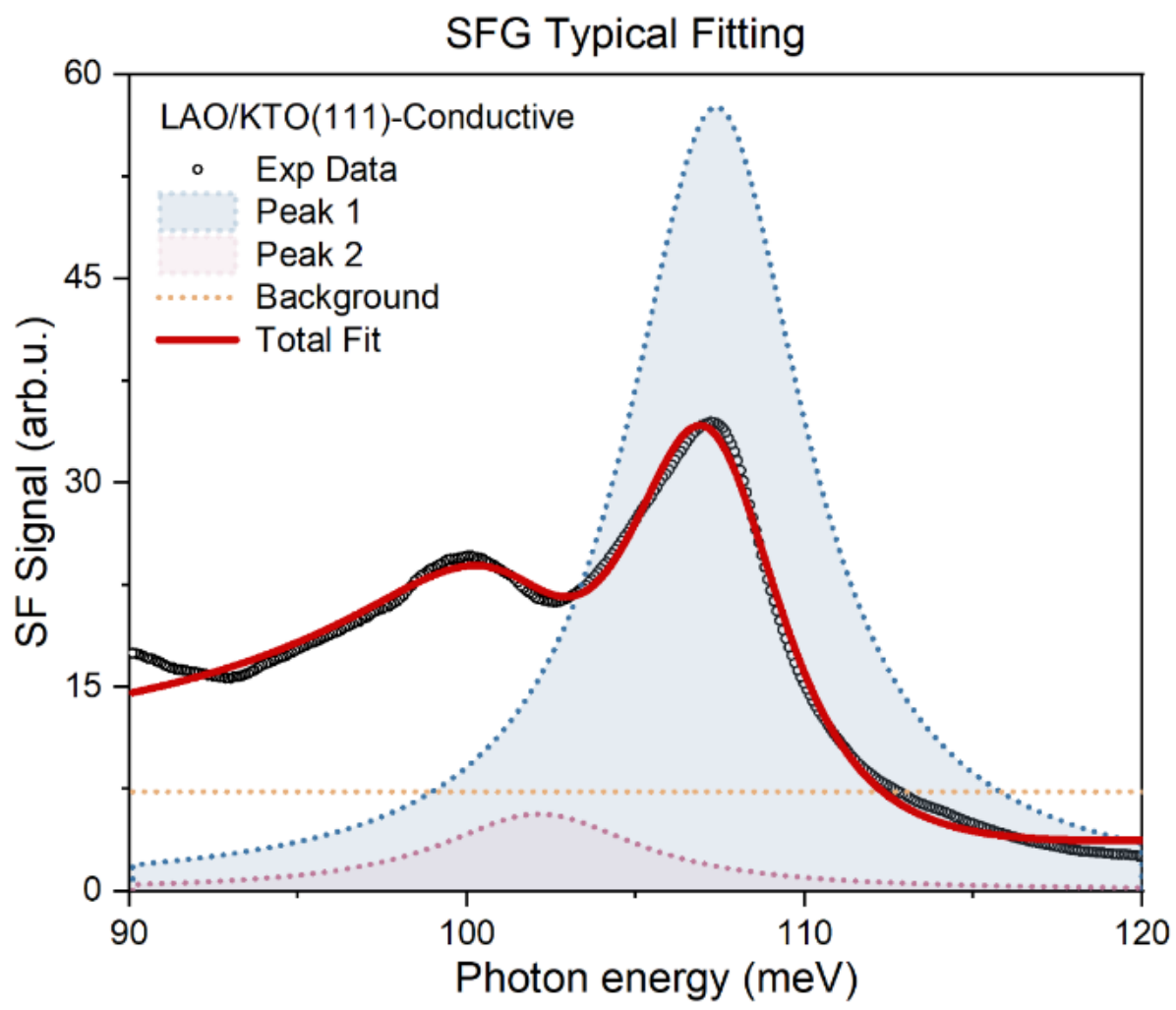

Figure R13. SF spectrum and Lorentzian fit.

We have elaborated on the discussion as follows:

> *"The longer penetration depth of the accompanied surface field could lead to stronger nonlinear optical responses known as the electric-field-induced (EFI) SFG from the bulk subphase [36]. This also explains the absence of notable frequency shift and linewidth change of the SF phonon mode from case to case."* (Pg. 10, Ln. 205)
>
> *"Future ultrafast time-resolved studies may provide more insight into the dynamic interaction between electronic and lattice degrees of freedom."* (Pg. 15, Ln. 311)

and we thank the Reviewers again for this very inspiring comment.

Q3. Also, the anisotropic patterns are well explained by known crystal symmetry and rotational transformation given the experimental configuration. So, the role of interfacial symmetry in producing such SF signal is well explained, but its role in the Cooper pairing mechanism is not clear.

(Our response:) We thank the Reviewers for noting this. Qualitatively, indeed the anisotropic patterns conformed nicely with the surface lattice symmetry, that is C_{4v}, C_{2v}, and C_{3v} for (100), (110), and (111), respectively. While quantitatively, the relative strength between tensor elements could carry more relevant information. As mentioned in our answer to Q1 above, in our recent collaborative study [*arXiv:2511.15526*], we studied LAO/KTO samples with the same (111) orientation but different T_c. Though exhibiting the same overall 3-fold symmetry, the relative strength between the trigonal and isotropic components varied systematically (Fig. R10). Physically, we propose that this originates from the interfacial trigonal crystal field that modified the SOC experienced by Ta-*5d* electrons from the bulk phase (Fig. R14). This could affect the electronic states at the Fermi surface and influence the pairing interaction, showcasing a role played by the *in-plane* interfacial symmetry via SOC.

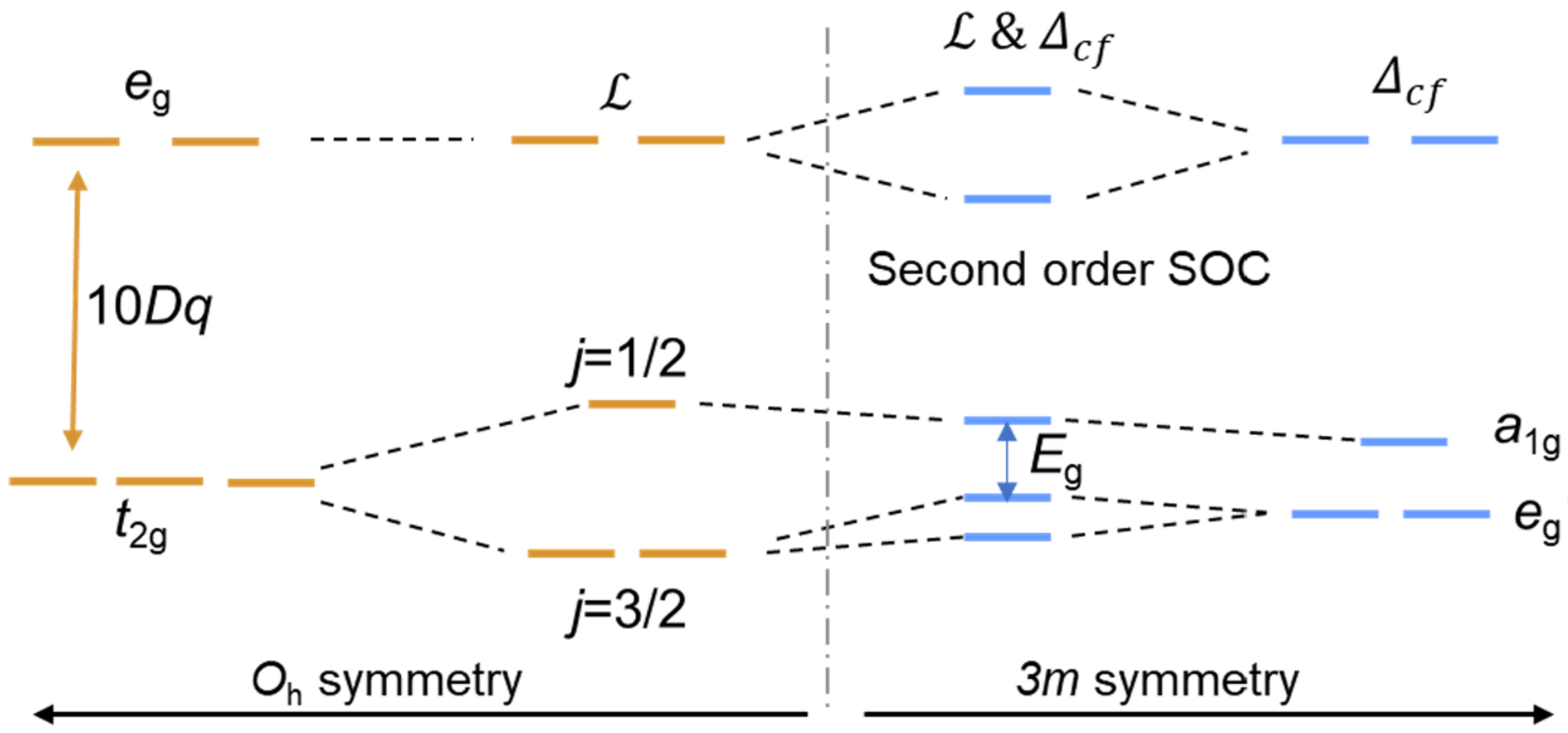

Figure R14. Hierarchical splitting of the Ta-*5d* orbitals. Left panel presents the case in bulk phase, right panel on (111) surfaces. From [*arXiv:2511.15526*].

Also, in a very recent study by our coauthors, Cao *et al.* reported a geometric scaling relation, showing an explicit linear relation between T_c and the angle θ between the (hkl) plane and the reference (100) plane [*arXiv:2512.10301*]. They suggested that increasing the angle θ [moving away from (100)] enhances the structural *asymmetry* (related to the polarity) and/or orbital overlapping, showing that the *out-of-plane* interfacial symmetry (related to polarity) could play a key role. Of course, we agree with the Reviewer that the exact pairing mechanism remains to be clarified. As our SF phonon spectroscopy can sensitively probe various aspects of the interface symmetry, both out-of-plane (polarity) and in-plane (anisotropy), it would help elucidate the microscopic role played

by symmetry. We thank the Reviewers again for this insightful comment, and have added related notes to the manuscript as follows:

> *"The inversion symmetry breaking at polar interfaces provides additional tunability by allowing giant surface fields (~10^9 V/m) [23-26] which could not only amplify SOC through relativistic effects, but also modulate EPC through surface phonons that contribute to the Cooper pairing [27]."* (Pg. 3, Ln. 38, in the Introduction section)
>
> *"Overall, the correspondence between T_c and enhanced polarity for different KTO orientations reflects the combined effects of EPC and SOC, underscoring the critical role of orientation-dependent symmetry breaking, both in-plane and out-of-plane, in devising oxide heterostructures."* (Pg. 14, Ln. 295)

Q4. The SF spectra were obtained above the superconducting temperature with the single thickness. Can they compare the temperature dependence, or thickness dependence where they can correlate the electronic behavior and phonon-originated SF behavior?

(Our response:) We thank the Reviewers for this valuable suggestion and the comparisons are as follows:

- *Temperature dependence:* we monitored spectra of KTO (001) down to 50 K as shown in SI Fig. S6. Following the Reviewer's suggestion, we added the measurement results from (110) and (111) surfaces as summarized in Fig. R15 and added in SI Fig. S6. These spectra showed a moderate, monotonic increase of intensity from room temperature, likely due to the increase of KTO dielectric constant at low temperature [*Phys. Rev. 129, 94 (1963); J. Chem. Phys. 158, 044701 (2023)*]. We did not observe any notable anomalies at lower temperatures, apart from the V_Os-related behaviors discussed in the manuscript. Although our current equipment (cryogenic system with mid-infrared optical windows) limited measurements across T_c, this continuous behavior suggests that the interfacial lattice structure is robust during cooling, providing a stable background for monitoring the behaviors at low temperature. We have added additional data and notes on the temperature dependence in the revision (Pg. 17, Ln. 353).

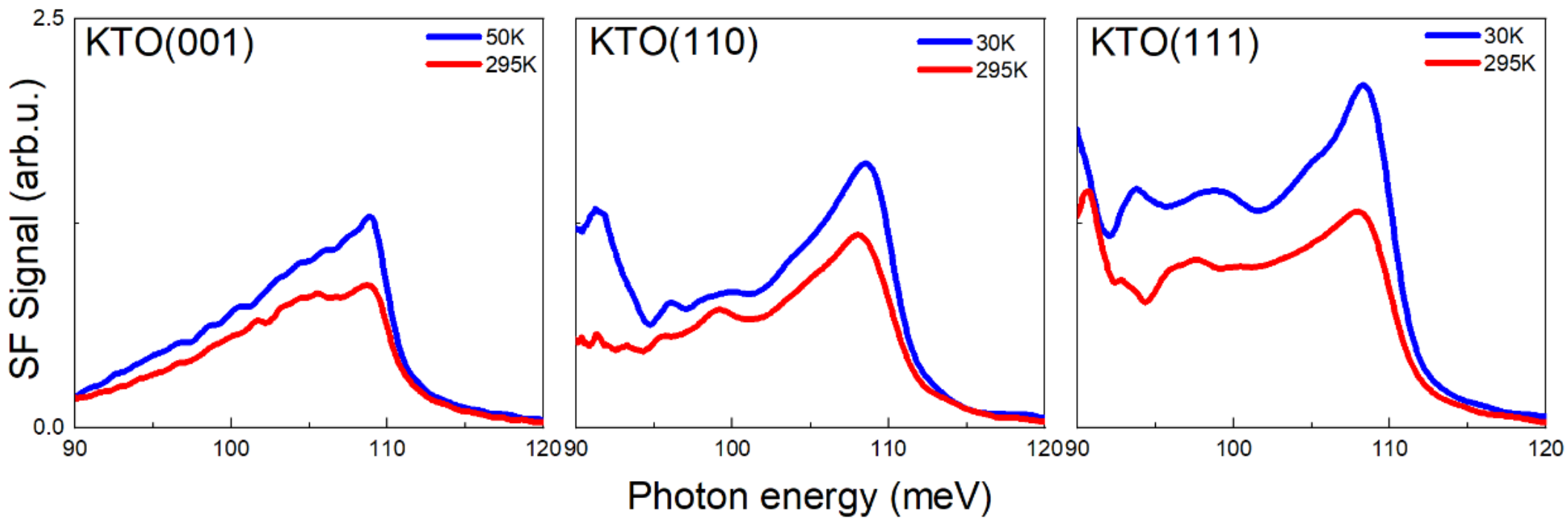

Figure R15. Spectra from KTO (001), (110), and (111) at 295 K and low temperatures.

- *Thickness dependence:* we have measured samples with different LAO overlayer thicknesses (Pg. 7, Ln. 122). On KTO (111), when the LAO thickness increased from below to above the 2DEG formation threshold, the SF intensity increased by over ~150%. For (111) samples with the 2DEG but of different LAO thicknesses, the SF phonon intensities are in general comparable (2-nm in Fig. 2a, vs. 1-nm in Fig. 2d, lower panel, also in Fig. R16).

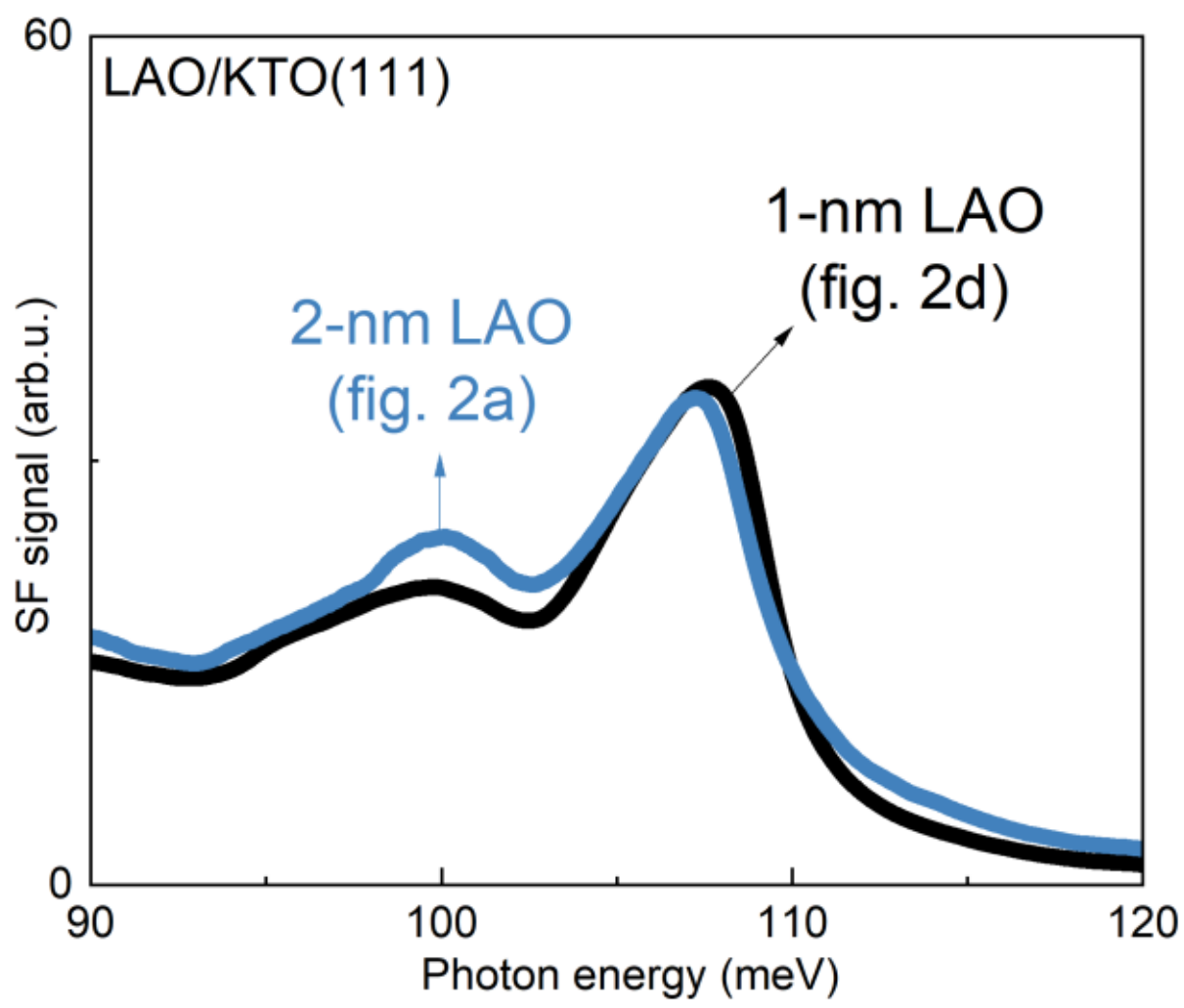

Figure R16 Comparable SF phonon intensities for (111) LAO/KTO with different LAO thicknesses (2 nm and 1 nm).

- In the collaborative study [*arXiv:2511.15526*] mentioned above, we further measured (111) samples of the same LAO thickness, but different 2DEG thicknesses (from ~3.7 to 10.5 nm), and the main difference was in the SF anisotropy patterns, showcasing a modulated in-plane symmetry sensed by the mode.

We thank the Reviewers again for the comment. We have added the supplemental temperature dependent data, and also related notes for better clarity:

> *"First, we monitored the case with different LAO thicknesses."* (Pg. 7, Ln. 122)
>
> *"For conducting (111) interfaces, 1- and 2-nm LAO capping layers yielded about the same enhancement."* (Pg. 8, Ln. 145)
>
> *"...the spectral profiles of the KTO (001), (110), and (111) surfaces were not sensitive to temperature variations (Supplementary Fig. S6) ..."* (Pg. 17, Ln. 353)

Q5. The authors mentioned that their findings can "facilitate the establishment of a unified framework" for phonon-mediated pairing from the "fragmented understandings". However, this work still seems to be not well connected with the electronic degrees of freedom. It would be great if they can strengthen the discussion of "the roles played by crystalline symmetry, phonon dynamics, and interfacial polarity in the Cooper pairing mechanism".

(Our response:) We thank the Reviewers for the suggestion. Our statement was: "*our findings can facilitate the establishment of a unified framework for correlating interfacial symmetry, phonon-mediated pairing, and defect engineering in oxide quantum materials*", and our emphasis was on finding the relationship between all above factors, which shall help establish a holistic view of such materials. To avoid confusion and overstatement, we have revised related sentences as follows:

> *"Currently, our understanding of such effects is still emerging, ..."* (Pg. 4, Ln. 54)
>
> *"Our findings can facilitate the establishment of a general framework for relating interfacial symmetry, phonon-mediated pairing, and defect engineering in oxide quantum materials."* (Pg. 5, Ln. 77)

Regarding the *electronic degrees of freedom*, our main finding was the correlation between the overall surface phonon polarity [(100) << (110) < (111)] and T_c [(100) << (110) < (111)] of LAO/KTO interfaces, and it scaled with the Born effective charge at each interface. As we noted in the answer to Q1, such polarity can have multiple effects on the electronic degree of freedom, which are as follows:

- First, it can directly enhance the Fröhlich-type EPC of zone center LO phonons that we probed. As pointed out by D. H. Lee [Ref. 20, *Chin. Phys. B 24, 117405 (2015)*], such phonons could mediate the Cooper pairing at the interfaces. For example, the ~100 meV LO phonon at the STO interface can generate an instantaneous, forward-focusing attraction that cooperatively enhances the

intrinsic Cooper pairing in FeSe, driving the anomalously high T_c.

- Second, the surface polar field can modulate SOC at the interface. For example, as we discussed in the text (Pg.14, Ln. 290), such an inversion symmetry-breaking field can lead to Rashba-type SOC at the interface, which is capable of mediating electron pairing [Ref. 56, *Phys. Rev. Lett. 87, 037004 (2001)*; Ref. 57, *npj Quantum Mater. 10, 82 (2025)*].
- Third, the confinement caused by polar field modulates the local symmetry sensed by interfacial charges. As observed in one recent collaborative study [*arXiv:2511.15526*], we observed markedly stronger trigonal components for (111) samples of higher T_c (Fig. R10), which further offsets the surface electronic levels and modifies the electron-electron interactions.
- In addition, regarding the V_O-related mode, we actually noted it to be significantly stronger in more conducting samples and at the lower temperature [see, for example, *PNAS 123, e2520089123 (2026)*].

We have summarized the above points in Fig. R11, and have added notes in Pg. 3, Ln. 38 and Pg.10, Ln. 205 to strengthen our discussion. We thank the Reviewers again for this comment.

Reviewer #3's comments:

I co-reviewed this manuscript with one of the reviewers who provided the listed reports. This is part of the Nature Communications initiative to facilitate training in peer review and to provide appropriate recognition for Early Career Researchers who co-review manuscripts.

(Our response:) We sincerely thank the Reviewers for the effort in reviewing our manuscript and provide insightful comments.

Summary of changes:

1. We have carefully refined the discussion on the relationship between surface phonon polarity and superconductivity, clarifying the roles of threshold interfacial fields, spin-orbit coupling (SOC), and electron-phonon coupling (EPC), while explicitly noting that the observed correlation does not ensure direct causality. (Pg. 2, Ln. 25; Pg. 4, Ln 54; Pg.5, Ln. 77; Pg. 7, Ln. 139; Pg. 10, Ln. 203 & Ln. 207; Pg. 11, Ln. 211; Pg. 12, Ln. 256; Pg. 13, Ln. 279; Pg. 14, Ln. 288 & Ln. 293 & Ln. 297)
2. We detailed the workflow for extracting nonlinear susceptibility tensor elements, including an illustrative flowchart and new subheadings in the main text to improve overall readability. (Pg. 11, Ln. 221; Pg. 12, Ln. 234; Pg. 17, Ln. 349; SI Notes 1 & 2; Fig. S10)
3. We added experimental data and discussions to explicitly rule out measurement artifacts from laser-induced sample degradation and UV-induced photothermal heating effects. (Pg. 8, Ln. 148; Pg. 9, Ln. 169 & Ln. 180; SI Fig. S11)
4. We incorporated additional temperature-dependent measurements across different crystallographic facets and elaborated on the LAO capping layer thickness to better correlate the electronic and phonon-originated SF behaviors. (Pg. 7, Ln. 120; Pg. 8, Ln. 145; Pg. 15, Ln. 320; Pg. 16, Ln. 326; Pg. 17, Ln. 353; SI Fig. S6 & S12)

We thank all Reviewers for their continued efforts in reviewing our manuscript and helping us improve its quality. In this revision, we have carefully re-examined the manuscript and clarified our descriptions following the Reviewers' instructions. Our point-by-point responses to all Reviewers' comments are provided below.

Responses to the Reviewers' Comments:

Reviewer #1's comments:

I would like to thank the authors for their careful and thorough response to my previous comments, as well as for the substantial revisions made to both the main text and the Supplementary Information. The authors have addressed the key concerns from the initial review in a thoughtful and constructive way, and the work now presents a significantly stronger case for the importance of orientation-dependent surface phonon polarity in superconducting $KTaO_3$*-based interfaces. The revised manuscript is now much clearer, more rigorous, and overall far more compelling in communicating its findings. Overall, I have no further questions or comments and support the publication of the manuscript in its revised form.*

(Our response:) We deeply appreciate the Reviewer's insightful comments that helped us improve the manuscript in the previous round, and sincerely thank the Reviewer for the positive assessment.

Reviewer #2 (Remarks to the Author):

The authors have significantly improved the manuscript by addressing my previous concerns regarding the physical origins of the observed SFG intensity enhancements. However, while the manuscript now provides a compelling empirical correlation between interfacial symmetry breaking and the superconducting transition temperature (Tc), several physical and logical interpretations in the manuscript remain speculative or conflate distinct physical phenomena. I advise that the authors address the following conceptual points to clarify the manuscript's claims before publication:

(Our response:) We sincerely thank the Reviewers for the insightful and constructive comments, which have helped us further improve our manuscript. We have revised the manuscript accordingly, and our point-by-point responses are provided below.

1. The authors must decouple the static interfacial field (that drives the SFG signal) from the dynamic polar field (associated with Fröhlich-type EPC). The claim that a static surface field directly enhances the bulk Fröhlich matrix element is physically unsupported and should be removed or clarified.

(Our response:) We thank the Reviewers for pointing this out. Indeed, our previous descriptions needed further clarification. We fully agree that the static field is not a component of the bare Fröhlich vertex and cannot be directly added to the phonon-induced longitudinal field. Meanwhile, it can still help modulate the interfacial EPC via indirect channels. For example, Zhao *et al*. found that positive back-gating in FeSe/$SrTiO_3$ could draw electrons closer to the interface thereby strengthening their effective coupling to $SrTiO_3$ phonons [*"Direct Imaging of Electron Transfer and Its Influence on Superconducting Pairing at the FeSe/SrTiO₃ Interface," Sci. Adv. 4, eaao2682 (2018)*]. In this revision, we have clarified this point to avoid ambiguity and added notes on possible influencing factors, as follows:

- Original statement: "*(The surface field could) modulate EPC through surface phonons that contribute to the Cooper pairing* [27]."
  Revised statement: "*Such a static field may influence the interfacial EPC landscape by modifying the equilibrium polar structure*[25,27]*, the dielectric response and carrier screening*[28,29]*, and the confinement and spatial distribution of interfacial electronic states*[30,31] *thereby changing their overlap with polar-phonon fields*[32]." (Pg. 3, Ln. 41-44)

- Original statement: "*...revealing a symmetry-breaking polar field that could amplify SOC and EPC for (111) and (110) surfaces.*"
  Revised statement: "*... revealing an orientation-dependent static interfacial polar field that can enhance Rashba-type SOC and help modulate the interfacial EPC landscape through the associated polar reconstruction.*" (Pg. 4, Ln. 73- Pg. 5, Ln. 75)

- Original statement: "*This type of EPC is the weakest on (001) due to the lowest polarity, corresponding to the lowest Tc achievable.*"
  Revised statement: "*Consistently, ARPES studies report the weakest EPC signatures on (001), where our SFG measurements also show the lowest surface polarity, suggesting a possible correlation with its low* T_c." (Pg. 14, Ln. 294-296)

2. The interpretation of the Nb-doping data for FeSe/STO should be softened to acknowledge the presence of multiple, simultaneous perturbations (carrier density, screening, strain) that may act as common causes for both the observed SFG enhancement and the higher Tc. The manuscript should frame this as a multi-factorial correlation rather than definitive proof of a causal polar-field mechanism.

(Our response:) We fully agree with the Reviewer that the Nb-doped STO comparison is not to be interpreted as a polar-field effect alone, and we mainly used it as an example showcasing a similar effect to the KTO-based system. In this revision, we have explicitly framed the correspondence between SFG-derived polarity enhancement and T_c as an empirical correlation following the Reviewers' suggestion:

- Original statement: " *Overall, the **correspondence** between Tc and enhanced polarity for different KTO orientations reflects the combined effects of EPC and SOC, underscoring the **critical** role of orientation-dependent symmetry breaking...*"
  Revised statement: "*Overall, we obtained an **empirical correspondence** between T_c and enhanced polarity for different KTO samples, which can reflect the combined effects of SOC and EPC, and underscore the **potential** role of orientation-dependent symmetry breaking....*" (Pg. 14, Ln. 302- Pg. 15, Ln. 305)

3. The authors should explicitly clarify that the technique's sensitivity to the interfacial environment is derived from the macroscopic $\chi^{(2)}$ intensity, whereas the robustness of the phonon lineshape acts as a control for the stability of the lattice. This distinction resolves the tension between 'sensitivity' and 'insensitivity' currently present in the text.

(Our response:) We thank the Reviewer a lot for pointing this out. We have now clarified this point by adding the following notes:

- "*Therefore, the robustness of the phonon frequency and linewidth from case to case indicates that the underlying lattice structures remained stable, without major structural variations and/or chemical degradation.*" (Pg. 10, Ln. 211- Pg. 11, Ln. 213)

- "*We note that the interfacial sensitivity mainly refers to changes in the SFG intensity, which scales with $|\chi^{(2)}|^2$ and probes the interfacial polar response; by contrast, the*

nearly unchanged phonon frequencies and linewidths serve as a control for lattice stability. " (Pg. 17, Ln. 358- 361)

- "*Since spectral profiles of the KTO (001), (110), and (111) surface phonons showed only minor temperature dependence (Supplementary Fig. S6), spectra discussed in the text were all taken at room temperature unless otherwise specified.*" (Pg. 17, Ln. 363-365)

- "*...the resulting normalized intensity is proportional to the squared modulus of the effective second-order nonlinear susceptibility,* $\left|\chi_{eff}^{(2)}\right|^2$. *Changes in SFG intensity therefore reflect changes in the effective interfacial nonlinear response, whereas the phonon frequencies and linewidths provide independent measures of the stability of the underlying lattice structures.*" (SI, Pg. 2, Ln. 36-40)

4. Statements attributing the pairing mechanism to specific surface phonons (e.g., Pg. 3, Ln. 38) should be rephrased to emphasize these as potential modulators of EPC rather than established contributors, aligning the text with the authors' own admission that the exact mechanism remains unclear.

(Our response:) We thank the Reviewer for pointing this out, and we fully agree that the pairing mechanism in KTO-based interfaces has not yet been established. In this revision, we have carefully rephrased our descriptions to emphasize our finding as a potential modulator, as follows:

- Original statement: "*Our findings can **facilitate** the establishment of a general framework for relating interfacial symmetry, phonon-mediated pairing, and defect engineering in oxide quantum materials.*"
  Revised statement: "*Our findings can **contribute to** the establishment of a general framework for relating interfacial symmetry, phonon-mediated electron-electron correlation, and defect engineering in oxide quantum materials.*" (Pg. 5 Ln. 81-83)

- Original statement: "*The LO_4 mode … can **dominate** the long-range electron-electron attraction…*"
  Revised statement: " *The LO_4 mode … can **mediate** the long-range electron-electron attraction…*" (Pg. 14, Ln. 286-289)

- Original statement: "*Such contrasts are intricately linked to the anisotropy of superconductivity, suggesting an intimate interplay between interfacial polarities,*

defects, and emergent superconductivity."

Revised statement: "*Such contrasts show an orientation dependence synchronizing the superconducting anisotropy, suggesting that interfacial polarity and defects are closely related to the electronic environment of KTO-based heterointerfaces.*" (Pg. 15, Ln. 316-318)

- Original statement: " *Overall, the **correspondence** between Tc and enhanced polarity for different KTO orientations reflects the combined effects of EPC and SOC, underscoring the **critical** role of orientation-dependent symmetry breaking...*"

  Revised statement: "*Overall, we obtained an **empirical correspondence** between* T_c *and enhanced polarity for different KTO samples, which can reflect the combined effects of SOC and EPC, and underscore the **potential** role of orientation-dependent symmetry breaking....*" (Pg. 14, Ln. 302- Pg. 15, Ln. 305)

Reviewer #3's comments:

I co-reviewed this manuscript with one of the reviewers who provided the listed reports. This is part of the Nature Communications initiative to facilitate training in peer review and to provide appropriate recognition for Early Career Researchers who co-review manuscripts.

(Our response:) We sincerely thank the Reviewer again for participating in the review process and helping us improve our manuscript.

Summary of changes:

1. We distinguished the static interfacial field detected by SFG from the dynamic polar field involved in Fröhlich EPC and clarified its possible indirect effects. (Pg. 3–5; Pg. 14)
2. We reframed the correlation between interfacial polarity and T_c as an empirical result reflecting combined SOC and EPC effects. (Pg. 14–15)
3. We clarified that SFG intensity reflects the interfacial response, while stable phonon frequencies and linewidths confirm lattice robustness. (Pg. 10-11; Pg. 17; SI, Pg. 2, Ln. 36-40)
4. We softened statements regarding superconducting pairing and described surface phonons as potential EPC modulators rather than established pairing mediators. (Pg. 5; Pg. 14–15)

Responses to the Reviewers' Comments:

Reviewer #2 (Remarks to the Author):

I thank the authors for overall great improvement and sufficiently addressing the concerns.

(Our response:) We sincerely thank the Reviewer for the positive evaluation of our revised manuscript and for endorsing its publication. We are deeply grateful for your rigorous review, valuable suggestions, and the time you dedicated to helping us improve this work.
